# Risk Algorithm Using Serial Biomarker Measurements Doubles the Number of Screen-Detected Cancers Compared With a Single-Threshold Rule in the United Kingdom Collaborative Trial of Ovarian Cancer Screening

**DOI:** 10.1200/JCO.2014.59.4945

**Published:** 2015-05-11

**Authors:** Usha Menon, Andy Ryan, Jatinderpal Kalsi, Aleksandra Gentry-Maharaj, Anne Dawnay, Mariam Habib, Sophia Apostolidou, Naveena Singh, Elizabeth Benjamin, Matthew Burnell, Susan Davies, Aarti Sharma, Richard Gunu, Keith Godfrey, Alberto Lopes, David Oram, Jonathan Herod, Karin Williamson, Mourad W. Seif, Howard Jenkins, Tim Mould, Robert Woolas, John B. Murdoch, Stephen Dobbs, Nazar N. Amso, Simon Leeson, Derek Cruickshank, Ian Scott, Lesley Fallowfield, Martin Widschwendter, Karina Reynolds, Alistair McGuire, Stuart Campbell, Mahesh Parmar, Steven J. Skates, Ian Jacobs

**Affiliations:** Usha Menon, Andy Ryan, Jatinderpal Kalsi, Aleksandra Gentry-Maharaj, Mariam Habib, Sophia Apostolidou, Matthew Burnell, Susan Davies, Richard Gunu, Martin Widschwendter, Elizabeth Benjamin, Mahesh Parmar, and Ian Jacobs, University College London; Anne Dawnay and Tim Mould, University College London Hospital; Naveena Singh, Barts and the London School of Medicine and Dentistry; David Oram and Karina Reynolds, St Bartholomew's Hospital; Alistair McGuire, London School of Economics; Stuart Campbell, Create Health Clinic; Aarti Sharma, University Hospital of Wales; Nazar N. Amso, Cardiff University, Cardiff; Keith Godfrey, Queen Elizabeth Hospital, Gateshead; Alberto Lopes, Royal Cornwall Hospital, Truro; Jonathan Herod, Liverpool Women's Hospital, Liverpool; Karin Williamson, Nottingham City Hospital, Nottingham; Mourad W. Seif, St Mary's Hospital, Manchester; Howard Jenkins and Ian Scott, Royal Derby Hospital, Derby; Robert Woolas, Queen Alexandra Hospital, Portsmouth; John B. Murdoch, St Michael's Hospital, Bristol; Stephen Dobbs, Belfast City Hospital, Belfast; Simon Leeson, Llandudno Hospital, Ysbyty Gwynedd; Derek Cruickshank, James Cook University Hospital, Middlesbrough; Lesley Fallowfield, University of Sussex, Falmer, United Kingdom; Steven J. Skates, Massachusetts General Hospital and Harvard Medical School, Boston, MA; and Ian Jacobs, UNSW Australia, Sydney, NSW, Australia.

## Abstract

**Purpose:**

Cancer screening strategies have commonly adopted single-biomarker thresholds to identify abnormality. We investigated the impact of serial biomarker change interpreted through a risk algorithm on cancer detection rates.

**Patients and Methods:**

In the United Kingdom Collaborative Trial of Ovarian Cancer Screening, 46,237 women, age 50 years or older underwent incidence screening by using the multimodal strategy (MMS) in which annual serum cancer antigen 125 (CA-125) was interpreted with the risk of ovarian cancer algorithm (ROCA). Women were triaged by the ROCA: normal risk, returned to annual screening; intermediate risk, repeat CA-125; and elevated risk, repeat CA-125 and transvaginal ultrasound. Women with persistently increased risk were clinically evaluated. All participants were followed through national cancer and/or death registries. Performance characteristics of a single-threshold rule and the ROCA were compared by using receiver operating characteristic curves.

**Results:**

After 296,911 women-years of annual incidence screening, 640 women underwent surgery. Of those, 133 had primary invasive epithelial ovarian or tubal cancers (iEOCs). In all, 22 interval iEOCs occurred within 1 year of screening, of which one was detected by ROCA but was managed conservatively after clinical assessment. The sensitivity and specificity of MMS for detection of iEOCs were 85.8% (95% CI, 79.3% to 90.9%) and 99.8% (95% CI, 99.8% to 99.8%), respectively, with 4.8 surgeries per iEOC. ROCA alone detected 87.1% (135 of 155) of the iEOCs. Using fixed CA-125 cutoffs at the last annual screen of more than 35, more than 30, and more than 22 U/mL would have identified 41.3% (64 of 155), 48.4% (75 of 155), and 66.5% (103 of 155), respectively. The area under the curve for ROCA (0.915) was significantly (*P* = .0027) higher than that for a single-threshold rule (0.869).

**Conclusion:**

Screening by using ROCA doubled the number of screen-detected iEOCs compared with a fixed cutoff. In the context of cancer screening, reliance on predefined single-threshold rules may result in biomarkers of value being discarded.

## INTRODUCTION

A key component of cancer control is screening, and significant research is underway to develop highly sensitive and specific tests that are minimally invasive. Circulating biomarkers have a major role in this effort. Many are not specific to the cancer because they are altered in other malignant or benign conditions. Therefore, it is essential to carefully define the cutoff for abnormality. Frequently, biomarker levels are interpreted by using a single-threshold rule developed in the context of differential diagnosis of clinically presenting cancers. Biomarker velocity, which can be significantly different in patients with cancer compared with controls^1^ is often ignored. Where it has been used, the data may be conflicting as they are for prostate-specific antigen velocity in prostate cancer^2–4^ or limited as they are for ovarian cancer.^1,5,6^ Modeling studies^5,6^ that use data from the Prostate, Lung, Colorectal and Ovarian (PLCO) Cancer Screening Trial^7^ suggest that up to a third of the ovarian cancer cases could have been detected earlier if cancer antigen 125 (CA-125) velocity had been used instead of a fixed cutoff.

In the multimodal screening (MMS) arm of the United Kingdom Collaborative Trial of Ovarian Cancer Screening (UKCTOCS), women underwent serial serum CA-125 testing.^8,9^ CA-125 velocity was interpreted by using a risk of ovarian cancer algorithm (ROCA), which compares an individual's serial profile with that of cases and controls to estimate the risk of having ovarian cancer.^10^ We report here on the impact of using CA-125 velocity compared with a single-threshold rule on ovarian cancer detection during 296,911 woman-years of annual incidence screening.

## PATIENTS AND METHODS

The trial was approved by the United Kingdom North West Multicentre Research Ethics Committee (International Standard Randomized Controlled Trial Number ISRCTN22488978 and ClinicalTrials.gov NCT00058032). Trial design, including details of recruitment and randomization, and the results of the initial (prevalence) screen have been described elsewhere.^8,9^ All women provided written informed consent.

**Fig 1.**
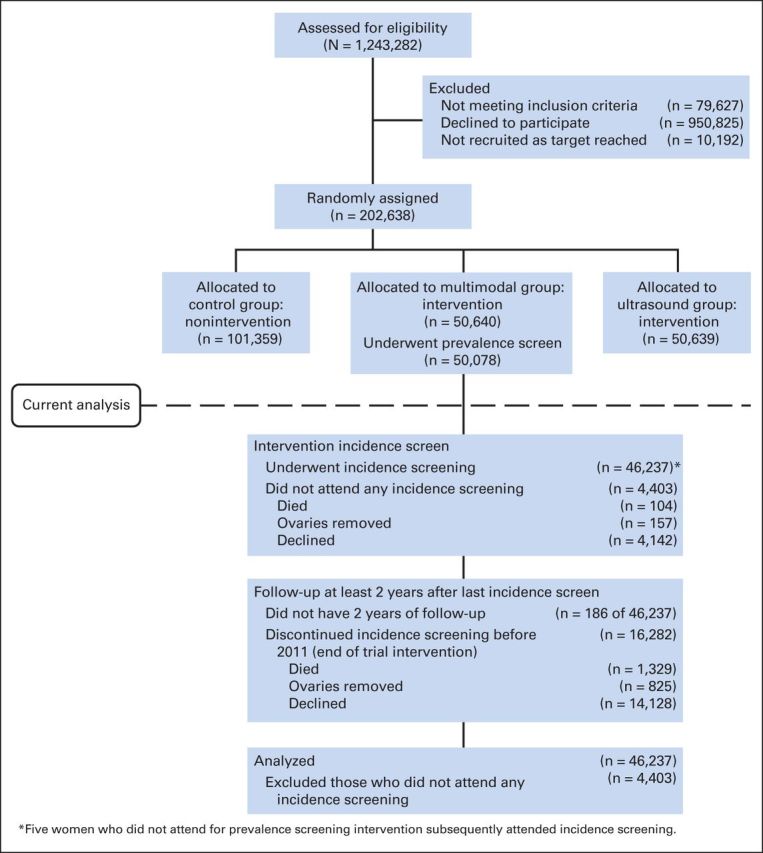
CONSORT diagram.

In brief, between 2001 and 2005, 202,638 women were randomly assigned, 50,640 of whom were allocated to the MMS group. Of those, 50,078 (98.9%) underwent a prevalence screen (Fig 1). The sensitivity, specificity, and positive predictive values (PPVs) for detection of invasive epithelial ovarian and/or tubal cancers (iEOCs) within 1 year of first screen (the prevalence screen) were 89.5%, 99.8%, and 35.1%, respectively.^9^

### MMS Strategy

**Fig 2.**
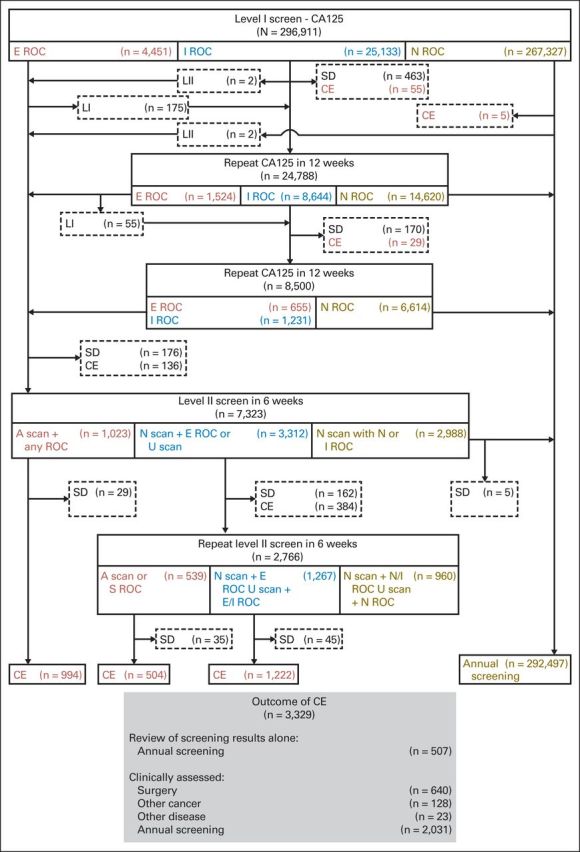
Multimodal screening algorithm and outcome of incidence screening. A, abnormal; CA-125, cancer antigen 125; CE, clinical evaluation; E, elevated; I, intermediate; LI, level I CA-125 test; LII, level II transvaginal scan (TVS) and CA-125 test; N, normal; ROC, risk of ovarian cancer; S, severe; SD, screening discontinued; U, unsatisfactory.

Following the initial prevalence screen, trial participants underwent an annual blood test on the anniversary of the randomization date. Serum CA-125 (level I screen) was measured by using an electrochemiluminescence sandwich immunoassay (Catalog No. 11776223 322; Roche Diagnostics, Mannheim, Germany).^10^ The screening protocol and management of screen-detected abnormalities have been previously described^8,9^ and are illustrated in Figure 2. In brief, at the annual screen, women were triaged as follows: risk of ovarian cancer (ROC) normal, return to annual screening; ROC intermediate, repeat CA-125 (repeat level I screen) in 12 weeks; and ROC elevated, repeat CA-125 and transvaginal scan (TVS; level II screen) in 6 weeks, with earlier screens arranged when results are suggestive of clinical disease. At level II screen, women with normal or intermediate ROC and a normal scan were returned to annual screening, whereas those with elevated ROC and a normal scan or an unsatisfactory scan irrespective of ROC had a repeat level II screen in 6 weeks. Those with abnormal scans irrespective of ROC were referred for clinical assessment. At repeat level II, women were again triaged to annual screening or clinical assessment (Fig 2). Women with an ROC of more than 1 in 5 (severe risk) were recommended to have surgery irrespective of scan findings.

The protocol was strictly enforced by using a custom-built Web-based trial management system with central classification of results, subsequent actions, and automated screening appointments.^9^ At study conception, the ROC cutoffs (< 1 in 1,818 and < 1 in 500) were set to allow approximately 15% and 2% of women to be triaged at annual screen to intermediate and elevated ROC groups, respectively. In April 2005, on the basis of data analysis on the performance of ROCA within UKCTOCS, the cutoffs were decreased to less than 1 in 3,500 and less than 1 in 1,000, respectively, to maintain the target proportions for triage.

### Clinical Assessment, Surgery, and Conservative Management

Clinical assessment and appropriate investigations were undertaken locally by a designated clinician. The latter included repeat CA-125, imaging (TVS with Doppler ultrasound, computed tomography, and/or magnetic resonance imaging of abdomen and pelvis) and other tumor markers. In women with severe ROC, a chest computed tomography scan and mammogram were also requested. All women who were thought to have cancer were discussed at the local gynecologic oncology multidisciplinary team meeting. If surgery was recommended, laparoscopic bilateral salpingo-oophorectomy was performed unless the assessment was definitively suggestive of ovarian cancer or the procedure was inappropriate for other reasons, in which case laparotomy was preferred. Women who underwent bilateral salpingo-oophorectomy and were found to have ovarian and/or tubal cancer had completion surgery with staging. In those who did not have surgery, the coordinating center was informed of the follow-up plan, which usually involved repeat CA-125/TVS every 3 months. When clinicians felt reassured that the woman was unlikely to have ovarian cancer, she was returned to annual screening within UKCTOCS.

All center staff were asked to report intra- and postoperative complications, return to operating theater, and readmissions by using standard UKCTOCS forms and to report any serious adverse events to a designated safety officer. In addition, coordinating center staff reviewed medical notes and follow-up questionnaire responses to capture any additional complications. All were independently reviewed by a senior trial gynecologic oncologist blinded to the randomization group.

### Confirmation of Diagnosis

In all women who underwent screen-positive surgery, copies of medical records including surgery notes, discharge letters, and histopathology and/or cytology reports were obtained as previously described.^9^ For women who resided in England, additional information up to March 31, 2010, was obtained from the Hospital Episode Statistics.^11^ In women diagnosed with cancer, further information was obtained, including the discharge summary, multidisciplinary team meeting notes, and other correspondence. These reports were also obtained for all women when there was notification through cancer registry, death certificate, follow-up questionnaire, or personal communication of a possible ovarian or tubal cancer (International Classification of Diseases and Related Health Problems [10th revision; ICD-10] codes; Appendix Table A1, online only). The case notes of all these individuals were reviewed using a strict protocol by an Outcomes Review Committee (two pathologists and two gynecologic oncologists) who were blinded to the randomization group. They confirmed the final diagnosis, stage, and morphology of any cancer and, when possible, they classified iEOCs into type I (low-grade serous, low-grade endometrioid, mucinous, and clear cell cancers) or type II (high-grade serous, high-grade endometrioid, carcinosarcomas, and undifferentiated carcinoma) cancers.^12^ Where it was not possible to delineate whether the primary site was ovary, fallopian tube, or peritoneum, the diagnosis was classified as undesignated.^13^

### Follow-Up

All volunteers were followed up through their National Health Service number by the appropriate national agencies for cancer registrations and/or deaths as well as by postal questionnaires 3 to 5 years after randomization and 2 years after the end of screening in the trial.^9^ The most recent cancer registrations for this analysis were received on June 17, 2014 (England and Wales), and July 2, 2014 (Northern Ireland).

### Analysis

A screen was defined as a single or series of serum CA-125 assays with or without scans culminating in surgery or return to annual screening. All women were censored at 1 year from last scan and/or CA-125 assay performed during their last incidence screen. The screen was considered positive (screen positive) if the woman had surgery or image-guided biopsy as a result of screening. Included in this category were women who were found to have ovarian lesions during imaging for other disease and who underwent surgery while awaiting repeat testing. The primary outcome for this analysis was primary iEOC diagnosed within 12 months of the last test in the incidence screen. Women with primary peritoneal cancer, borderline or nonepithelial ovarian cancers, and ovarian neoplasms of uncertain behavior were not included as true positives in the primary outcome analysis. A screen-detected cancer was one that resulted from screen-positive surgery and/or biopsy. A screen-negative/interval cancer was one diagnosed clinically within 12 months of the last test in the incidence screen in women returned to annual screening.

Overall sensitivity, specificity, PPV, and descriptive statistics for MMS were calculated for iEOCs and for all primary malignant ovarian and fallopian tube cancers (including borderline tumors and nonepithelial ovarian cancers).^9^ Receiver operating characteristic curves were constructed to compare the performance characteristics of annual serum CA-125 interpreted by using the ROCA with that of CA-125 interpreted by using several normal fixed cutoffs in this population, specifically, more than 35, more than 30, and more than 22 U/mL. A test for the difference in the area under the curves (AUCs) was performed as described by DeLong et al.^14^

## RESULTS

The CONSORT diagram (Fig 1) shows that 46,237 (91.3%) of the 50,640 women randomly assigned to the MMS arm participated in incidence screening. Between June 25, 2002, and December 21, 2011, 296,911 incidence screens were undertaken. Appendix Table A2 (online only) lists the reasons for screens that were not performed. The median number of incidence screens was seven (range, one to 10; interquartile range [IQR], six to eight). Median follow-up from the last incidence screen to latest cancer registration update was 3.1 years (IQR, 2.8 to 4.1 years).

Figure 2 and Appendix Table A3 (online only) summarize the results. In all, 10.0% (29,584 of 296,911 involving 20,485 of 46,237 volunteers) of annual screens resulted in a recommendation for a repeat screen. Use of a single-threshold rule for CA-125 of more than 35, more than 30, or more than 22 U/mL would have resulted in 1.9% (5,597 of 296,911 involving 2,253 of 46,237 volunteers), 3.3% (9,699 of 296,911 involving 3,537 of 46,237 volunteers), and 9.7% (28,757 of 296,911 involving 8,596 of 46,237 volunteers), respectively, of screens needing to be repeated.

Overall, 1,085 (0.4% of 296,911) of these screening episodes were not completed because women died (n = 95), changed their minds/moved away/did not attend repeat appointments (n = 689), were diagnosed with nonovarian cancer (n = 169) or other disease (n = 29), or had their ovaries removed as part of surgery for other conditions (n = 20).

Clinical evaluation was performed in 1.1% (3,329 of 296,911 involving 3,078 of 46,237 volunteers) of the screens (Fig 2). In 507 patients this was limited to assessment of screen results and return to annual screening. The remaining 2,822 screens resulted in clinical assessment; 3.6% (102 of 2,822) of the assessments were undertaken instead of protocol-mandated repeat testing. Reasons stated included CA-125 levels ≥ 50 U/mL (n = 50), elevated ROC (n = 68), or both (n = 38) and patient anxiety and/or symptoms suggestive of ovarian cancer (n = 22).

A proportion of the screens (0.2%; 640 of 296,911) resulted in women having screen-positive surgery, 64.8% (415 of 640) of which was laparoscopic. Primary ovarian and/or tubal malignancies were detected in 154 (24.1%) of the 640 women (Table 1). The latter included 133 iEOCs, 17 borderline, and four nonepithelial ovarian cancers. Two of the 154 women had incomplete screening episodes, and ovarian cancer (one iEOC, one nonepithelial microscopic granulosa tumor) was diagnosed in the course of imaging for renal disease and surgery for endometrial cancer, respectively, while awaiting repeat testing. Thirty-two interval ovarian or tubal cancers (22 iEOCs, nine borderline ovarian tumors, and one nonepithelial ovarian cancer) were diagnosed clinically within 12 months of the last incidence screen test. The 22 iEOCs include a protocol deviation in which the clinical team returned an asymptomatic woman estimated by ROCA to be at severe risk (one in four) to annual screening. Her CA-125 was 29 U/mL and pelvic magnetic resonance imaging was normal. Eight months later, she presented symptomatically with high-grade serous stage III iEOC (Table 1). In addition, a second woman was classified as intermediate risk by ROCA at both annual and first repeat screens but then classified as normal risk on her second repeat sample, at which point she was returned to annual screening. Eleven months later, she was diagnosed with stage IV high-grade serous cancer. An additional 21 iEOCs were diagnosed 12 to 24 months after the last annual screen.

**Table 1.** Pathologic Findings and CA-125 at Relevant Annual Screen (level I) in Screen-Positive Women and Screen-Negative Women (those with interval cancers)

Outcome of Screen-Positive Surgery	Total No. of Women	Annual CA-125	
		< 35 U/mL	≥ 35 U/mL
Total No. of women	640	455	185
Total No. of women with normal or benign pathology	441	344	97
Laparoscopy, ovaries normal, not removed*	13	12	1
Normal ovaries†	133	106	27
Benign ovarian pathology‡	295	226	69
Total No. of nonovarian malignant neoplasms	45	24	21
Ovarian neoplasm of uncertain behavior (ICD D39.1)	2	2	0
Primary peritoneal cancer (ICD C48.2)	12	6	6
Other nonovarian and/or tubal cancer involving ovaries (secondary ovarian neoplasm)*	12	6	6
Other nonovarian and/or tubal cancer not involving ovaries§	19	10	9
Total No. of screen-positive women diagnosed with malignant neoplasm of ovary (ICD C56) and fallopian tube (ICD C57.0)	154	87	67
Nonepithelial neoplasm of ovary (ICD C56)	4	3	1
Primary borderline epithelial neoplasm of ovary (ICD C56)	17	14	3
Primary invasive epithelial neoplasm of ovary (ICD C56)	113	56	57
Primary invasive epithelial neoplasm of fallopian tube (ICD C57.0)	11	8	3
Undesignated (unable to delineate whether primary site is ovary, fallopian tube, or peritoneum)	9	6	3
Total No. of women with screen-negative (interval) malignant neoplasm of ovary (ICD C56) or fallopian tube (ICD C57.0) diagnosed within 1 year of end of screen	32	31	1
Nonepithelial neoplasm of ovary (ICD C56)	1	1	0
Borderline epithelial neoplasm of ovary (ICD C56)	9	9	0
Primary invasive epithelial neoplasm of ovary (ICD C56)	18	17	1
Primary invasive epithelial neoplasm of fallopian tube (ICD C57.0)	1	1	0
Undesignated (unable to delineate whether the primary site is ovary, fallopian tube, or peritoneum)	3	3	0

Abbreviations: CA-125, cancer antigen 125; ICD, International Statistical Classification of Diseases and Related Health Problems (10th revision).

* Includes a volunteer who had ultrasound-guided aspiration of ascites in her year 4 screen with normal cytology and was returned to annual screening. In her next screen, she had screen-positive laparotomy with a final diagnosis of colorectal primary metastatic to the ovaries.

† Includes five women with para-tubal cysts, three with benign hydrosalpinx, one with mucinous cystadenoma of the appendix, and one with tumor-bearing endometrium.

‡ Includes one volunteer who had benign ovarian cysts at surgery. However, CA-125 continued to increase, and 1 year later, she was diagnosed with primary peritoneal cancer.

§ Includes six women who also had benign ovarian pathology.

**Fig 3.**
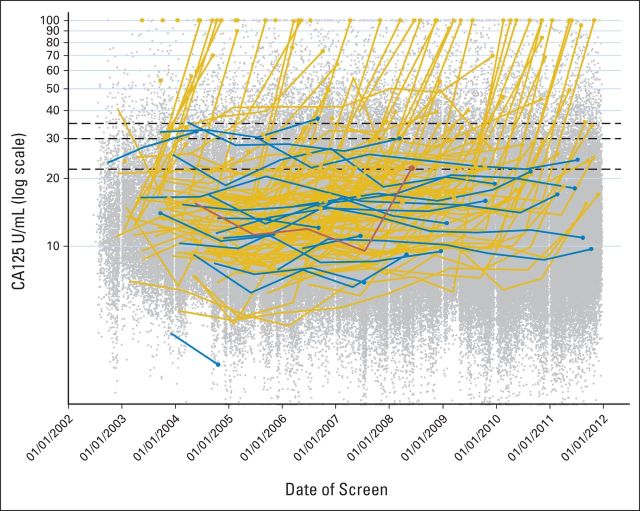
Plot of all multimodal screening annual cancer antigen 125 (CA-125) measurements over time on a log scale, including truncation. Superimposed are the serial measurements for 155 invasive epithelial ovarian and/or tubal cancers with the large circles representing the final screen before diagnosis, either true positive (n = 133; gold lines and markers) or false negative (n = 22; blue lines and markers). The red line indicates one patient in whom the risk of ovarian cancer algorithm recommended surgery, but it was not performed following clinical evaluation. The black horizontal lines represent CA-125 cutoffs of 35, 30, and 22 U/mL. NOTE. 262 CA-125 values truncated above 100 U/mL and 174 CA-125 values truncated below 2 U/mL.

**Fig 4.**
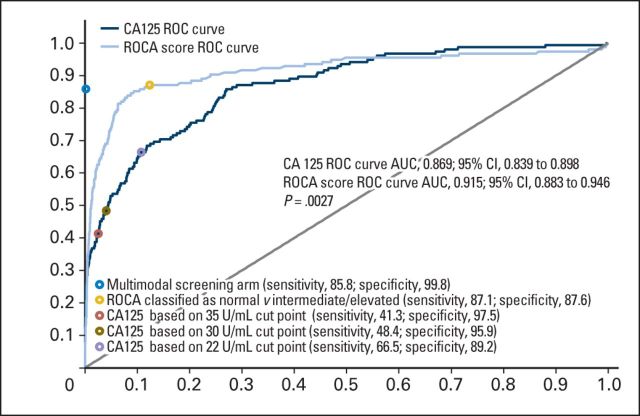
Risk of ovarian cancer (ROC) curves based on the performance characteristics of annual cancer antigen 125 (CA125) measurement alone and annual risk of ovarian cancer algorithm (ROCA) score alone. Overlaid points represent the actual characteristics of the multimodal screening strategy, hypothetical characteristics of annual ROCA classified as normal or abnormal (intermediate/elevated) risk, hypothetical characteristics of annual CA125 using the cutoff points of more than 35 U/mL (as in the Prostate, Lung, Colorectal and Ovarian Cancer Screening Trial), more than 30 U/mL (in clinical use), and more than 22 U/mL (as suggested by other groups), respectively. *P* value of .0027 is test of difference.

At the relevant annual screen, median serum CA-125 in the 133 women with screen-detected iEOCs was 33.6 U/mL (IQR, 21.3 to 109.2). Seventy (52.6%) of 133 of these women had CA-125 levels within the normal range (≤ 35 U/mL; subgroup A), and the remaining 63 (47.4%) had increased CA-125 levels (> 35 U/mL; subgroup B; Table 2). Only one of the 22 women who had an interval iEOC had a CA-125 level more than 35 U/mL (36.9 U/mL). These results are shown graphically in Figure 3, in which the serial annual CA-125 levels of all screen-positive (n = 133) and screen-negative patients with iEOC (n = 22) are plotted with the annual CA-125 levels for all other women shown as a scatterplot. The ROCA had a significantly larger area under the curve (0.915) than the individual CA-125 measurements (0.869; *P* = .0027; Fig 4). The sensitivity of ROCA alone was 87.1% (95% CI, 80.8% to 91.9%) and that of using annual serum CA-125 cutoffs of more than 35, more than 30, and more than 22 U/mL were 41.3%, (95% CI, 33.5% to 49.5%), 48.4% (95% CI, 40.3% to 56.5%), and 66.5% (95% CI, 58.4% to 73.8%), respectively. The specificity of annual ROCA alone was 87.6%. At the same specificity, the sensitivity of the annual CA-125 cutoff (20.99 U/mL) was 68.4%.

**Table 2.** CA-125 at the Relevant Annual Screen by Stage and Type of Primary Invasive Epithelial Ovarian and Tubal Cancers

Characteristic	Screen-Detected Status											
	Positive									Negative		
	All			Annual CA-125 < 35 U/mL (subgroup A)			Annual CA-125 ≥ 35 U/mL (subgroup B)			All		
	No.	%	95% CI	No.	%	95% CI	No.	%	95% CI	No.	%	95% CI
Total No. of women	133			70			63			22		
Serum CA-125 at corresponding annual screen, U/mL												
Median	33.6			21.8			112.1			13.6		
IQR	21.3-109.2			16.5-26.3			66.4-375.4			11-20.8		
ROC at corresponding annual screen												
Normal risk	0	0		0	0		0	0		20	90.9	
Intermediate risk	37	27.8		33	47.1		4	6.3		1	4.5	
Elevated risk	96	72.2		38	54.3		58	92.1		1	4.5	
Stage												
I	35			22			13			4		
II	20			12			8			2		
III	68			32			36			11		
IIIa	6			2			4			0		
IIIb	16			11			5			3		
IIIc	46			19			27			8		
IV	10			4			6			5		
Early stage (I or II)		41.4	32.9 to 50.2		48.6	36.4 to 60.8		33.3	22.0 to 46.3		27.3	10.7 to 50.2
Morphology												
Total type I iEOC	19			10			9			5		
Low-grade serous	5			1			4			0		
Low-grade endometrioid	8			4			4			1		
Clear cell	5			4			1			4		
Mucinous	1			1			0			0		
Total type II iEOC	109			58			51			11		
High-grade serous*	89			44			45			8		
High-grade endometrioid	8			5			3			1		
Unspecified adenocarcinoma	10			8			2			1		
Carcinosarcoma (MMT)	2			1			1			1		
Unclassified†	5			2			3			6		

Abbreviations: CA-125, cancer antigen 125; iEOC, invasive epithelial ovarian and/or tubal cancer; IQR, interquartile range; MMT, malignant mesenchymal tumor; ROC, risk of ovarian cancer.

* Includes a case reported as mixed high-grade adenocarcinoma with serous and clear cell features and focal squamous differentiation.

† Morphology could not be determined because only cytology was undertaken.

Of the screen-detected iEOCs, 82.0% (109 of 133) were type II. The distributions of type I and type II cancers in the A and B subgroups were similar (Table 2). Fifty-five (41.4%) of 133 patients with iEOCs were diagnosed in stage I to II (Table 2). A greater proportion (*P* = .075) in subgroup A (48.6%; 34 of 70) were early-stage (stage I to II) cancers compared with subgroup B (33.3%; 21 of 63).

Overall, in women with screen-detected iEOCs, the median time from last screen test to surgery was 8 weeks (IQR, 4.9 to 13.7 weeks), and the median time from the start of the relevant annual screen (level I) to surgery was 20 weeks (IQR, 11 to 34 weeks). In subgroup A, the interval was significantly (*P* < .0001) longer (30 weeks; IQR, 18 to 43 weeks) compared with subgroup B (12 weeks; IQR, 7 to 19 weeks). This difference reflects the greater proportion of cases undergoing repeat screens following an intermediate ROC at annual screen in subgroup A (33 of 70) compared with subgroup B (four of 63; Table 2).

The overall sensitivity and specificity of MMS for iEOCs were 85.8% (95% CI, 79.3% to 90.9%) and 99.8% (95% CI, 99.8% to 99.8%), respectively, with 4.8 surgeries per iEOC detected during incidence screening (Table 3). If the 12 screen-detected and three screen-negative primary peritoneal cancers (PPCs) were included, sensitivity, specificity, and PPV were 85.3% (95% CI, 79.1% to 90.3%), 99.8% (95% CI, 99.8% to 99.8%), and 22.7% (95% CI, 19.5% to 26.1%), respectively. If we extended performance characteristics to include iEOCs diagnosed up to 24 months from date of last scan/CA-125 assay performed during incidence screening, sensitivity for iEOCs was 74.4%.

**Table 3.** Performance Characteristics of MMS Incidence Screening for Malignant Ovarian (C56), Tubal (C57.0), and Primary Peritoneal (C48.2) Neoplasm

Characteristic	Ovarian and Fallopian Tube Cancers		Ovarian, Fallopian Tube, and Primary Peritoneal Cancers	
	No.	95% CI	No.	95% CI
No. of women-years	296,911		296,911	
No. of surgeries	640		640	
Primary ovarian (C56) and tubal (C57.0) malignancies and primary peritoneal cancer (C48.2) within 1 year of screen (includes borderline and ovarian neoplasm of uncertain behavior)				
Screen positive	154		166	
Screen negative	32		35	
Sensitivity	82.8	76.6 to 87.9	82.6	76.6 to 87.6
Specificity	99.8	99.8 to 99.9	99.8	99.8 to 99.9
PPV	24.1	20.8 to 27.6	25.9	22.6 to 29.5
No. of operations per screen positive	4.2		3.9	
Primary invasive epithelial ovarian, tubal, and primary peritoneal malignancies within 1 year of screen (excludes borderline epithelial ovarian neoplasms)				
Screen positive	133		145	
Screen negative	22		25	
Sensitivity	85.8	79.3 to 90.9	85.3	79.1 to 90.3
Specificity	99.8	99.8 to 99.8	99.8	99.8 to 99.8
PPV	20.8	17.7 to 24.1	22.7	19.5 to 26.1
No. of operations per screen positive	4.8		4.4	

NOTE. All codes are International Statistical Classification of Diseases and Related Health Problems, 10th revision (ICD-10).

Abbreviations: MMS, multimodal strategy; PPV, positive predictive value.

Of the 640 women who had screen-positive surgery, 31 had nonovarian cancers, and 441 had normal or benign pathology (Table 1; Appendix Table A4, online only). An intraoperative or early postoperative complication was reported in 20 of the 441 women (4.5%; 95% CI, 2.8% to 6.9%). Twelve of these women had a major complication or significant sequelae (Appendix Table A5, online only).

## DISCUSSION

In the largest ovarian cancer screening trial that we are aware of, a risk algorithm using serial biomarker measurement doubled the number of screen-detected cancers compared with a single-threshold rule. Of the 155 women with iEOCs, the ROCA detected 86.4% whereas using annual serum CA-125 fixed cutoffs of more than 35, more than 30, and more than 22 U/mL would have identified only 41.3%, 48.4%, and 66.5%, respectively. Our data provide prospective evidence of the improvement that CA-125 velocity analysis brings to iEOC detection compared with a predetermined cutoff. The impact of such screening on ovarian cancer mortality will be known later in 2015 when follow-up is complete. However, our current findings are of immediate importance because they highlight the need to examine serial change in biomarker levels in the context of screening and early detection of cancer. Reliance on predefined single-threshold rules may result in biomarkers of value being discarded.

The encouraging sensitivity (85.8%) and specificity (99.8%) for detecting iEOCs in low-risk postmenopausal women noted during the prevalence screen persisted during incidence screening.^9^ The high sensitivity remained even when PPC was included as an outcome measure. This was reassuring given that PPC probably shares common origins with primary high-grade serous iEOCs.^15^ The ROCA increases sensitivity by personalizing the interpretation of serial biomarker values. This explains the higher sensitivity observed in our trial compared with other trials in which a single-threshold CA-125 rule was used—67% in the PLCO trial^16^ (four rounds of screening including prevalence) and 77% in the Shizuoka Cohort Study.^17^

Overall, 41.4% (55 of 133) of women were detected with stage I or II disease. A majority (82.0%) of screen-detected iEOCs were aggressive type II, which are associated with the highest mortality rates.^18^ This is reassuring, given the concern that screening detects more indolent cancers. In the Shizuoka Cohort Study, 48% of screen-detected cancers were type I mucinous and clear cell iEOCs.^17^

The strategy involved at least one repeat test such that the median time from annual screen to surgery was 20 weeks. The interval was significantly longer in subgroup A (30 weeks) compared with subgroup B (12 weeks) because women with annual CA-125 levels in the normal range required more repeat testing. Despite this, there was a higher proportion of stage I or II iEOCs in subgroup A. The latter coupled with the fact that ovarian cancers double every two and half months,^19^ suggests that modifications to the screening strategy that could decrease this interval may have an additional impact on tumor stage and volume. This could include decreasing the 3-month interval to repeat CA-125 testing following an intermediate ROC and measuring levels of a second blood biomarker such as HE4^20,21^ in intermediate-risk annual samples. Although HE4 does not improve CA-125 lead time,^22,23^ it could help confirm ovarian cancer risk and reduce time to surgery. In the presence of an increasing CA-125, HE4 was increased in samples from 27 of 39 women with ovarian cancer in the PLCO trial.^21^ TVS does not seem to have the resolution to detect iEOC at low CA-125 levels. Twenty-nine (41%) of 70 women with iEOCs in subgroup A had no abnormality on the initial level II scan, and TVS was abnormal in only 17 of the 39 women in the study by Urban et al.^21^ The potential of newer technology such as contrast-enhanced TVS with targeted microbubbles warrants assessment in this context.^24^

For each iEOC detected, four additional women underwent surgery. These figures are slightly higher than previously reported in trials using the ROCA^9,25,26^ but lower than the 19.5^16^ and 33^17^ surgeries undertaken for each cancer detected in trials using other screening strategies. Excess surgical morbidity in patients with false-positive diagnoses is a key concern, especially with increasing comorbidity in the older women. In our study, the rate of complications in women with benign or normal histology, most of whom underwent laparoscopic bilateral salpingo-oophorectomy, was 4.5%. Similar rates have been reported in women at high-risk of ovarian cancer undergoing risk-reducing salpingo-oophorectomy (3.9%).^27^

Key strengths of our trial are the scale, the multicenter setting within the United Kingdom health service, detailed screening and management protocols implemented by a dedicated local and central team, Web-based bespoke trial management system, high compliance with screening, and independent blinded outcome review. Completeness of data on screen-negative and/or interval cancers in the year following the end of screening (2012) was ensured by postal follow-up of all women in April 2014, coupled with cancer registry updates in July 2014. The limitations relate mainly to the long duration, a necessary feature of randomized controlled trials with mortality as the primary end point, and the associated improvements in clinical management over that period. A healthy volunteer effect reduced the expected number of cancers in the control arm and thereby further lengthened the trial.^28^ However, although these issues are pertinent to this analysis, they will not affect the primary intention-to-treat mortality analysis.

In conclusion, our data support use of velocity-based algorithms as opposed to a predefined single-threshold rule in cancer screening strategies that use blood biomarkers.

## Supplementary Material

Protocol

Publisher's Note

## Glossary Terms

biomarker: a functional biochemical or molecular indicator of a biologic or disease process that has predictive, diagnostic, and/or prognostic utility.
CA-125 (cancer antigen 125): a protein produced by the fallopian tubes, the endometrium, and the lining of the abdominal cavity (peritoneum). CA-125 is a tumor marker present in higher than normal amounts in the blood and urine of patients with certain cancers. Typically, women with ovarian cancer have high levels of CA-125. Other conditions associated with increased levels of CA-125 include endometriosis, pancreatitis, pregnancy, normal menstruation, and pelvic inflammatory disease. CA-125 levels may be used to help diagnose ovarian cancer and to determine whether these tumors are responding to therapy. The normal range for CA-125 is less than 35 U/mL and less than 20 U/mL for women who have been treated for ovarian cancer. Women with ovarian cancer may show values higher than 65 U/mL.
positive predictive value: the probability of a positive test result being truly positive.

## Appendix

**Table A1.** ICD-10 Codes for Notes Reviewed by the Outcomes Committee

ICD-10 Code	Description
C56	Malignant neoplasm of ovary
C57.0	Malignant neoplasm of fallopian tube
C57.4	Uterine adnexa, unspecified
C57.7	Other specified female genital organs
C57.8	Malignant neoplasm of overlapping lesion of female genital organs
C57.9	Malignant neoplasm of female genital organ, unspecified
C48.0	Retroperitoneum
C48.1	Specified parts of peritoneum
C48.2	Malignant neoplasm of peritoneum, unspecified
C48.8	Overlapping lesions of retroperitoneum and peritoneum
C76.2	Malignant neoplasm of abdomen
C76.3	Malignant neoplasm of pelvis
C80	Malignant neoplasm without specification of site
D07.3	Carcinoma-in-situ of other or unspecified female genital organ
D28.2	Benign neoplasm of fallopian tube
D28.9	Benign neoplasm of female genital organ, unspecified
D36.9	Benign neoplasm of unspecified site
D39.1	Neoplasm of uncertain or unknown behavior of ovary
D39.9	Neoplasm of uncertain or unknown behavior of female genital organ, unspecified

Abbreviation: ICD-10, International Statistical Classification of Diseases and Related Health Problems (10th revision).

**Table A2.** Reasons Why Screens That Should Have Been Performed Were Not Undertaken

Reason Screen Was Not Performed	No. of Screens	No. of Women
Died	6,945	1,781
Decided to discontinue	73,632	16,393
Ovaries removed	4,505	1,060
Cancer diagnosed	3,425	953
Over-ran previous screen	3,105	2,874

**Table A3.** Results of Incidence Screens

Incidence Screens	Woman-Years	
	No.	%
Level 1 screen*	296,911	100
Normal ROC, returned to annual screening	267,327	90.0
Intermediate ROC, referred for repeat level 1 screen	25,133	8.5
Elevated ROC, referred for level 2 screen	4,451	1.5
Repeat level 1 CA-125†	24,788	8.3
Returned to annual screening	21,234	85.7
Referred for level 2 screen	3,355	13.5
Did not complete all repeat screens	199	0.8
Level 2 screen†	7,323	2.5
Returned to annual screening	2,988	40.8
Referred for clinical assessment	1,023	14.0
Referred for repeat level 2 screen	3,312	45.2
Repeat level 2 screen†	2,766	0.9
Returned to annual screening	960	34.7
Referred for clinical assessment	1,806	65.3
Clinical assessments‡	3,329	1.1
Screen-positive surgery	640	0.2
Diagnostic laparoscopy	17	2.7
Operative laparoscopy	356	55.6
Combined laparoscopy and laparotomy	42	6.6
Laparotomy	206	32.2
Imaging-guided cytology and/or biopsy	15	2.3
Other	4	0.8

Abbreviations: CA-125, cancer antigen 125; ROC, risk of ovarian cancer.

* Denominators for header rows are number of incidence screens. Denominators for subsequent rows are numbers of women who underwent a specific screen.

† Difference in numbers between those who were recommended tests and number who underwent test is because of noncompliance.

‡ In all, 109 (29 + 35 + 45) women withdrew before a clinical assessment was performed and 609 (55 + 5 + 29 + 136 + 384) additional women were clinically evaluated before completing all protocol screens.

**Table A4.** Screen-Detected Nonovarian, Tubal, or Primary Peritoneal Cancer

Cancer Type	No. of Women
Women with other nonovarian or tubal cancers not involving the ovaries (n = 19)	
Appendiceal	2
Endometrial	8
Lymphoma	3
Malignant neoplasm of unknown but not ovarian or tubal origin	1
Breast	1
Colorectal	1
Pancreatic	1
Liver	1
Renal	1
Women with other nonovarian or tubal cancers involving the ovaries (secondary ovarian neoplasm; n = 12)	
Appendiceal	1
Breast	3
Colorectal	3
GI	2
Lymphoma	1
Endometrial	2

**Table A5.** Details of the Complications in Women Who Had Normal Ovaries or Benign Pathology at Screen-Positive Surgery

Intra- and Early Postoperative Complications	Women	
	No.	%
Major		
Intraoperative episode of severe tachycardia with asystole requiring cardiopulmonary resuscitation	1	
Bowel obstruction*	4	
Bowel injury	2	
Hemorrhage†	3	
Wound dehiscence requiring resuturing	1	
Significant ileus	1	
Minor		
Wound infection requiring antibiotics	5	
Chest infection	1	
Diarrhea and vomiting	1	
Perforation of uterus, urinary retention, and UTI	1	
Total number of benign surgeries with complications	20	
Total number of benign surgeries	441	
Complication rate		4.5

Abbreviation: UTI, urinary tract infection.

* One small bowel obstruction from port site hernia, one subacute bowel obstruction requiring readmission.

† One from rectus sheet bleed, one from umbilical port site hematoma, one two-unit transfusion.
