# Supplementary material for: Risk Algorithm Using Serial Biomarker Measurements Doubles the Number of Screen-Detected Cancers Compared With a Single-Threshold Rule in the United Kingdom Collaborative Trial of Ovarian Cancer Screening
Source: J Clin Oncol. 2015 May 11;33(18):2062–71. doi: 10.1200/JCO.2014.59.4945 (PMC4463475; doi:10.1200/JCO.2014.59.4945)
Supplement: Protocol [file supp_JCO.2014.59.4945_Protocol_JCO.2014.59.4945.pdf]

The following protocol information is provided solely to describe how the authors conducted the research underlying the published report associated with the following article:

A risk algorithm using serial biomarker measurements doubles the number of screen-detected cancers compared to a single threshold rule in the United Kingdom Collaborative Trial of Ovarian Cancer Screening (UKCTOCS)

Menon, et al

DOI: 10.1200/JCO.2014.59.4945

The information provided may not reflect the complete protocol or any previous amendments or modifications. As described in the Information for Contributors (http://jco.ascopubs.org/site/ifc/protocol.xhtml) only specific elements of the most recent version of the protocol are requested by JCO. The protocol information is not intended to replace good clinical judgment in selecting appropriate therapy and in determining drug doses, schedules, and dose modifications. The treating physician or other health care provider is responsible for determining the best treatment for the patient. ASCO and JCO assume no responsibility for any injury or damage to persons or property arising out of the use of these protocol materials or due to any errors or omissions. Individuals seeking additional information about the protocol are encouraged to consult with the corresponding author directly.

Protocol for the United Kingdom Collaborative Trial of Ovarian Cancer Screening (UKCTOCS)

Version: 7.1

Date: April 2015

Trial Title:

United Kingdom Collaborative Trial of Ovarian Cancer Screening (UKCTOCS)

International Standard Randomised Controlled Trial Number: 22488978

Trial sponsor: University College London

Summary

In the UK, ovarian cancer (OC) causes more deaths than all the other gynaecological cancers put together. Majority of patients present with advanced disease which is associated with poor survival. In contrast, five-year survival rates for women diagnosed with stage I OC are over 90%, suggesting that early detection through screening may impact on outcome. Preliminary data from screening trials supports this view. The UK Collaborative Trial of Ovarian Cancer Screening (UKCTOCS) is a multicentre randomised control trial which aims to assess the impact of screening on ovarian cancer mortality while comprehensively evaluating physical and psychological morbidity, compliance and resource implications of screening and performance characteristics of a serum CA125 versus ultrasound based screening strategy. Women aged 50 to 74 were randomly invited from age/sex registers of Primary Care Trusts adjoining 13 trial trial centres in England, Wales and Northern Ireland. Those who accepted the invitation were sent appointments to attend recruitment clinics. After confirmation of eligibility, 202,638 postmenopausal women were randomised between 2001 and 2005 to screening with transvaginal ultrasound (U/ultrasound group), serum CA125 interpreted using a ‘Risk of Ovarian Cancer’ algorithm (M/multimodal group) or control (C group) in a 1:1:2 ratio. Women in the U and M groups receive annual screening till the end of 2011 using well-defined strategies which incorporate repeat testing. If a persistent suspicious abnormality is noted, they undergo clinical assessment and surgery. All women are followed to the end of 2024 via a ‘flagging study’ through the NHS Information Centre for Health and Social Care (formerly Office of National Statistics) and postal questionnaires. The sample size allows over 80% power at the (two-sided) 5% significance level to detect a reduction in ovarian cancer mortality of 30% in C versus M and U groups alone.

Background

In the UK, ovarian cancer (OC) causes more deaths than all the other gynaecological cancers put together. In 2004 in England and Wales, it accounted for 6% of all female cancer deaths with 5,293 women dying of the disease.[1] Whilst advances in therapy have improved median survival during the last decade, there has been little or no change in the overall mortality rate.[1,2]

Stage is one of the most important determinants of survival with most patients presenting with advanced disease, possibly as a result of non specific symptoms and delays in diagnosis. [3] The small proportion of patients who are diagnosed with stage I OC have five-year survival rates in excess of 90%.[4] This suggests that early detection through screening may impact on outcome.

During the 1990s, large prospective studies of screening have demonstrated that both CA125 and ultrasound can detect OC in asymptomatic women. However, the impact on mortality of preclinical detection of OC by screening remains uncertain. Preliminary data supports optimism. In a randomised controlled trial of ovarian cancer screening (OCS), median survival in women with OC in the screened group (72.9 months) was significantly better when compared to the control group (41.8 months).[5] Data from single arm prospective studies also suggest a possible survival benefit [6,7] although in these cases, lack a control group could lead to significant bias from a "healthy-volunteer effect".

Screening is not without morbidity. A proportion of women will undergo unnecessary surgery following false positive results and a few of these surgical procedures will lead to complications and rarely death. Secondly, there may be psychological sequelae resulting from screening, repeat testing and receiving false positive/negative results. Thirdly, there are major cost implications of a population screening programme. Resource implications, psychological sequelae and physical morbidity will be key factors in assessing the overall impact of OC screening.

In 1998, the NHS Centre for Reviews and Dissemination completed a systematic review which found that screening with ultrasound and serum CA125 can detect ovarian cancer in asymptomatic women but that the impact of screening on outcome is unproven. The recommendation was that screening should not be introduced into clinical practice until further information was available from randomised trials designed to document the impact of screening on OC mortality as well as the adverse effects and the cost effectiveness of screening.[8] Based on this, the UK Exceptional Cases Advisory Committee concluded that a well designed trial of OCS would be of value to the National Health Service. Lay groups like OVACOME and the National Federation of Women's Institutes urged the UK government to support research into OCS. In parallel, it was clear that there was only a limited window of time to perform a randomised controlled trial (RCT) on the effectiveness of OCS before media publicity and demand from individuals, lay groups and sectors of the medical profession led to widespread implementation. The UK Collaborative Trial of Ovarian Cancer Screening (UKCTOCS) was set up in response to these needs and commenced recruitment in 2001.

Principal investigators

Professor Ian Jacobs (Chief Investigator), Honorary Professor of gynaecological oncology and consultant gynaecological oncologist, Department of Women's Cancer, UCL EGA Institute for Women's Health, London W1T 7DN and President/Vice Chancellor, UNSW Australia, High Street Kensington, Sydney NSW 2032, Australia. Professor Usha Menon (Trial Coordinator), Professor of gynaecological oncology, Head of Gynaecological Cancer Research Centre and consultant gynaecologist, Department of Women's Cancer, UCL EGA Institute for Women's Health, London W1T 7DN

Co-investigators

Professor Mahesh Parmar (Trial Statistician), Professor of medical statistics and epidemiology, and Director of the MRC Clinical Trials Unit and the Institute of Clinical Trials and Methodology at University College London., Aviation House, London, WC2B 6NH.

Dr Steve Skates Associate Professor of Biostatistics, Department of Medicine, Harvard Medical School, Boston, MA, US MA 02115

Professor Stuart Campbell Professor and consultant obstetrician and gynaecologist, Create Health Clinic, London W1G 6AJ

Professor Lesley Fallowfield (Chief Investigator – Psychosocial study), Professor of psycho-oncology, Sussex Health Outcomes Research & Education in Cancer (SHORE-C), University of Sussex, Sussex, UK, BN1 9PX

Professor Ali McGuire, Professor of economics, London School of Economics, London WC2A 2AE

Study centres

Coordinating Centres

Main trial: The trial is coordinated by the Gynaecological Cancer Research Centre, UCL EGA Institute for Women's Health, University College London, 149 Tottenham Court Road, London W1T 7DN. In the early part of the trial (2001-2004), the coordinating centre group was located in the Department of Gynaecological Oncology, at Queen Mary University London.

The CC team includes a senior clinician (trial coordinator), a data manager, a senior research nurse, a laboratory manager, a statistician, two postdoctoral research fellows, a clinical fellow and a team of administrative and laboratory staff. It is actively involved in the day-to-day running of the trial and the implementation of the protocol through a custom-built trial management system (TMS).

Psychosocial study: This is coordinated by the Sussex Health Outcomes Research & Education in Cancer (SHORE-C), University of Sussex, Sussex, UK, BN1 9PX;

Trial Centres

The trial is conducted through 13 trial centres (TC) in England, Wales and Northern Ireland which are mainly located in NHS Trusts. Each centre has a dedicated trial team consisting of a research nurse, phlebotomist, clerk and ultrasonographers led by a consultant clinician (Lead Researcher). The trial centres undertake recruitment of the participants and performance of the screening tests. The teams arrange clinical evaluation of women confirmed to have abnormalities on screening and referral to the appropriate NHS clinic if surgery is appropriate.

The centres are

(1) Northern Gynaecological Oncology Centre, Queen Elizabeth Hospital, Gateshead NE9 6SX;

(2) Department of Gynaecological Oncology, St. Bartholomew's Hospital, London EC1A 7BE;

(3) Department of Gynaecology, Liverpool Women's Hospital, Liverpool L8 7SS;

(4) Department of Gynaecological Oncology, Nottingham City Hospital, Nottingham NG5 1PB ;

(5) Academic Unit of Obstetrics and Gynaecology, St. Mary's Hospital, Manchester M13 9WL;

(6) Department of Gynaecological Oncology, Derby City Hospital, Derby DE22 3NE;

(7) Department of Gynaecological Oncology, Royal Free Hospital, London NW3 2QG;

(8) Department of Gynaecological Oncology, St. Mary's Hospital, Portsmouth PO3 6AD;

(9) Department of Gynaecological Oncology, St. Michael's Hospital, Bristol BS2 8EG;

(10) Department of Gynaecological Oncology, Belfast City Hospital, Belfast BT9 7AB;

(11) Department of Obstetrics and Gynaecology, University of Wales College of Medicine, Cardiff CF14 4XN;

(12) Department of Gynaecological Oncology, Llandudno Hospital, Llandudno, North Wales LL30 1LB;

(13) Department of Gynaecological Oncology, James Cook University Hospital, Middlesbrough TS4 3BW;

Contact details

Prof Usha Menon

Gynaecological Cancer Research Unit

UCL EGA Institute for Women’s Health, London W1T 7DN, UK

Tel: +44 (0)20 3 447 2108

Fax: +44 (0)20 3 447 2129

Email: u.menon@ucl.ac.uk

Aims of the trial

The overall aim of the UK Collaborative Trial of Ovarian Cancer Screening (UKCTOCS) is to provide data so that an informed decision about the introduction of population screening for ovarian cancer can be made.

Primary objective

To establish the impact of screening on ovarian cancer mortality by comparing disease mortality in the screen and control groups.

Secondary objectives

(1) To assess and compare the performance characteristics of the two screening strategies (serum CA125 versus ultrasound)

(2) To determine the physical morbidity resulting from surgical intervention attributable to screening

(3) To assess the psychological consequences of screening

(4) To determine the resource implications of screening and the resulting interventions

(5) To assess feasibility of screening, as reflected by compliance rates with annual screening

(6) To establish a serum bank for future assessment of novel tumour markers

Design

UKCTOCS is a RCT of postmenopausal women aged 50 to 74 years randomised to (1) Study group: There is a 1:1 randomisation within this group to (a) An ultrasound group (U, annual screening with transvaginal ultrasound) and (b) A multimodal group (M, annual screening with serum CA125 as a primary test and ultrasound as a secondary test). (Figure 1) (2) Control group: No screening

Figure 1: Trial design

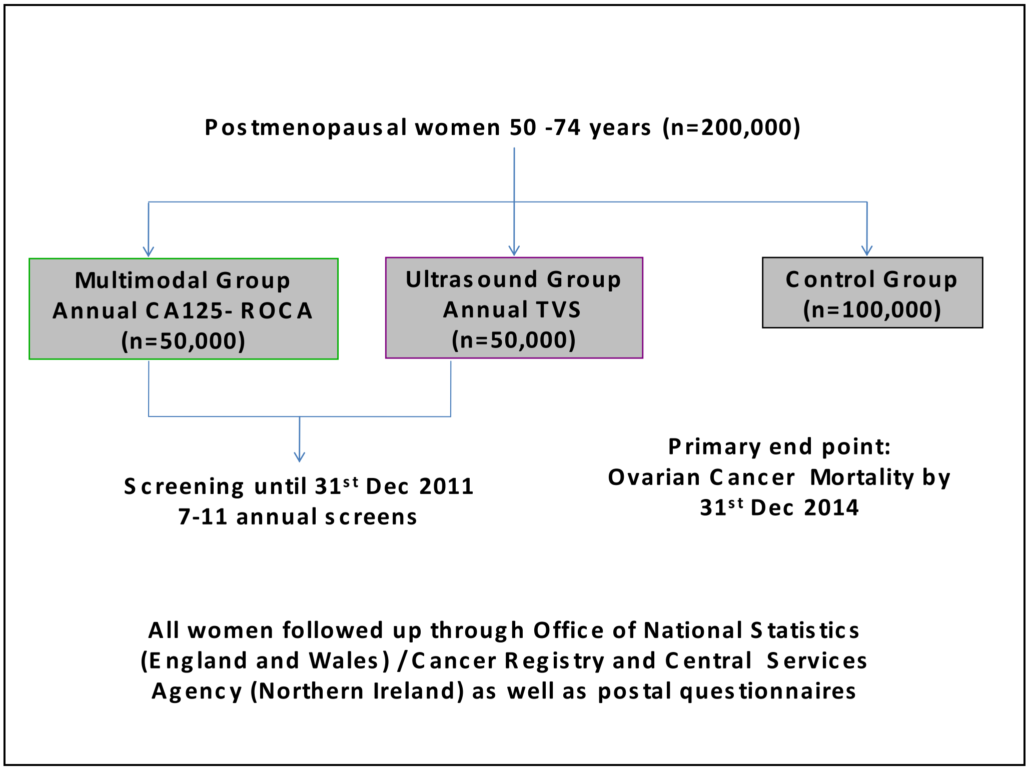

<u>Inclusion criteria</u>

(1) Age 50-74 years

(2) Postmenopausal: This is defined as either (a) >12 months amenorrhoea following a natural menopause or hysterectomy, or (b) >12 months of hormone replacement therapy commenced for menopausal symptoms. The restriction is because premenopausal women have a greater incidence of benign and physiological conditions associated with false positive findings on multimodal and ultrasound screening. The criteria does not entirely exclude premenopausal women, but it is not feasible to determine FSH levels in all

volunteers and such an approach would not be applicable to women taking hormone replacement therapy.

<u>Exclusion criteria</u>

(1) Previous ovarian malignancy;

(2) History of bilateral oophorectomy;

(3) Active non-ovarian malignancy: Women who have a past history of malignancy are only eligible if (a) they have no documented persistent or recurrent disease and (b) have not received treatment for >12 months. The intention is to minimise false positive CA125 results due to advanced stages of previously diagnosed malignancy. This exclusion does not include premalignant disease such as cervical intraepithelial neoplasia or use of Tamoxifen to prevent breast cancer recurrence.

(4) Increased risk of familial ovarian cancer: The high-risk women are eligible for a separate trial, the United Kingdom Familial Ovarian Cancer Screening Study (UKFOCSS). They are defined by the eligibility criteria for UKFOCSS. These women are first-degree (1°) relatives (mother, sister, daughter) of a cancer affected member of a “high risk” family. The high-risk family is defined by one of the following criteria:

- Two or more individuals with OC who were 1° relatives
- One individual with OC and 1 individual with breast cancer diagnosed under 50 years who are 1° relatives
- One individual with OC and 2 individuals with breast cancer diagnosed under 60 years who are connected by 1° relationships

- An affected individual with a mutation of one of the known ovarian cancer predisposing genes (BRCA1, BRCA2, MSH1)
- One person with OC and three individuals with colorectal cancer with at least one case diagnosed before 50 years, all connected by 1° relationships.
- Affected relatives fulfilling criteria 1, 2 or 3 who are related by second degree through an unaffected intervening male relative

(5) Participation in other ovarian cancer screening trials: It should be noted that after the start of UKCTOCS, there were no other OCS trials in the general population in the UK.

Ethical issues

The trial involves 13 Trial Centres (RC) and their adjoining Primary Care Trusts (PCT). Ethical approval was obtained from the North West Multicentre Research Ethics Committee on the 21st June 2000; MREC Reference 00/8/34. Site-specific approval was obtained from the individual Local Regional Ethics Committees of the trial centres and their adjoining Primary Care Trusts (PCT). Approval was also obtained from the Caldicott Guardians (data controllers) of each of the participating PCTs. The sponsor is University College London.

Recruitment

2,000 to 10,000 women aged between 50 and 74 years were randomly selected on a regular (usually 3 monthly) basis from the age/sex registers held by the participating PCTs using specialised software commissioned from the NHS. Their contact details, date of birth, NHS number and GP details were forwarded as an electronic file to the Coordinating

Centre (CC). The software ensured that the women selected were flagged on the PCT register such that their details were not included in future downloads. These files were uploaded into the TMS. Women were then sent personal invitations (Appendix B) to join the trial along with a brochure outlining the objectives, design and inclusion/ exclusion criteria. Women who returned the tear away slip were sent appointments to attend for recruitment along with a detailed trial information sheet (Appendix C).

At the recruitment appointment, women viewed an information video and participated in a group discussion. They completed an 18-item recruitment questionnaire (Appendix D), which included information regarding eligibility. Those who were willing to participate in the psychosocial study were given a set of baseline quality of life questionnaires to complete and return to the researchers in a pre-paid envelope (Appendix E-J). All volunteers provided a baseline serum sample that was sent overnight at room temperature to the CC laboratory (Tumour Marker Laboratory, UCL) for storage.

The recruitment questionnaires were sent to the CC where they were scanned electronically using computerised intelligent character and optical mark reading software (Cardiff Software Inc, Teleform Elite version 8.1.1) which allowed rapid and accurate data entry. Any inconsistency or information not recognised by the data capture software was verified manually by trained data entry staff, who validated the computer-interpreted data.

The TMS checked for completeness of eligibility data. When data was incomplete, women were placed ‘on hold’ and letters requesting further information were automatically be sent. If the volunteer was placed ‘on hold’ because 12 months had not elapsed since her last

menstrual period or start of hormone replacement therapy, she was informed and included in the randomisation process when 12 months have elapsed from the relevant date. The TMS generated lists of women who had a family history suggestive of ‘increased risk of ovarian cancer’. Such women were individually contacted and eligibility confirmed. If they fulfilled criteria which put them at ‘increased risk’ of familial ovarian cancer, their GP was sent a letter requesting that the individual be referred to the Clinical Genetics department for risk assessment.

Consent

Separate written informed consent for the main trial, serum bank (Appendix K) and the psychosocial study was obtained (Appendix L). Each individual was also asked to sign a data protection form (Appendix M). A copy of the consent form was sent to the CC where it was checked and any exceptions to future use of data or samples noted on the TMS.

Randomisation

Once the TMS confirmed eligibility, participants were randomised to the control (C group), multimodal (CA125) (M group), or ultrasound (U) group in a 2;1:1 ratio using a computer generated random number algorithm. Randomisation was carried out as follows:

(1) The TMS allocated a set of 32 random numbers to each RC

(2) The lowest 8 were allocated to the M-group, the next 8 to the U-group and the remaining 16 to the C-group.

(3) Each successive volunteer within the RC was randomly allocated one of the random numbers and hence randomised into a group

(4) When all 32 random numbers had been used up a further set of 32 was generated

The randomisation was accomplished by using the visual basic randomisation statement and the Rnd function. Following this, letters were automatically printed and sent to the woman and her GP confirming eligibility and randomisation status.

Interventions

The screening strategies utilise two screening tests – serum CA125 interpreted the Risk of Ovarian Cancer (ROC algorithm and transvaginal ultrasound (TVS) of the ovaries.

Screening tests

For the measurement of serum CA125, a blood sample is taken in Greiner gel tubes (8 mL gel separation serum tubes; Greiner Bio-One 455071, Stonehouse, UK) at the trial centre and transported overnight at ambient temperature to the central laboratory. The blood is centrifuged at 4,000 rpm for 10 minutes and the serum separated. Excess serum is aliquoted and stored. Serum CA125 levels are determined by commercial enzyme immunoassay on the Roche EIA Elecsys 2010 system. All blood samples received >56 hours after venepuncture are discarded and repeat samples requested.

The second test used is transvaginal ultrasound (TVS). Where this is not acceptable to a volunteer, transabdominal ultrasonography is performed. The scans are performed by two levels of staff. Annual and repeat scans following an unsatisfactory annual scan are performed by Level 1 sonographers who are certified ultrasonographers, or trained midwives or doctors with experience in gynaecological especially transvaginal scanning. Level II scans are performed when an abnormality is detected on the annual screen. Level II sonographers are experienced gynaecologists or radiologists or senior

ultrasonographers (usually at superintendent grade in the NHS) with particular expertise in transvaginal ultrasonography. Majority of the scans were performed on a dedicated trial ultrasound machine.

Ovarian morphology and dimensions are assessed and volume determined using the formula for an ovoid (d1 x d2 x d3 x 0.532). Ovarian morphology is classified as

(1) Normal: if the ovary is of uniform hypoechogenicity and smooth outline with or without a single inclusion cyst or spots of calcifications. To classify as normal the inclusion cyst must be single, less than 10mms and should not distort the outline of the ovary.

(2) Simple cyst: A single, thin walled, anechoic cyst with no septa or papillary projections Is detected

(3) Complex: All non-uniform ovarian echogenicity excluding single simple cyst. If there are more than one cyst even if these are without septae or papillations and have thin wall with regular internal outline, ovarian morphology is classified as complex.

(4) Complex unchanged: Where the morphology is complex as described above but it has been noted and reviewed previously and has remained unchanged in appearance and size on follow up.

Detailed description of all features - the number and size of cysts, wall regularity, presence and thickness of septae, size of papillations and echogenicity of the fluid contents will be recorded (Appendix O). Definitions of ultrasound features and classification of cysts follow guidelines published by the International Ovarian Tumour Analysis (IOTA) group [9].

When ovaries are not visualised, ultrasonographer specify if a good view of iliac vessels is obtained or if the view is obscured (poor) due to bowel, fibroids, pelvic varicosities or other reasons.

Based on visualisation and morphology of the two ovaries, scans are classified as

(1) Normal: If both ovaries have normal morphology or simple cysts <60cm^3 or are not visualised but a good view of the iliac vessels is obtained, or are complex unchanged.

(2) Abnormal: If one or both ovaries have complex morphology or simple cysts >60 cm^3 or ascites (vertical pool in Pouch of Douglas >10mms)

(3) Unsatisfactory: If one or both ovaries are not visualised due to a poor view. The exception is those scans where one ovary is not visualised due to a poor view but the other ovary has abnormal morphology or a simple cyst >60 cm^3 or >5cms in diameter. In the latter case, the scan will be classified as abnormal.

Scan images are transferred weekly on magneto-optical discs for centrally archiving at the CC. TCs can request central review of the ultrasound image.

Multimodal Group (M) – screening strategy:

The multimodal strategy uses serum CA125 interpreted using the 'Risk of Ovarian Cancer' algorithm as the primary screening modality with TVS as the second line test[10]. The algorithms are derived from data collected in the numerous large prospective OCS studies undertaken in the 90s.[5,10-12] The first ROC determination is based upon a single measurement of CA125 and the woman's age specific incidence of ovarian cancer. Subsequent ROC determinations are based upon both the absolute CA125 level and the

rate of change in CA125 levels. The ROC summarises, in one number, the information about risk of ovarian cancer, thus simplifying the practical implementation of the screening protocol. It is anticipated that ongoing refinement of the screening algorithms will occur in the future in a manner analogous to refinement of the screening algorithms for Downs syndrome, cervical cancer and breast cancer.

Level I screen: This involves venepuncture and serum CA125 measurement. The assay results are directly uploaded into the TMS which calculates the Risk of Ovarian Cancer (ROC). Based on the ROC value women are triaged into three risk groups (Figure 2). which are managed as follows:

(1) Normal ROC (<1 in 3500): Volunteer returned to annual screening with the next blood test (Level I) on the one year anniversary of the randomisation date.

(2) Intermediate ROC (<1 in 1000 and ≥1 in 3500): Volunteer recalled for a repeat CA125 measurement in 12 weeks. The ROC is recalculated with management determined as for the initial ROC. Volunteers whose ROC remain intermediate after a total of three CA125 tests are referred for a Level II screen (see below).

(3) Elevated ROC (≥1 in 1000): Women were recalled for a Level II screen in 6-8 weeks with earlier screens arranged where there is a high index of suspicion.

Level II screen: This involved venepuncture for repeat CA125 assay and a TVS. Depending on the results of the Level II screen, there are three possible courses of action:

(1) Scan normal and the ROC normal or intermediate: Volunteer returned to annual screening with the next blood test (Level I) on the one year anniversary of the randomisation date.

(2) Scan normal and the ROC elevated OR scan unsatisfactory irrespective of ROC: Repeat Level II screen in 12 weeks with earlier screens arranged where there is a high index of suspicion.

(3) Scan abnormal irrespective of the ROC: Referral for clinical assessment with a view to surgery.

Women who have the Level II screen repeated are again triaged based on the findings to annual screening or clinical assessment.

Figure 2: Multimodal screening algorithm (updated 2009)

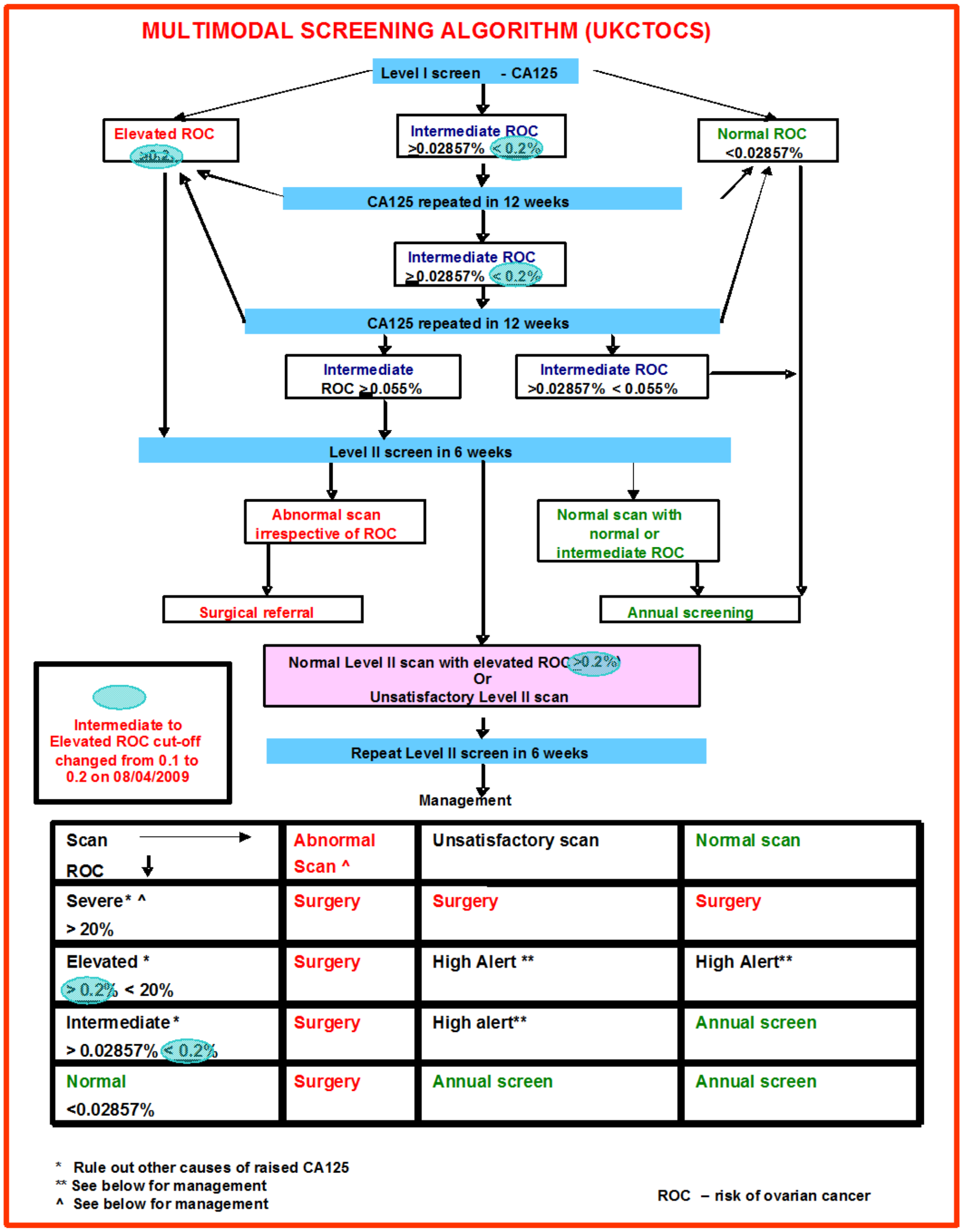

Scan → / ROC ↓	Abnormal Scan ^	Unsatisfactory scan	Normal scan
Severe* ^ > 20%	Surgery	Surgery	Surgery
Elevated * > 0.2% < 20%	Surgery	High Alert **	High Alert**
Intermediate * > 0.02857% < 0.2%	Surgery	High alert**	Annual screen
Normal <0.02857%	Surgery	Annual screen	Annual screen

High alert: Women who on repeat level II screen have either an unsatisfactory or a normal scan with intermediate or elevated CA125 undergo a further 'high alert' CA125 in 6 weeks. This is automatically generated and once performed, by the TMS. There are three possible courses of action as depicted below:

a) If ROC is <0.2%, the women are returned to annual screening by the TMS.

b) if the ROC is >0.2 and <10%, a repeat level II scan and CA125 is actioned in 8 weeks time. Each case is discussed with the PIs.

c) If ROC is >10%, a CT scan of chest and abdomen and a mammogram are performed. If appropriate, hysteroscopy is recommended. The results should be discussed with the PIs and prompt surgery needs to be undertaken.

Scan<br>ROC	Abnormal scan	Unsatisfactory scan	Normal scan
Severe*<br>>20%	Surgery	Surgery	Surgery
Elevated*<br>>0.2% < 20%	Surgery	High Alert	High Alert
Intermediate*<br>>0.02857% < 0.2%	Surgery	High Alert	Annual screen
Normal<br><0.02857%	Surgery	Annual screen	Annual screen

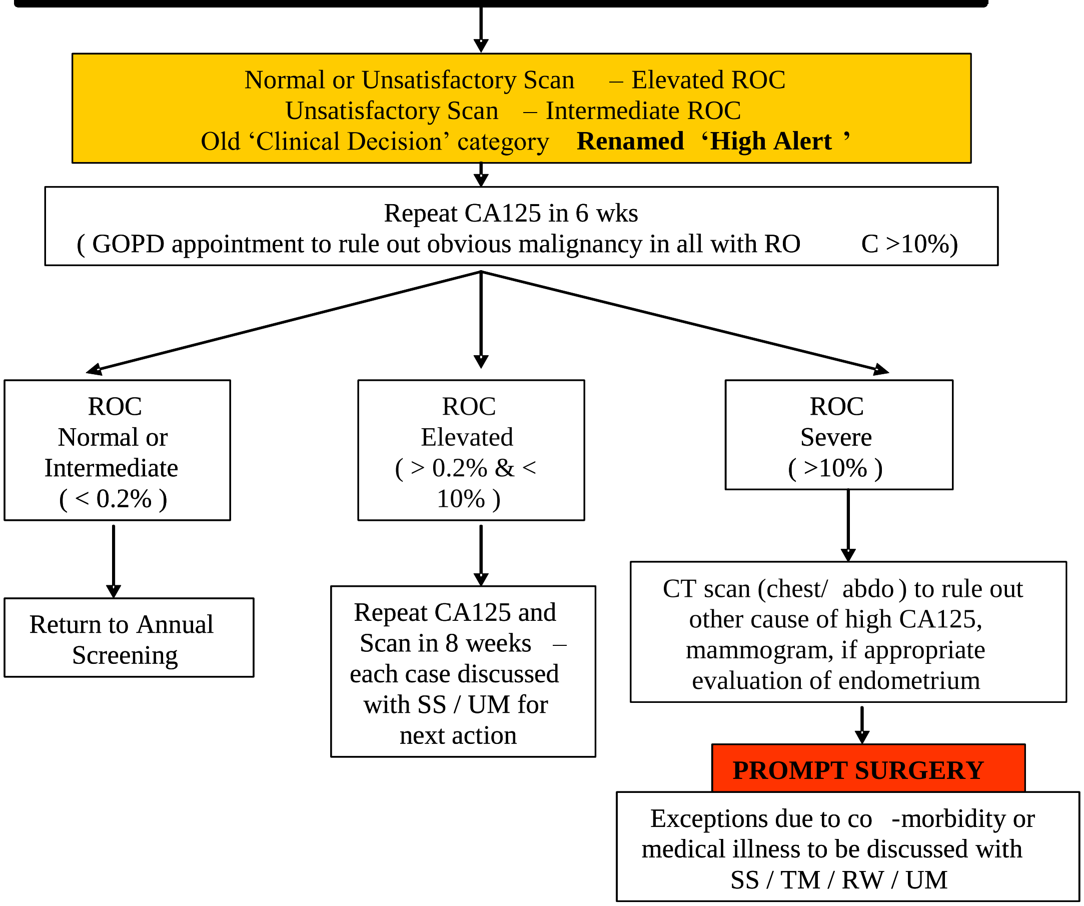

Severe ROC and/or abnormal scan: Women whose scan is unsatisfactory or normal with a corresponding ROC>20% or an abnormal scan with a ROC normal, intermediate, elevated or severe a letter is generated at the CC, which is sent to the Lead, Nurse and GP (via Nurse) to highlight the urgency of the results. The volunteer then undergoes a Gynae outpatient department review and is to have CT chest/abdomen/pelvis , mammogram, hysteroscopy if appropriate, the results of which need to be reviewed by PIs and prompt surgery needs to be performed.

Scan → ROC ↓	Abnormal scan	Unsatisfactory scan	Normal scan
Severe* ≥ 20%	Surgery	Surgery	Surgery
Elevated * ≥ 0.2% < 20%	Surgery	High Alert	High Alert
Intermediate* ≥ 0.02857% < 0.2%	Surgery	High Alert	Annual screen
Normal <0.02857%	Surgery	Annual screen	Annual screen

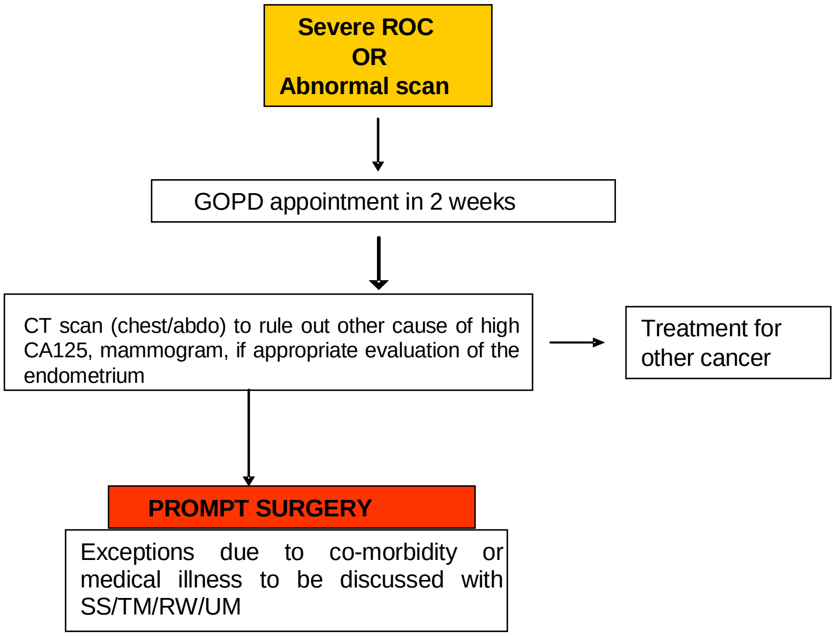

Ultrasound Group (U) – screening strategy:

The ultrasound strategy uses transvaginal ultrasound (TVS) as a first line test with repeat screening after 6-8 weeks if an abnormality is detected on initial testing.

Level I screen: Women randomised to the U group will have an annual TVS at their TC. Depending on the results of the scan, there will be three possible courses of action (Figure 3):

(1) Normal scan: Routine screening with an annual TVS (Level I) on the next anniversary (one year) of the randomisation date.

(2) Unsatisfactory scan: A repeat Level I scan in 12 weeks.

(3) Abnormal scan: Level II scan in 6-8 weeks. Earlier scans will be arranged where there is a high index of suspicion.

Level II screen: Depending on the results of the Level II screen, there will again be three possible courses of action:

(1) Normal Scan: Routine screening with an annual TVS (Level I) on the next anniversary (one year) of the randomisation date.

(2) Unsatisfactory scan: Repeat Level II screen in 6-8 weeks with earlier screens arranged where there is a high index of suspicion.

(3) Abnormal scan: Referral for clinical assessment.

Women who have repeat Level II screens will be triaged based on their findings to annual screening or clinical assessment.

The ultrasound screening procedures are described in greater detail in a separate document (the Ultrasound Screening Protocol).

Figure 3: UKCTOCS ultrasound algorithm

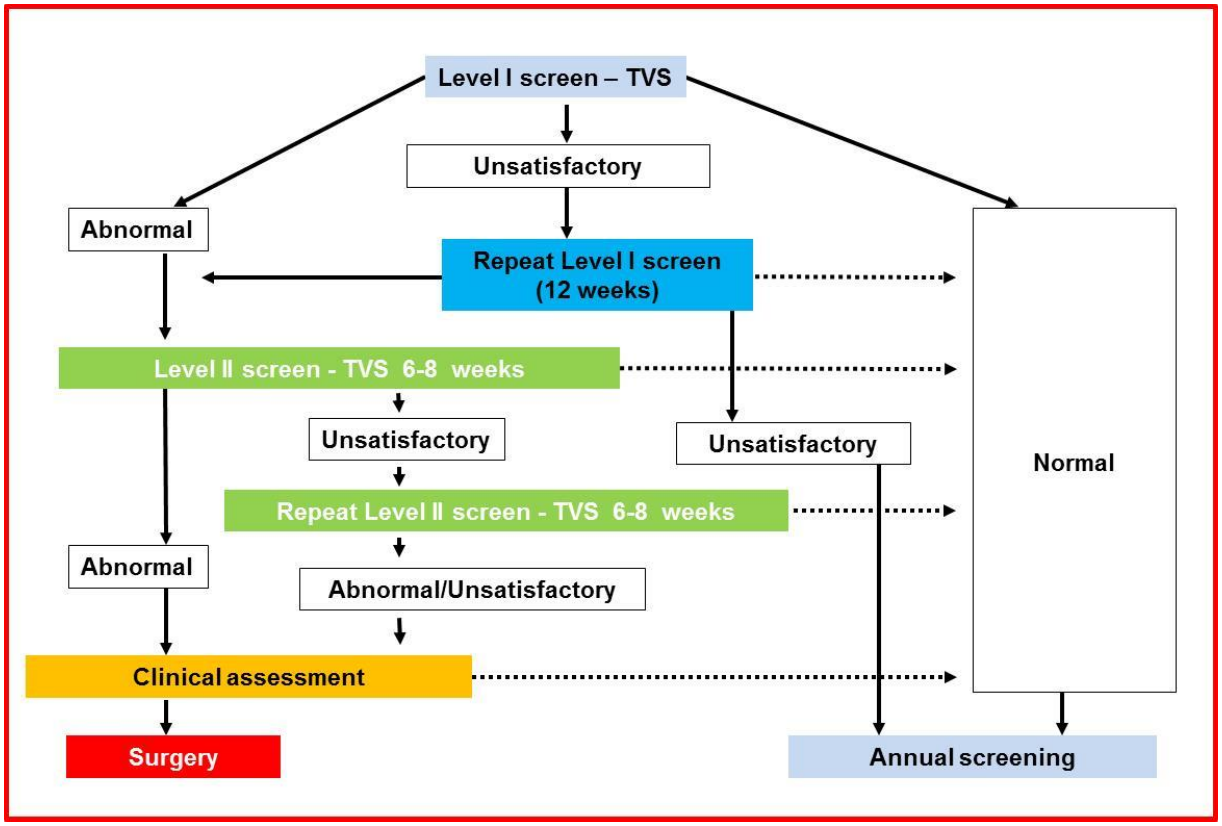

Counselling and support

All women on the trial will be given a leaflet explaining the trial design and objectives prior to recruitment. Staff with a detailed understanding of the study will be available at designated times at all TCs to answer volunteers' queries. All volunteers attending for a level II screen will be seen afterwards or contacted by a member of the local team who will explain the results of the scan and inform them of the timing and nature of expected follow-up. Pilot studies have suggested that a positive screen would be expected to have a positive predictive value for ovarian cancer of approximately 20-25% in the multimodal group and 5-10% in the ultrasound group.

Clinical assessment and further investigation:

Where the protocol requires clinical assessment, the TC refers the woman to a designated NHS clinician who carries out a clinical evaluation (history and physical examination) and arranges for further investigations as appropriate. These may include:

(1) Serum CA125 and/or other markers

(2) Repeat Ultrasound and Doppler with a senior consultant radiologist with a specialist interest in gynaecological oncology.

(3) CT / MRI of abdomen and pelvis.

(4) Serum FSH / oestradiol if multiple cysts present and hysterectomy has been performed in the past to establish if patient could be still ovulating.

(5) Mammogram to rule out breast cancer if CA125 profile is abnormal and no ovarian abnormality is detected on TVS.

In the M group, clinical assessment will include ruling out other causes of CA125 elevation (Appendix T). In the ultrasound group, some features of adnexal masses are associated with lower risk of OC and may be managed conservatively if the patient is asymptomatic and other clinical findings are not suggestive of malignancy. In women who have previously undergone a hysterectomy, there is an increased incidence of adhesions and peritoneal pseudocysts which maybe reported as ‘multilocular adnexal cysts’. In addition, significant co-morbidity and the views of the individual will be taken into consideration while making a final management plan

If there is a high index of suspicion, women undergo surgery. If the risk of a cancer is considered to be low or if the patient declines surgery, then conservative management with follow up is arranged.

Surgery:

The nature and timing of surgical intervention is decided at the consultation between the participant and the gynaecological oncologist / gynaecologist receiving the referral. Patients undergo routine investigations that are deemed appropriate for assessment of fitness for surgery. Some centres may use pre-operative assessment scoring systems to assess possible morbidity. It is to be noted that in the ultrasound group, a higher proportion of women who have had previous hysterectomy and/or endometriosis will be referred for surgery. The guidelines below represent appropriate management in the majority of cases. However, it is acknowledged that there will be circumstances where they cannot be adhered to and in such situations, management will depend upon the judgement of the clinician involved.

The primary aim of surgery will be to remove both ovaries and tubes for histopathological examination. However, there will be exceptions based on clinical grounds. One such occasion is where there are dense pelvic adhesions with increased risk of significant morbidity if dissection is undertaken. The clinician may opt to only remove the ovary found to have an abnormality on ultrasound and not dissect the contralateral 'normal looking' ovary. Hysterectomy adds to morbidity and will be avoided unless there are clear clinical indications. 'Normal looking' ovaries will be removed if associated with rising CA125 levels as malignancy has been detected in such ovaries in the pilot trial (unpublished data).

The approach to surgery will depend on the results of the pre-operative investigations and local surgical expertise. The primary intervention in most cases will be laparoscopy with the intention of performing a laparoscopic bilateral salpingo-oophorectomy. If clinical findings at laparoscopy are suggestive of ovarian cancer, or if laparoscopy is inappropriate because of technical difficulties, a laparotomy will be undertaken.

<u>Laparoscopy</u> - The procedure will be carried out using the surgeon's preferred technique. A thorough inspection of the whole of the abdominal cavity will be performed before aspirating any free fluid that is present and/or taking peritoneal washings. A bilateral salpingo-oophorectomy will be performed if technically possible and there are no features suggestive of ovarian cancer. Care will be taken to ensure removal of the entire ovary on each side. Aspiration of ovarian cysts will be avoided since a stage IA cancer may unwittingly be converted to a stage IC. When a histopathological diagnosis of ovarian cancer is made following a laparoscopic bilateral salpingo-oophorectomy, a formal staging laparotomy will be performed.

<u>Laparotomy</u> - If there is a clinical suspicion of ovarian cancer, the surgeon will carry out the laparotomy according to their usual practice in order to stage the disease and remove all macroscopic disease where possible. In addition, the clinician may decide to perform a laparotomy for bilateral salpingo-oopherectomy if this is deemed to be the best option for the individual based on clinical assessment.

Conservative management:

If the decision following clinical assessment, investigation and discussion with the woman is to manage the abnormality conservatively, then a follow up plan will be drawn up and

the CC informed. Most women will be followed up by a repeat TVS and possibly a serum CA125 assessment in three months and returned to annual screening if the findings are unchanged at review. Women will be advised to contact their GP if they have any symptoms as would happen in normal circumstances. At subsequent annual screens, no action will be taken if there is no change in the size or character of the previously detected adnexal lesion and it remains asymptomatic.

Management of Incidental findings

Ultrasound scans will sometimes reveal possible abnormalities that are outside the scope of this study. Any significant finding must be discussed with the lead researcher or other designated person. Each centre should draw up local guidelines for management of these findings. The most frequent example of this is a thickened endometrium. Women with endometrial thickness of >5mms are questioned for irregular bleeding. Suggested guidelines are that women should be referred if the endometrial thickness is > 10mms or endometrial thickness is >5 - <10 mms AND there is focal or cystic thickening or irregular bleeding. Volunteers with incidental findings will be referred to their general practitioner for further management.

Follow up

There will be two methods of follow up

Postal Questionnaires

All volunteers will be sent a postal questionnaire 3.5 years from the randomisation date and in 2014 following the planned 3-year follow-up phase of the trial post end of screening.

The first and second follow-up questionnaires **(**Appendices N and V**)** will ask for details of any hospital admission or serious illness since registration with the trial. Attempts will be made to contact volunteers who do not respond by sending further questionnaires by first class post. Where necessary further efforts to contact volunteers will be made by telephone or via the general practitioner.

It is possible that women in the control group will undergo screening elsewhere. The follow up questionnaires (Appendices N and V) will ask volunteers if they have undergone ovarian cancer screening of any kind in order to document the size of this problem. In addition, the method of diagnosis of ovarian cancer amongst women in the control group will be reviewed to establish whether any cases of OC were detected by screening.

The follow up questionnaires of women in the screen and control groups of the trial will be used to document gynaecological surgical procedures undertaken following clinical presentation (i.e. not to investigate screening results). Further details of these procedures will be obtained where possible from the appropriate hospital. It will then be possible to compare the number of surgical procedures and the frequency of surgical morbidity and mortality in the screened population and the control population.

Cancer Registry

All subjects participating in the study will be registered with either the for Health & Social Care Information Centre (HSCIC) for England and Wales or the Cancer Registry and Central Services Agency of Northern Ireland depending upon their place of residence. The computerised entry for each subject at the registry will be tagged to prompt notification to

the coordinating centre in the event of new cases of cancer and deaths in the study population. Women will be followed until 2024 to capture the full impact of screening on mortality due to ovarian cancer as this may not be evident in the few years immediately after screening has halted. The registry will also notify the CC of individual subjects who do not appear on the register so that further information required to trace their entry can be obtained or alternative methods of follow up employed. Individuals not identified on the register will be followed up by postal questionnaires only.

Outcome's review process

The purpose of the outcome committee's review is to verify reported or probable diagnosis of and death due to, ovarian/fallopian tube cancer.

1. Identification of potential ovarian/tubal cancer by coordinating centre

The Coordinating Centre (CC) is notified of new cases of ovarian/fallopian tube cancer (OC) and possible ovarian cancer by various sources.

The RC notifies the CC of all new cases of screen detected OC (and possible OC) and forwards relevant medical notes to the UKCTOCS senior research nurse or designated research associate. The RC will sometimes also notify the CC of screen negative and control arm cases where these cases are known to them. However, these volunteers do not always receive treatment at the RC hospital and will therefore not be identified through this method.

The Health &Social Care Information Centre (formerly NHS IC and prior to this, ONS) cancer registration and Death Certificates are two of the most important sources of information. Quarterly HSCIC updates are received electronically. After each_update, a query is run to identify potential new cases of ovarian/tubal cancer. The query flags up a list of ICD-10 codes that could be related to a diagnosis of ovarian or tubal cancer (see Appendix S-III Table1)).

The cohort has also been linked to the Hospital Episode Statistics (HES) in England. HES is a comprehensive dataset of volunteers' in- and out- patient hospital admissions and corresponding disease codes from 2001-2009 and 2003-2009, respectively. HES data on the women was received by the CC in 2010. The clinical diagnosis fields of the HES dataset are searched for the ICD-10 diagnosis codes, (see Appendix S-III) and in addition, for operative procedures.

New cases can also be identified through the follow-up questionnaires, which are sent to all volunteers in both the screening and control arms. The first questionnaires were sent out at approximately 3.5 years post randomisation to the trial and the second follow-up questionnaire in 2014. A query is run, after each upload of data from returned questionnaires, to capture new cases of OC and possible OC.

The research associate may also identify cases of ovarian cancer when querying volunteers, who have been withdrawn from the trial because their ovaries have been removed. In all instances where the CC is aware that the woman has had her ovaries removed surgically, a letter and a single page questionnaire are sent to the volunteer

requesting more information about her operation when ovaries were removed. Upon receipt of volunteer's reply, the research associate contacts the volunteer's hospital consultant for further information about the operation and whether a diagnosis of cancer was made. All attempts are made to obtain histology reports of the removed ovaries.

Occasionally, the volunteer (or her relative) may inform the CC directly of her ovarian cancer diagnosis. However, usually they notify the RC.

2. Review Process

Once a volunteer is "flagged up" as potentially having ovarian/fallopian tube cancer or having died due to ovarian/fallopian tube cancer, copies of all her medical notes relating to the cancer diagnosis/death are obtained from the corresponding regional centres, hospital(s) where treatment was received, GP surgeries, hospices and cancer registries.

The collected notes, arranged in chronological order and stripped of any evidence of volunteer allocation in the trial and/or screen-detection of the cancer, are presented to a member of the Outcomes Review Committee (ORC). Generally, at least three documents are required for ascertainment of an ovarian/fallopian tube cancer diagnosis or death due to ovarian/fallopian tube cancer. Where surgery has been performed, one of these documents must be a histology report. Other relevant information includes death certificates, NHS IC cancer registrations (CR), biopsy/cytology reports, diagnostic imaging results, multidisciplinary team meeting (MDT) summaries, discharge summaries, chemotherapy schedules and hospital letters. The clinical records, submitted to reviewers,

must also contain a clear letter of diagnosis by the clinical team such as an MDT review, letter to GP or hospital letter prior to chemotherapy commencement.

Upon review of the documents, the reviewer completes a trial cancer review form (CRF; see Appendix S-I)) and death review form (DRF, where applicable; see Appendix S-II)) for each volunteer. If the submitted information is deemed adequate and there is agreement between the reviewer's cancer diagnosis/cause of death assignment and either CR or death certificate (DC) registrations, the case will be considered "signed off". A signed off case will have primary cancer site, grade and stage (and cause of death, where applicable) verified. If the reviewer disagrees with either of CR/DC, the case will be forwarded to a second ORC member for an independent review. If the two ORC members are concordant in their assignments, the case will be signed off; otherwise, a third ORC member will independently review the case; and cancer diagnosis and cause of death will be signed off based on the majority reviewers' assignment. If there is disagreement between the assignments of the three ORC members, the case will be set aside for outcome committee's discussion, where two pathologists and two oncologists will review the case together and sign it off. Reviewers can also refer cases to outcome committee's discussion when they cannot independently ascertain cancer diagnosis and/or cause of death (Figure 4).

If the submitted information is insufficient for verification of cancer diagnosis/cause of death, the reviewer may request either additional information or histology slides for pathological review to determine stage/grade of diagnosed ovarian/tubal cancer. In such

cases, the UKCTOCS research associate will obtain the requested information or histology slides. Once the slides have been reviewed, the case will be re-submitted to the original reviewer with the additional histology report.

Figure 4: Outcome Review Process

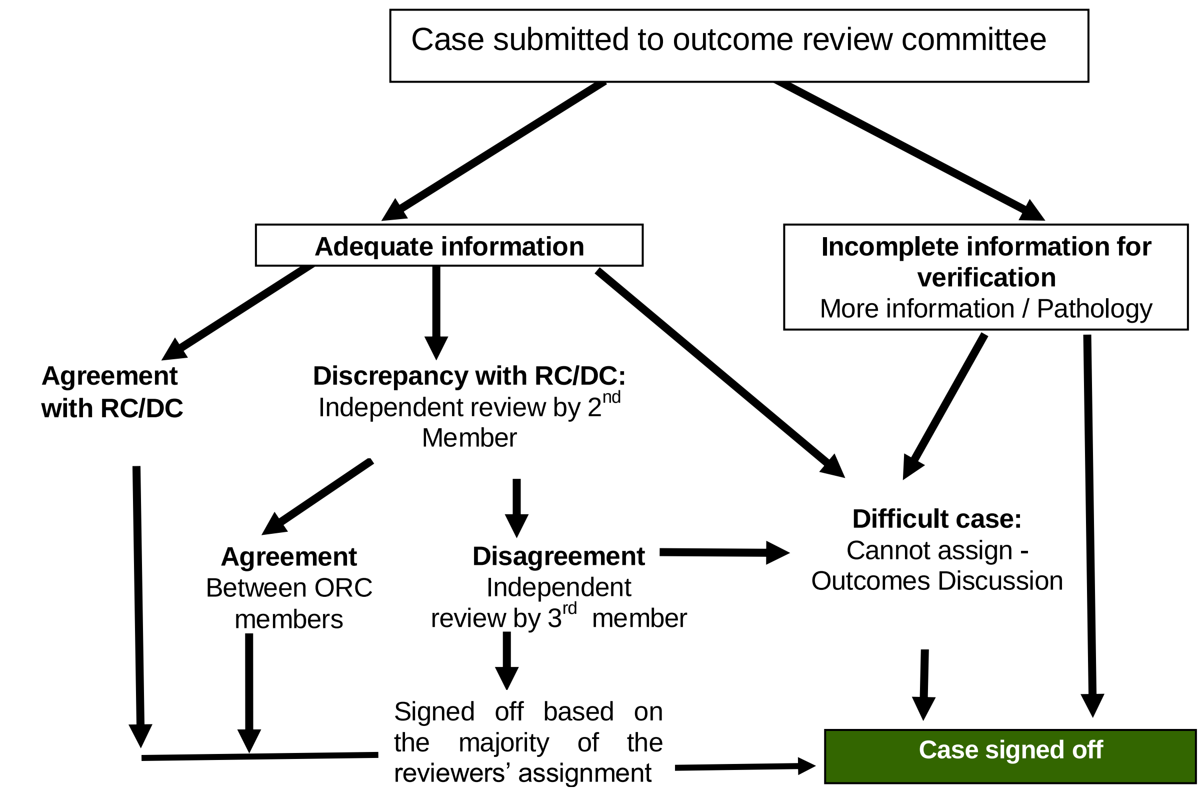

Disagreement refers to discordance with RC/DC re: C56/C57.0

RC: HSCIC Registration

Morbidity and serious adverse reactions

A copy of the operative and other relevant pages of the hospital notes of women who undergo trial surgery will be obtained. A senior trial gynaecological oncologist blinded to

the randomisation group will confirm all surgical complications. Suspected unexpected serious adverse reactions will be reported within 7 days using a defined protocol and specific form (Appendix U). Complications related to screening and resulting from trial surgery will be presented regularly to the Data Monitoring and Ethics Committee.

Psychosocial study:

All consenting women will complete psychosocial baseline questionnaires at randomisation (Appendices E-J) These include an examination of their knowledge, beliefs and attitudes to ovarian cancer screening and standardised questionnaires including the General Health Questionnaire 12 (GHQ12), Speilberger Trait Anxiety Inventory (STAI) and Fallowfield's Sexual Activity Questionnaire (SAQ). This information is entered into the study database if the women are subsequently randomly chosen for longitudinal follow-up (250 women in each screening arm and 500 in the control arm) or if any woman's screening results are not straightforward. Volunteers will complete a set of postal questionnaires annually for 6 years to assess their emotional well-being, psychological functioning, sexual activity, and the acceptability of the screening process after each screen. Women will also complete questionnaires if they undergo surgery and again if the surgery reveals that they have ovarian cancer. These women will then be followed longitudinally at 6 months post-surgery.

Serum bank:

Excess serum will be stored for future use in ethically approved studies for early detection and treatment of disease. A semi-automated system will aliquot this serum into 500 microlitre straws which will then be heat sealed, barcoded and stored in special containers

in liquid nitrogen tanks. Once filled, the tanks will be transported to an offsite cryonic biorepository.

Statistical analysis plan

The primary outcome measure in UKCTOCS is ovarian cancer (OC) mortality and the primary comparison is based on an ‘intention to treat’ analysis between control (C) group and each of the screened groups (M and U). In the UK, there are approximately 37 deaths from ovarian cancer per 100,000 women aged 50-74 per year. Assuming an attrition rate of 4% per year (75% of patients followed up for the full 7 year period) gives a mean of 6 person years of observation for each patient randomised, and thus an expected number of deaths from ovarian cancer in the control group of 222 per 100,000 women randomised. The event of interest, death from ovarian cancer, is rare and so it is assumed that the observed number of deaths in each group follows a Poisson distribution. Under these assumptions, a total sample size of 200,000 (100,000 controls, 50,000 in each screening group) followed for 7 years from randomisation would give > 80% power at the (two-sided) 5% significance level to detect a reduction of 30% in each of the screened groups (C vs M or U).

The operating characteristics of the two screening arms are different. It is important to note that if one comparison is significant and the other is not, this result does not necessarily imply that one method is significantly better than the other. Only the direct comparison between the two methods will address this issue. The trial has at least 70% power to detect a difference in OC mortality between the two screening groups of 30% or more. However, if as anticipated the difference in OC mortality between the two screened groups

is modest then this study will have limited power to detect such differences e.g. 35% power to detect a difference of the order of 20%. The choice of screening strategy will then be based on other outcome measures such as sensitivity, positive predictive value, morbidity, quality of life and health economics.

Randomisation to UKCTOCS was completed in October 2005 and in 2007, with women having completed a median of approximately 3.5 years in the study, it was possible to review mortality in the control arm. The expected number of control group deaths (ED) based on mortality statistics from ONS 2004 was recalculated with more precision taking into consideration several factors.

(1) Age-group specific mortality rates based on the age at randomisation of the 202,638 women and the increasing age of the women as the trial progresses

(2) Reduction in OC deaths due to the eligibility criteria in the early years of the trial. Deaths in any particular year from ovarian cancer occur largely in women diagnosed with the disease in the preceding 2-3 years. Based on eligibility criteria women with ovarian cancer were not able to join the trial.

(3) True attrition rate to follow up – Follow up was via ONS flagging and few women withdrew from flagging even if they withdrew from screening. As a result, the original 4% annual attrition rate was found to be conservative. So in place of an attrition factor, an adjustment was made for a mortality factor of deaths from other causes that reduces the effective population at risk from ovarian cancer.

This analysis identified a shortfall in number of observed deaths (OD) in UKCTOCS participants compared to age adjusted expected deaths (ED). The overall Standardised

Mortality Ratio (SMR - ratio of observed versus expected deaths expressed as a percentage) was 31.6 (95% CI 30.1, 32.7) suggesting that there was a 'healthy volunteer effect (HVE). The SMR for OC mortality was 58.31(95% CI 40.4, 81.5). When the SMR was calculated for each year from randomisation, it gradually increased suggesting that the HVE became less of a factor as time from randomisation increased. On this basis, a reasonable estimate of the SMR for OC mortality for the trial was taken as 65. An OC SMR of 65 would have a significant impact on power calculations. It would have reduced the power to detect a 30% reduction in mortality between control and one of the screen arm (C vs M or U) to 53.7% versus the original estimate of 80%.

After exploring various options to increase sample size, it was finally decided to alter the trial design to extend follow up on all women to 31st December 2014. Based on randomisation date, this would result in individual women being in the trial for 9 to 13 years. In the study groups (M and U groups) the number of annual screens was extended till 31st December 2011 such that women underwent 7 to 11 screens (compared to the original 6 screens) depending on date of randomisation. Women in the study groups would be followed up for at least 3 years from their last annual screen. The design provides the power to detect a 30% reduction in mortality between control and one of the screen arm (C vs M or U) of >80% as originally envisaged.

Primary analysis:

The primary analysis will be a direct comparison of the mortality rate of ovarian cancer:

- between the control arm and multimodal arm
- between the control arm and ultrasound arm.

Events of interest will be those deaths signed off by Outcome Review as primarily caused by ovarian cancer (ICD-10: C56) or tubal cancer (C57.0) or undesignated ovarian/tubal/peritoneal cancer. The analysis will be based on 'intention to treat', so that no allowances for non-compliance to screening will be made. Similarly for any 'contamination' that occurs. A Cox regression model will be used to model the difference in mortality rate between the control arm and each individual screen arm. Several sensitivity analyses will be performed to validate the primary outcome result.

Secondary analyses: This will include assessing the performance characteristics of the screening modalities, namely their sensitivity, specificity and positive predictive value, compliance with screening and complication rates resulting from trial surgery using the conventional ratios with appropriate confidence intervals.

There will be an interim analysis performed at the point where half the expected number of control group deaths due to OC (111) is reached. This will include all primary and secondary endpoints detailed above as well as intermediate endpoints for OC mortality such as stage of OC and lead time (mean duration of preclinical disease) estimated from the ratio of prevalence (at the first screen) and incidence (annual) rates of OC.

The economic analysis will be undertaken on the calculated cost-effectiveness ratios that are crucial to the trial. It will include both a within trial analysis and a modeling analysis. The within trial analysis will consider the cost-effectiveness ratios relating to protocol driven resource use. The modeling analysis will take into account standard practice which would reflect the implementation of a National Screening Programme (as this may differ

from study protocol resource utilisation), and extrapolation of trial results over a longer time frame to include lifetime benefits.

Exploratory analysis: With women followed for up to 13 years in the first instance until 2014 and during extended follow-up until 2024 there is great scope to produce an extensive array of exploratory analyses. Characteristics of the randomised population will be compiled using standard descriptive statistics of the (baseline) recruitment data. In addition, the use of postcodes can provide relevant deprivation indices. These will be further enhanced by data obtained via from postal questionnaires. The collection from HSCIC of incidence data of all other cancer types and mortality data of all causes allows investigation of numerous potential links between incidence and mortality and the population characteristics.

The vast amount of data generated from the screens themselves, pertaining to ultrasounds, blood tests, surgery outcomes and histopathology results will be analysed as 1) a quality control of the screens (e.g. percentage of ovaries ‘seen’ by ultrasound sonographers) 2) as worthwhile scientific data in its own right (e.g. average ovarian volume) and 3) as further possible explanatory variables in studies on other cause mortality and incidence.

Predefined subgroups: The benefit of screening, and in particular the positive predictive value (PPV), will depend on the absolute level of risk for the population. Women at a particularly high risk of developing OC due to familial predisposition are excluded from this trial. Apart from differences between age groups, long-term use of oral contraceptive pills

and number of pregnancies are known to be factors influencing the risk of developing OC. The major subgroups to be explored and contrasted will be those defined as being ‘low risk’ and ‘intermediate risk’ groups within this population. The ‘low risk’ group will be defined as women with ≥3 full-term pregnancies or ≥5 years use of oral contraceptives; the ‘intermediate risk’ group will be those women with <3 full-term pregnancies and <5 years use of oral contraceptives.

Start date: April 2001

End of screening date: 31st December 2011

Censorship for primary mortality analysis: 31st December 2014

Reporting date for above: June 2015

End of follow-up: December 2024

Publication policy

The credit for results addressing the core hypotheses of this collaborative effort lies with all the members of the UKCTOCS team and authorship will include all names if investigators, researchers at the coordinating centre and trial centre leads where possible. If the journal does not permit this due to the number of authors, then the paper will be published under the acronym - UKCTOCS group. The data and samples available offers numerous opportunities for secondary studies which will be performed either by members of the UKCTOCS team or in collaboration with other investigators. Authorship credit will be based on the usual criteria. The entire UKCTOCS group will be acknowledged on all such publications.

Trial Steering Committee (Figure 5)

Independent members - Professor David Luesley (Chair), Department of Gynaecological Oncology, The Birmingham Nuffield Hospital, Birmingham; Ms Louise Bayne, Ovacome, London; Professor Jack Cuzick, Department of Epidemiology, Mathematics and Statistics, Cancer Research UK Clinical Centre at Queen Mary University, London; Professor Julietta Patnick, NHS Cancer Screening Programmes.

Funding agency representatives –Trials and Studies Coordinating Centre (NETSCC) Programme Manager; Head of Clinical and Population Research Funding, CRUK

Investigators **-** Professor Ian Jacobs, Professor Usha Menon, Professor Mahesh Parmar, Professor Lesley Fallowfield,

Data Monitoring and Ethics Committee (Figure 5)

Professor Peter Boyle (chair), Director, International Agency for Research on Cancer (IARC), Lyon, France; Professor Peter Heintz, Department of Gynaecology and Oncology, University Medical Centre, Utrecht, The Netherlands; Dr Susanne Kjǽr, Danish Cancer Society Research Centre, Division for Cancer Epidemiology, Copenhagen, Denmark; Professor Edward Trimble, Department of Gynaecology and Obstetrics, The John Hopkins Hospital, Baltimore, MD, US

Trial Management Committee (Figure 5) Professor Ian Jacobs; Professor Usha Menon (chair); Professor Mahesh Parmar; Dr Steve Skates; Professor Stuart Campbell; Mr Tim Mould; Mr Rob Woolas; Dr Aleksandra Gentry-Maharaj; Dr Matthew Burnell; Dr Andy Ryan; Dr Jatinderpal Kalsi.

Ultrasound Sub-Committee (Figure 5)

Professor Usha Menon (chair); Professor Stuart Campbell; Professor Nazar Amso; Mr Mourad Seif; Dr Gillian Turner; Dr Carol Brunell (consultant radiologist); Dr Rani Rangar ; Mrs Gwendolen Fletcher; Dr Aleksandra Gentry-Maharaj; Dr Matthew Burnell Ms. Susan Davies; Dr Jatinderpal Kalsi

Outcomes Committee (Figure 5)

Dr Naveena Singh **(Chair)** , Department of Pathology, Bart's and the London NHS Trust, London; Ms Karina Reynolds, Department of Gynaecological Oncology, Bart's and the London NHS Trust, London; Dr Elizabeth Benjamin, Department of Pathology, University College Hospital, London (since 2007), Professor Martin Widschendter, Department of Gynaecological Oncology, UCL.

Coordinating Centre Teams (Figure 5)

Main trial: Gynaecological Cancer Research Centre, University College London Elizabeth Garrett Anderson Institute for Women's Health, London, United Kingdom

Andy Ryan (database manager), Aleksandra Gentry-Maharaj (postdoctoral fellow), Susan Davies (senior research nurse), Anne Dawnay (consultant biochemist), Jatinderpal Kalsi (UKCTOCS project manager), Matthew Burnell (statistician), Sheila Spicer.

Previous team members: Jeremy Ford (laboratory manager), Richard Gunu (Snr BMS), Mariam Habib (postdoctoral fellow), Lucy Connelly (clerk), Theresa Goodall (MLSO), Mumtaz Ahmed (MLSO), Kirstin Tamm (MLSO), Josephine Cunningham (MLSO), Susan Grant (postdoctoral fellow), Rachel Hallett (postdoctoral fellow), Sara Lewis (research assistant), Susan Philpott (research assistant), Aarti Sharma (clinical fellow), Karen Sibley

(project manager), Tracy Bridgeford. Charlotte Spicer, Lisa Sterry, Yvonne Wold (clerical staff); Nuno Alves, Mary Pamboris, Tasneem Akbar, (laboratory technicians).

Psychosocial study: Sussex Health Outcomes Research & Education in Cancer (SHORE-C), University of Sussex, Sussex, UK.

Justine Kilkerr, Clare Coxon, Louise Parlour. Barrett J, Langridge C,

Team also includes: Jenkins V, Cardiovascular Epidemiology Unit, Department of Public Health & Primary Care, University of Cambridge, Cambridge, UK, Farewell V, Medical Research Council Biostatistics Unit, Institute of Public Health, Cambridge

Previous team member: Hazel Beveridge

Figure 5: Trial organisation

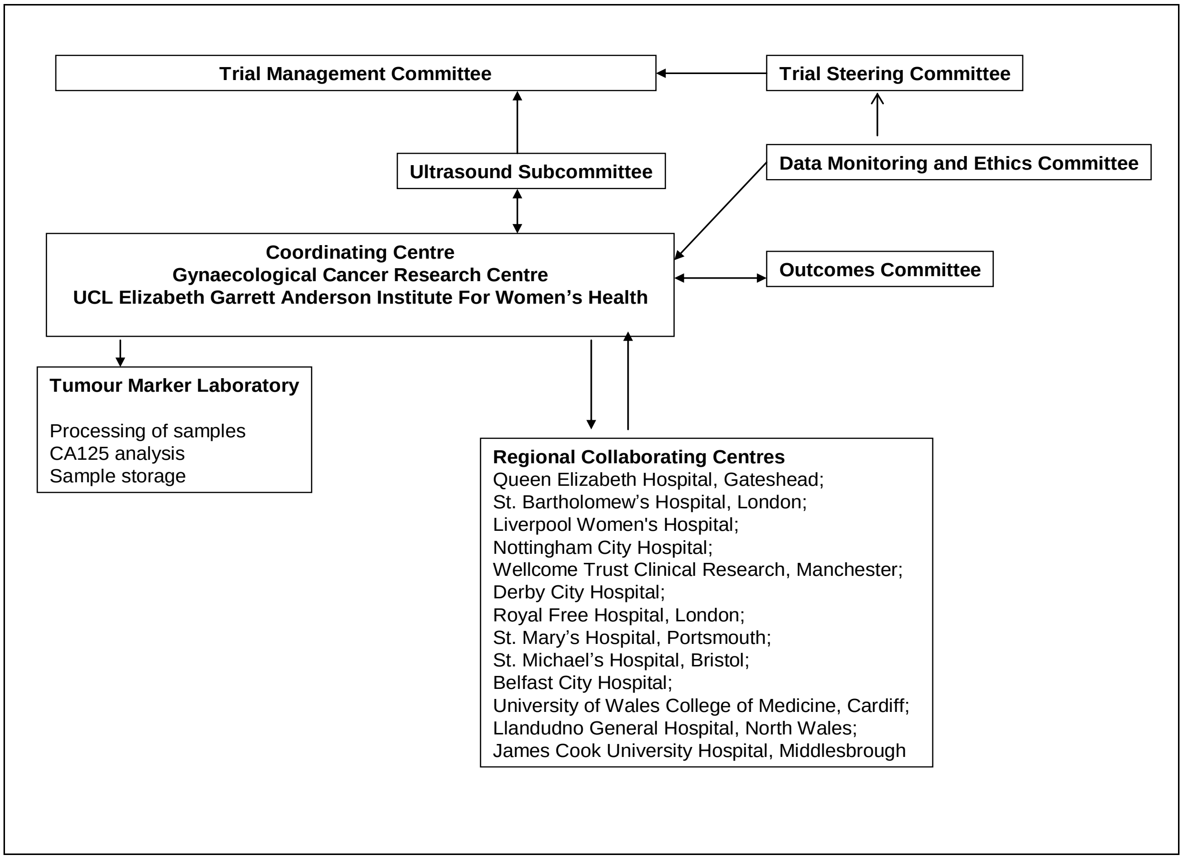

Trial Centres and Leads

1. Northern Gynaecological Oncology Centre, Queen Elizabeth Hospital, Gateshead:

Lead Investigator: Mr Keith Godfrey.

Former Lead Investigators: Mr Tito Lopez, Mr John Monaghan.

2. Department of Gynaecological Oncology, St. Bartholomew's Hospital, London.

Joint Lead Investigators: Professor Usha Menon and Mr David Oram.

3. Department of Gynaecology, Liverpool Women's Hospital, Liverpool.

Lead Investigator: Mr Jonathon Herod.

4. Department of Gynaecological Oncology, Nottingham City Hospital, Nottingham.

Lead Investigator: Ms Karin Williamson.

5. Obstetrics and Gynaecology, St. Mary's Hospital, Manchester

Lead Investigator: Mr Mourad Seif.

Previous Lead Investigator: Ms Karina Reynolds

6. Department of Gynaecological Oncology, Derby City Hospital, Derby.

Lead Investigator: Mr Howard Jenkins (since 2008).

Previous Lead Investigator: Mr Ian Scott (until 2008).

7. Department of Gynaecology, Royal Free Hospital, London

Lead Investigator: Mr Tim Mould.

8. Department of Gynaecological Oncology, St. Mary's Hospital, Portsmouth.

Lead Investigator: Mr Robert Woolas.

9. Department of Gynaecological Oncology, St. Michael's Hospital, Bristol

Lead Investigator: Mr John Murdoch.

10. Department of Gynaecological Oncology, Belfast City Hospital, Belfast.

Lead Investigator: Mr Stephen Dobbs.

11. Department of Gynaecology, University of Wales College of Medicine, Cardiff.

Lead Investigator: Professor Nazar Amso.

12. Department of Gynaecological Oncology, Llandudno Hospital, North Wales.

Lead Investigator: Mr Simon Leeson.

13. Department of Gynaecological Oncology, James Cook University Hospital, Middlesbrough.

Lead Investigator: Mr Derek Cruikshank.

Details of UKCTOCS Regional Centre Staff are stored locally and on the trial database.

Funding

The main trial is core funded by the Medical Research Council, Cancer Research UK and the Department of Health with additional support from Eve Appeal, Special Trustees Barts and the London and Special Trustees University College London Hospital. The Psychosocial Study is funded by the Medical Research Council.

References

1. Office for National Statistics. Cancer Statistics registrations: Registrations of cancer diagnosed in 2004, England *Series MB1 35* 2004;**24**.

2. Berrino F, De Angelis R, Sant M, et al. Survival for eight major cancers and all cancers combined for European adults diagnosed in 1995-99: results of the EUROCARE-4 study. *Lancet Oncol* 2007;**8**(9)**:**773-83.

3. Goff BA, Mandel L, Muntz HG, Melancon CH. Ovarian carcinoma diagnosis. *Cancer* 2000;**89**(10)**:**2068-75.

4. Surveillance Epidemiology and End Results (SEER) Program (www.seer.cancer.gov). Mortality - All COD, Public-Use With State, Total U.S. (1969-2003). In: National Cancer Institute D, Surveillance Research Program, Cancer Statistics Branch, ed. SEER*Stat Database: Surveillance, Epidemiology, and End Results (SEER) , National Cancer Institute, 2006.

5. Jacobs IJ, Skates SJ, MacDonald N, et al. Screening for ovarian cancer: a pilot randomised controlled trial. *Lancet* 1999;**353**(9160)**:**1207-10.

6. van Nagell JR, Jr., DePriest PD, Reedy MB, et al. The efficacy of transvaginal sonographic screening in asymptomatic women at risk for ovarian cancer. *Gynecol Oncol* 2000;**77**(3)**:**350-6.

7. Sato S, Yokoyama Y, Sakamoto T, Futagami M, Saito Y. Usefulness of mass screening for ovarian carcinoma using transvaginal ultrasonography. *Cancer* 2000;**89**(3)**:**582-8.

8. Bell R, Petticrew M, Luengo S, Sheldon TA. Screening for ovarian cancer: a systematic review. *Health Technol Assess* 1998;**2**(2)**:**i-iv, 1-84.

9. Timmerman D, Valentin L, Bourne TH, Collins WP, Verrelst H, Vergote I. Terms, definitions and measurements to describe the sonographic features of adnexal tumors: a consensus opinion from the International Ovarian Tumor Analysis (IOTA) Group. *Ultrasound Obstet Gynecol* 2000;**16**(5)**:**500-5.

10. Menon U, Skates SJ, Lewis S, et al. Prospective study using the risk of ovarian cancer algorithm to screen for ovarian cancer. *J Clin Oncol* 2005;**23**(31)**:**7919-26.

11. Jacobs IJ, Skates S, Davies AP, et al. Risk of diagnosis of ovarian cancer after raised serum CA 125 concentration: a prospective cohort study. *BMJ* 1996;**313**(7069)**:**1355-8.

12. Jacobs I, Davies AP, Bridges J, et al. Prevalence screening for ovarian cancer in postmenopausal women by CA 125 measurement and ultrasonography. *Bmj* 1993;**306**(6884)**:**1030-4.

Appendix A - GP Fact Sheet

UK COLLABORATIVE TRIAL OF OVARIAN CANCER SCREENING (UKCTOCS)

**Funding bodies**	MRC, NHS R&D, CRC, ICRF, Gynaecology Cancer Research Fund (GCRF)
**Design/Intervention**	Randomised controlled trial with three arms:<br>(1) A control group (no screening).<br>(2) A multimodal group (annual screening with serum CA 125 as the primary test and CA125 and ultrasound as the secondary test).<br>(3) An ultrasound group (annual screening with ultrasound as the primary test and repeat ultrasound in 6-8 weeks as the secondary test).
**Objectives/aims:**	**Objective 1**<br>• To establish the impact of screening on ovarian cancer mortality<br>**Objective 2**<br>• To determine the physical morbidity of ovarian cancer screening.<br>• To determine the resource implications of screening.<br>• To record the psychological consequences of screening.<br>• To assess the feasibility of population screening for ovarian cancer as reflected by uptake of invitations and compliance rates with annual screening.<br>• To compare the performance of two screening strategies for ovarian cancer<br>**Objective 3**<br>To establish a serum bank for future assessment of novel tumour markers.
**Endpoints/primary outcomes:**	Ovarian cancer mortality at 7 years after randomisation.
**Inclusion/Exclusion criteria:**	**Inclusion criteria**<br>1. Age 50-74 years:<br>2. Postmenopausal: Either (a) >12 months amenorrhoea following a natural menopause or hysterectomy, or (b) >12 months of hormone replacement therapy (HRT) commenced for menopausal symptoms.<br>**Exclusion criteria**<br>(1) History of bilateral oophorectomy.<br>(2) Currently active non-ovarian malignancy. Women who have a past history of malignancy will only be eligible if (a) they have no documented persistent or recurrent disease and (b) have not received treatment for >12 months.<br>(3) Women who have had an ovarian malignancy in the past.<br>(4) Women at high risk of ovarian cancer due to familial predisposition as defined by the eligibility criteria for the UKCCCR Familial Ovarian Cancer Screening Study.<br>(5) Women participating in other ovarian cancer screening trials
**Recruitment**	Women aged 50-74 will be sent invitations to participate from Heath Authority age/sex registers over a 3 year period. **Note that women cannot volunteer or be referred to participate in this trial**
**Planned sample size:**	200,000 postmenopausal women, 100,000 randomised to the control arm, 50,000 randomised to multimodal screening, 50,000 randomised to ultrasound screening.
**Follow up:**	1. Postal questionnaire: All study participants will be asked to complete a health questionnaire midway through the trial and at the end of their participation in the trial (i.e. at 3.5 and 7 years after randomisation).<br>2. National Health Service Central Register: All study participants will be flagged using their NHS number through either the National Health Service Central Register for England and Wales or the Cancer Registry of Scotland depending upon their place of residence.
**Trial duration:**	January 2001 - December 2010
**Contact details:**	UKCTOCS Co-ordinating Centre<br>Gynaecological Cancer Research Unit<br>Institute of Women's Health, UCL<br>149 Tottenham Court Road<br>London, W1T 7DN<br>Tel: 020 7380 6925, Fax: 020 7380 6929<br>http://www.ukctocs.org.uk

UKCTOCS
United Kingdom Collaborative Trial of Ovarian Cancer Screening

Date as Postmarked

Dear **Vol Ref:**

We are writing to ask if you would like to help us to test a new screening programme for ovarian cancer. Over 4000 women per year in the UK die from ovarian cancer and we hope to find out if screening can save lives.

The study is called UKCTOCS (United Kingdom Collaborative Trial of Ovarian Cancer Screening). We need to involve 200,000 women, half of whom will be screened and half will have the usual medical care. Screening will involve either a blood test or an ultrasound scan.

You have been randomly selected from women aged 50 to 74 from your local health authority register to ask if you would be interested in taking part in this study. Please could you fill in the tear-off slip at the bottom of this letter and return it to let us know whether or not you would like to take part (no stamp needed). We will send you more information and an appointment to attend the above centre if you decide to take part.

We look forward to receiving your reply slip.

Yours sincerely

UKCTOCS Team

Send reply to:
Gynaecological Cancer Research Centre
FREEPOST LON21030
London W1T 7BR

WE WOULD BE VERY GRATEFUL IF YOU WOULD RETURN THIS SLIP
Ref: Error! Bookmark not defined.
Error! Bookmark not defined. of Error! Bookmark not defined., Error! Bookmark not defined., Error! Bookmark not defined., Error! Bookmark not defined. Error! Bookmark not defined.

I would like to take part in the UKCTOCS study | **YES** ☐ | **NO** ☐ |

I would like to take part but cannot because:

I am still having periods	☐	More than one person in my family has ovarian cancer	☐
I have had both ovaries removed	☐	I am taking part in another ovarian cancer screening trial	☐
I have had ovarian cancer	☐	I am currently being treated for cancer	☐

Signed……………………………………… Date…………………………………..

United Kingdom Collaborative Trial of Ovarian Cancer Screening (UKCTOCS)

You are being asked to take part in a research study of screening for ovarian cancer. Invitations are being sent to women in your region who are not known to have ovarian cancer. Before you decide it is important for you to understand why the research is being done and what it will involve. Please read the following information carefully and discuss it with friends, relatives and your GP if you wish. Ask us if there is anything that is not clear or if you would like more information. Take time to decide whether or not you wish to take part.

Thank you for reading this.

What is the purpose of the study?

Ovarian cancer is the fourth commonest cause of death from cancer amongst women in the UK. The majority of women who develop this cancer have few symptoms until it has spread outside the ovaries. By then, the cancer is much more difficult to treat and many women will die as a consequence of the cancer. In contrast, treatment is more successful and the outlook can be good for the small proportion of women diagnosed before the ovarian cancer has spread. The purpose of this study is to determine whether screening will detect ovarian cancer at an early stage when treatment is more effective and therefore reduce the number of deaths due to the disease.

Large studies performed by our research team during the last decade have developed and refined two methods of screening. One method uses ultrasound scanning, similar to the scanning used in pregnancy, to check for any enlargement or abnormality of the ovaries. This trial will use a method of ultrasound scanning called transvaginal scan whereby a probe is inserted into the vagina to see the ovaries. This method of scanning gives a much clearer picture of the ovaries than a transabdominal scan, where the probe is placed on the abdomen. Transvaginal scan should be no more uncomfortable than having a smear test. If, however, transvaginal scanning is not possible or acceptable a transabdominal scan will be performed. The second method involves a blood test to measure a substance called CA125, which is released at higher levels into the blood in women with ovarian cancer. Using these tests it seems likely that over 80% of women with ovarian cancer can be identified before they have symptoms. This current very large trial will answer the question whether early detection of ovarian cancer, using these tests can save the lives of women who have ovarian cancer.

The study will also assess the cost implications of the screening methods to the National Health Service (NHS), what anxieties and fears being screened may raise and what complications might arise as a result of screening. This information will be used to make a decision about whether an NHS national screening programme for ovarian cancer should be introduced.

The study will last for 10 years. If you agree to take part you will be followed up for 6 years.

Why have I been chosen?

We need a total of 200,000 women, aged 50-74 years to take part in this study. You have been invited as you belong to this age group. Your details were obtained from the age/sex register of your health authority.

Do I have to fulfil any other criteria to take part?

In order to be eligible to take part you should:

- Be 50-74 years of age.
- Postmenopausal, i.e. you should not have had a period within the past 12 months if you are not taking hormone replacement therapy (HRT). However, if you are taking HRT, you are still eligible to take part, if you have taken it for one year or more.
- Not have had both your ovaries removed at surgery. You can still take part if only one ovary was removed.
- Not have had surgery, chemotherapy or radiotherapy for any type of cancer within the past 12 months.
- Not currently be taking part in any other screening programme for ovarian cancer.
- Not belong a to a family with a high risk of familial ovarian cancer. Such families have:

1. Two or more first degree relatives (mother, sister, daughter) with ovarian cancer.
2. One first degree relative with ovarian cancer and one first degree relative with breast cancer diagnosed under 50 years of age.
3. One first degree relative with ovarian cancer and two first or second degree (grandmother, granddaughter, aunt or niece) relatives with breast cancer diagnosed under 60 years of age.

4. Three first degree relatives with cancer of the large intestine or bowel, with at least one diagnosed before the age of 50 years and at least one first degree relative with ovarian cancer.

"If you do belong to such a family and are a first degree relative of the person with cancer you may be at slightly higher risk of developing ovarian cancer and require regular screening. If you are concerned about your risk of developing ovarian cancer, further information can be obtained from the OVACOME (a support group for ovarian cancer patients and their families, tel: 0207 380 9589), Cancer BACUP (an information service about cancer, tel: 0808-800-1234) or your GP."

Do I have to take part?

It is up to you to decide whether or not to take part. If you do decide to take part you will be asked to sign a consent form. If you decide to take part you are free to withdraw at any time and without giving a reason. This will not affect any medical care you receive.

What will happen to me if I take part?

If you agree to take part in this study please attend the appointment that you have been given in the attached letter. If you are unable to make this appointment, please phone to re-arrange to a more convenient time. At the appointment, you will be shown a video about the study and given the opportunity to ask any questions. If you decide to participate you will be asked to fill a datasheet and to sign a consent and data protection form. A blood sample will be taken. Your General Practitioner will be informed of your participation.

You will be given a further questionnaire to complete at home and return by post to assess the psychological implications of screening and the acceptability of the screening methods. This should not take more than 10 minutes of your time. A small number of women including those who have abnormal results on screening will be sent repeat questionnaires to complete at home in the course of the study.

Because we do not know if either of the screening methods are able to save lives from ovarian cancer we need to compare both methods of screening with a group of women who will not be screened. This will tell us whether screening can save lives and which method is most effective. This is called a randomised trial. The groups are selected by a computer and cannot be influenced by you or us. Of the 200,000 women who agree to participate half will be screened every year for 6 years and half will be in the control group and followed up with questionnaires without screening. Overall the study will take 10 years to complete. Of the 100,000 women who will be screened 50,000 will be screened with ultrasound and 50,000 with the CA 125 blood test.

Travel expenses (for visits due to the research study) cannot be guaranteed as they are not provided by the research funders. In the event of hardship, you may wish to discuss travel costs with the research team at your regional centre who will do their best to help you.

Control / Questionnaire Group

If you are allocated to the group not to be screened you will be asked to complete health related questionnaires 4 and 7 years after you are recruited. The questionnaires will be sent to your home for you to complete and you will not be required to attend any further appointments. We will request your GP to refer you to the same specialist at the regional centre as women who are allocated to the screened groups if at anytime you develop symptoms that raises the possibility of ovarian cancer. You will not be asked to have further blood tests or an ultrasound scan. The blood test taken at your registration visit will not be tested at this time but will be stored to be used at some stage in the future to assess potential tests for cancer. As these will be tests in the initial phases of being researched, the results will not be conclusive and therefore you will not be notified of the results. If you are allocated to this group your contribution is vital to the outcome of the study.

Blood test for CA125 group (multimodal group)

If you are allocated to the CA125 group the blood taken at your registration visit will be tested. Your potential risk of developing ovarian cancer (ROC) in the year following the blood test will be calculated. Your ROC will be recalculated after each blood test. The ROC calculation is looking for changes in your blood results over time. For this reason your recommended follow-up may vary during the course of the study but will be one of the following:

- If your ROC is within normal limits you will be asked to return for a further blood test in one year. Most women will fall into this group and be recalled annually throughout the study.
- If there is a slight change in the levels of your blood test your ROC will be classified as intermediate and you will be asked to return for a repeat blood test 3 months later.

- If your ROC is higher than would be expected (elevated) you will be sent an appointment for a further blood test and an ultrasound scan of your ovaries. It is important to note that most women who require an ultrasound scan will not have an abnormality of their ovary and will not require surgery. If the ultrasound scan does reveal an abnormality of the ovaries, you will be referred to a specialist at the regional centre for further tests and surgery to remove your ovaries. This will be arranged in consultation with yourself and your General Practitioner. Only 1 in 5 of the women who have an abnormal test and have surgery will have ovarian cancer. The others will usually have less serious conditions of the ovary.

Ultrasound group
If you are allocated to the ultrasound group you will be sent an appointment for a scan of your ovaries. If no abnormalities are detected on the scan you will be sent an appointment for a further scan in one year. If an abnormality is seen, you will be sent an appointment for a repeat scan in 6-8 weeks. This is because many of the abnormalities will disappear on their own and need no treatment. If the repeat scan is normal you will be recalled in one year. If the repeat scan shows that the changes seen in the first scan persist, you will be referred to a specialist at the regional centre for further tests and surgery to remove your ovaries. This will be arranged in consultation with yourself and your General Practitioner. Only 1 in 15 of the women who have an abnormality detected at ultrasound and have surgery will have ovarian cancer. The others will usually have non cancerous conditions of the ovary.

You will be notified by post of your recommended follow-up after every blood test or investigation.

Samples storage and future use
We will store a portion of all blood taken during the course of the trial. These samples will be stored indefinitely under the custodianship of University College London. Some of these stored samples will be used for future research studies. The focus of this research will be the early detection and treatment of disease. Researchers will have to get permission from the Ethics Committee before using any samples. If this happens, all the samples you donated will be used anonymously. As the samples will not have your name or identification, it will not be possible to trace them back to you or to inform you of the results. It is possible that collaborative research may include researchers working for commercial companies. If you are concerned about any future research please do discuss this with the UKCTOCS team. It is still possible for you to take part in the screening study if you do not wish the blood taken for analysis in this study to be used for future research.

Follow up
All study participants will be mailed a health questionnaire in years 3 and 7. In addition, the Office of National Statistics will be provided with the name, address and NHS number of all the study participants.

What do I have to do before the tests?
There is no special preparation for either the blood test or the ultrasound scan.

What is the procedure being tested?
Two screening procedures are being tested to find out whether ovarian cancer can be detected in the early stage.

The blood test for CA125
Most women who develop ovarian cancer have high levels of the protein called CA125 in their blood. The CA125 test is therefore currently used to diagnose ovarian cancer in women with symptoms and to monitor women after treatment. It has been shown that the CA125 test can be elevated in the early stage of many ovarian cancers. In addition, the research team believes that it may be possible to identify those women with early ovarian cancer where the CA125 blood test is not elevated by looking for changes i.e. increases in a woman's blood results over time.

Ultrasound scans
Ultrasound is currently widely used for diagnosing ovarian cancer in women with symptoms. It has been shown that the ultrasound test can be abnormal in the early stage of many ovarian cancers.

What are the possible disadvantages of taking part?

- Some women may have ovarian cancer that has not been identified by the test and thus be falsely reassured. If you develop any symptoms that you are concerned about, you should see your doctor as soon as possible.
- CA125 is not a test for all types of cancer. You should continue with all routine screening e.g. smear tests and breast screening, as advised by your GP surgery.
- Women taking part in the study have a 1 in 70 chance of being referred for surgery on the basis of abnormal screening results.

- Of every 1,000 women who undergo screening, 20-30 will have a positive screening test leading to surgery but only 2-3 of these women will actually have ovarian cancer. Most of the other women will be found to have other less serious abnormalities at surgery (e.g. ovarian cysts, endometriosis, scar tissue or infection). In a small number of women who undergo surgery no abnormality will be found at all.
- Not all women with ovarian cancer who are identified by the screening test will have early disease, the disease may be more advanced. The screening test may therefore not make a difference to the type of treatment a woman may receive or the outcome of the treatment.
- Having a screening test can create anxiety in some women.

What are the possible benefits of taking part?

- The only direct benefit of taking part for the women allocated to the questionnaire group is that we will request that they are referred to the specialist at the regional centre if their GP suspects that they may have an ovarian cancer. The participation of these women is however vital to the outcome of the study. Their participation will help decide whether either screening method is of benefit and whether a national screening programme should be implemented.
- If a woman allocated to the screened groups does have ovarian cancer, but no symptoms, there is at least an 80% chance that she would be identified by the test.
- If a woman allocated to the screened groups does not have cancer there is at least a 99.5% chance that the test will be negative and provide reassurance.

What if new information becomes available?

There is an independent Data Monitoring and Ethics committee that will assess the trial on an ongoing basis. If any information should become available that makes this study unethical, then this committee will recommend to the Trial Steering Committee which also has an independent chairman and non medical representatives that the study be stopped.

What if something goes wrong?

You will always be able to contact a research nurse or doctor at your regional centre to discuss your concerns and we will give you an emergency telephone number. We will take every care in the course of this trial. If through our negligence any harm to you results, you will be compensated. However, a claim may have to be pursued through legal action. Even if the harm is not our fault, the Trusts will consider any claim sympathetically. If you are not happy with any proposed compensation you may have to pursue your claim though legal action.

Will taking part in this study be kept confidential?

All information which is collected about you during the course of the research will be kept strictly confidential. Occasionally the research documentation and results will be looked at by the people funding the research programme to check that the study is being carried out properly. Any information about you which is viewed by people not directly related to the research team will have your name and address removed so that you cannot be recognised from it.

What will happen to the results of the research study?

The results will be reviewed by other medical professionals and published in the medical press. Results will probably only be available within a 12 year period. Should either screening method prove to be of benefit the results will be presented to the government as a case for a national screening programme to be implemented. You will be notified in writing that the study has been completed and of the outcome. Individuals will not be identified in any publications.

Who is organising and funding the research?

The study is being organised by the Gynaecological Cancer Research Centre at the Institute of Women's Health, University College London. It is being funded jointly by the Medical Research Council (MRC), Cancer Research UK and the NHS Research and Development.

For further information please write to:
UKCTOCS
Gynaecological Cancer Research Centre
Institute of Women's Health, UCL
1st Floor, Maple House
149 Tottenham Court Road, London, W1T 7DN

United Kingdom Collaborative Trial of Ovarian Cancer Screening (UKCTOCS)

Please complete this form in BLACK INK and in BLOCK CAPITALS. It will help us check that you are eligible for the study as well as collect some data regarding your risk of developing ovarian cancer. If the personal or GP details are incorrect please insert the correct details into the "Amended details" box.

Your Personal Details

Amended details

Your GP Details

Dr

NHS No. **D.o.B.** **AP** **ID**

Your Home Telephone No.

Your Work Telephone No.

ELIGIBILITY DETAILS **(use black ink and BLOCK CAPITALS or place a cross "X" in the appropriate boxes)**

1. When was your last period? **(dd/mm/yyyy)** ☐☐ / ☐☐ / ☐☐☐☐

2. Are you currently on Hormone Replacement Therapy (HRT)? Yes ☐ No ☐

   If Yes then when did you start taking HRT? **(dd/mm/yyyy)** ☐☐ / ☐☐ / ☐☐☐☐

3. Have you had both your ovaries removed? Yes ☐ No ☐

4. Have you ever had cancer diagnosed (except skin cancer)? Yes ☐ No ☐ Go to Q5

   If yes, what cancer was it?

   ☐ Ovary ☐ Breast ☐ Bowel ☐ Lung ☐ Other

   When was it diagnosed? **(dd/mm/yyyy)** ☐☐ / ☐☐ / ☐☐☐☐

5. Have you had any treatment for any cancer (including surgery, chemotherapy, radiotherapy) in the last 12 months(not including tamoxifen)? Yes ☐ No ☐

6. If any of the following relatives have had **OVARIAN CANCER** please write the number of affected relatives in the approprate box. Please enter 0 for no affected relatives. **(e.g. 0 Mother, 2 Sister, 0 Daughter).**

☐ Mother ☐ Daughter ☐ Sister ☐ Aunt ☐ GrandMother ☐ GrandDaughter

7. If any of the following relatives have had **BREAST CANCER** please write the number of affected relatives in the approprate box. Please enter 0 for no affected relatives. **(e.g. 0 Mother, 2 Sister, 1 Daughter).**

☐ Mother ☐ Daughter ☐ Sister ☐ Aunt ☐ GrandMother ☐ GrandDaughter

8. Are you currently taking part in any other ovarian cancer screening trial? Yes ☐ No ☐

If yes what is your study reference number? ☐☐☐☐☐☐☐☐☐☐

ADDITIONAL INFORMATION

ID ☐

9. Your height (cm) ☐☐☐ Your weight (kg) ☐☐☐

Or (in) ☐☐☐ Or (lb) ☐☐☐

10. Country of birth (please place an "X" as appropriate)

England ☐ Northern Ireland ☐ Scotland ☐ Irish Republic ☐ Wales ☐ Elsewhere ☐

11. Ethnic group, please place an "X" in the appropriate box. (If you are descended from more than one ethnic or racial group, please sellect the group you consider you belong to or choose "Any other ethnic origin")

☐ White ☐ Indian ☐ Pakistani ☐ Chinese ☐ Bangladeshi

☐ Black-African ☐ Black-Caribbean ☐ Black-other ☐ Any other ethnic origin

12. At what age did you first have your period? ☐☐

13. How many pregnancies have you had which ended before they reached 6 months (including miscarriages, ectopic pregnancies)? ☐☐

14. How many pregnancies have you had which lasted beyond 6 months (including all deliveries - both term and preterm)? ☐☐

15. Have you ever taken the oral contraceptive pill? Yes ☐ No ☐

If yes, how many years in total did you take the pill? Years ☐☐

16. Have you ever had a hysterectomy (removal of the womb)? Yes ☐ No ☐

17. Have you had a sterilisation operation (To block your tubes)? Yes ☐ No ☐

18. Have you ever had any treatment for infertility? Yes ☐ No ☐

GENERAL HEALTH QUESTIONNAIRE (GHQ-12)

Name: **Date:**

Please read this carefully.

We should like to know if you have had any medical complaints and how your health has been in general, over the last few weeks. Please answer ALL the questions simply by underlining the answer which you think most nearly applies to you. Remember that we want to know about the present and recent complaints, not those you have had in the past.

It is important that you try to answer ALL the questions.

Have you recently . . .

1.	been able to concentrate on whatever you're doing?	Better than usual	Same as usual	Less than usual	Much less than usual
2.	lost much sleep over worry?	Not at all	No more than usual	Rather more than usual	Much more than usual
3.	felt that you are playing a useful part in things?	More so than usual	Same as usual	Less useful than usual	Much less useful
4.	felt capable of making decisions about things?	More so than usual	Same as usual	Less so than usual	Much less than usual
5.	felt constantly under strain?	Not at all	No more than usual	Rather more than usual	Much more than usual
6.	felt you couldn't overcome your difficulties?	Not at all	No more than usual	Rather more than usual	Much more than usual
7.	been able to enjoy your normal day-to-day activities?	More so than usual	Same as usual	Less so than usual	Much less than usual
8.	been able to face up to your problems?	More so than usual	Same as usual	Less so than usual	Much less able
9.	been feeling unhappy and depressed?	Not at all	No more than usual	Rather more than usual	Much more than usual
10.	been losing confidence in yourself?	Not at all	No more than usual	Rather more than usual	Much more than usual
11.	been thinking of yourself as a worthless person?	Not at all	No more than usual	Rather more than usual	Much more than usual
12.	been feeling reasonably happy, all things considered?	More so than usual	About same as usual	Less so than usual	Much less than usual

GENERAL HEALTH QUESTIONNAIRE 12 © David Goldberg, 1978. Reproduced by permission of the Publishers, The NFER-NELSON Publishing Company Ltd., Darville House, 2 Oxford Road East, Windsor, Berkshire SL4 1DF, England. All rights reserved.

SELF-EVALUATION QUESTIONNAIRE
STAI Form Y-2

DIRECTIONS

A number of statements which people have used to describe themselves are given below. Read each statement and then circle the appropriate value to the right of the statement to indicate how you *generally* feel. There are no right or wrong answers. Do not spend too much time on any one statement but give the answer which seems to describe how you generally feel.

	ALMOST NEVER	SOMETIMES	OFTEN	ALMOST ALWAYS
21. I feel pleasant	1	2	3	4
22. I feel nervous and restless	1	2	3	4
23. I feel satisfied with myself	1	2	3	4
24. I wish I could be as happy as others seem to be	1	2	3	4
25. I feel like a failure	1	2	3	4
26. I feel rested	1	2	3	4
27. I am "cool, calm and collected"	1	2	3	4
28. I feel that difficulties are piling up so that I cannot overcome them	1	2	3	4
29. I worry too much over something that doesn't really matter	1	2	3	4
30. I am happy	1	2	3	4
31. I have disturbing thoughts	1	2	3	4
32. I lack self-confidence	1	2	3	4
33. I feel secure	1	2	3	4
34. I make decisions easily	1	2	3	4
35. I feel inadequate	1	2	3	4
36. I am content	1	2	3	4
37. Some unimportant thought runs through my mind and bothers me	1	2	3	4
38. I take disappointments so keenly that I can't put them out of my mind	1	2	3	4
39. I am a steady person	1	2	3	4
40. I get in a state of tension and turmoil as I think over my recent concerns and interests	1	2	3	4

© Copy right 1968, 1977 by Charles D. Spielberger. All rights reserved.

STRICTLY CONFIDENTIAL

SEXUAL ACTIVITY QUESTIONNAIRE
(© Fallowfield)

Although the following questions are sensitive and personal, they are important in determining how different tests and treatments affect this part of your life. Please be assured that your responses to these questions will remain confidential.

Section I

	Yes	No
**1. Are you currently married or having an intimate relationship with someone?**	☐	☐
**2. Have you changed your sexual partner in the last 6 months?**	☐	☐
**3. Do you engage in sexual activity with anyone at the moment?**	☐	☐
	**If 'Yes' please go to next page**	**If 'No' please answer remaining questions on this page**

Section II

I answered 'No' to question 3. I am not sexually active at the moment because:
(Please tick as many of these items as apply)

a) I do not have a partner at the moment ☐

b) I am too tired ☐

c) My partner is too tired ☐

d) I am not interested in sex ☐

e) My partner is not interested in sex ☐

f) I have a physical problem which makes sexual relations difficult or uncomfortable ☐

g) My partner has a physical problem which makes sexual relations difficult or uncomfortable ☐

h) Other reasons (please describe) ☐

PTO

Please complete this section if you are sexually active (i.e. you answered 'Yes' to question 3).

Please read each of the following questions carefully and tick the box that best indicates your sexual feelings and experiences during the past month.

Section III

During the past month:

	very much	somewhat	a little	not at all
1. Was ‘having sex’ an important part of your life this month?	☐	☐	☐	☐
2. Did you enjoy sexual activity this month ?	☐	☐	☐	☐
3. In general, were you too tired to have sex ?	☐	☐	☐	☐
4. Did you desire to have sex with your partner(s) this month?	☐	☐	☐	☐
5. During sexual relations, how frequently did you notice dryness of your vagina this month?	☐	☐	☐	☐
6. Did you feel pain or discomfort during penetration this month?	☐	☐	☐	☐
7. In general, did you feel satisfied after sexual activity this month?	☐	☐	☐	☐

	5 times or more	3-4 times	1-2 times	not at all
8. How often did you engage in sexual activity this month?	☐	☐	☐	☐

	much more	somewhat more	about the same	not at all
9. How did this frequency of sexual activity compare with what is usual for you?	☐	☐	☐	☐

	very much	somewhat	a little	not at all
10. Were you satisfied with the frequency of sexual activity this month?	☐	☐	☐	☐

Any other comments?

Thank you very much for answering these questions.

Questionnaire to assess risk perception

Please read the following statements and tick those you believe to be true about ovarian cancer

1. The chances of a woman getting ovarian cancer in her lifetime in this country are - (please tick one)
   - About 1 in 500 ☐
   - About 1 in 70 ☐
   - About 1 in 12 ☐

2. Most ovarian lumps turn out to be (please tick one)
   - Cysts ☐
   - Cancer ☐
   - Don't know ☐

3. The chances of an ovarian lump being cancer are highest in women aged - (please tick one)
   - 25-35 ☐
   - 35-45 ☐
   - 45-55 ☐
   - 55-65 ☐
   - over 65 ☐

4. A woman is more likely to develop ovarian cancer if she - (tick any one of these you think may apply)
   - has never been pregnant or had children ☐
   - did not breast feed ☐
   - took the pill ☐
   - has relatives with breast cancer ☐
   - has relatives with ovarian cancer ☐
   - is past the menopause ☐
   - has had breast cancer ☐
   - has had a benign cyst in the ovary ☐
   - has had an abnormal cervical smear ☐

5. What do you think is your lifetime risk of developing ovarian cancer?
   - 1 in 10 chance ☐
   - 1 in 100 chance ☐
   - 1 in 500 chance ☐

6. Have you spoken to other members of your family about the risk of ovarian cancer?

   ☐ **Yes** ☐ **No**

7. Do you think you are at higher risk of developing other cancers than ovarian cancer?

   ☐ **Yes** ☐ **No**

	strongly agree	agree a little	disagree a little	strongly disagree
1. If more women went for ovarian screening there would be fewer deaths from ovarian cancer	☐	☐	☐	☐
2. My health is too good at present even to consider thinking that I might get ovarian cancer	☐	☐	☐	☐
3. If a lump is found in your ovaries it is usually too late to do anything about it	☐	☐	☐	☐
4. Whenever I hear of a friend/relative or public figure getting ovarian cancer I realise I could get it too	☐	☐	☐	☐
5. If I look out for the symptoms of ovarian cancer I might find something sooner than if I go for screening	☐	☐	☐	☐
6. There are so many things that could happen to me that it is pointless to think about ovarian cancer	☐	☐	☐	☐
7. Even though it is a good idea, I find gynaecological examination an embarrassment	☐	☐	☐	☐
8. The older I get the more I think about the possibility of getting ovarian cancer	☐	☐	☐	☐
9. Coming for screening would/has only made me worry (unnecessarily) about ovarian cancer	☐	☐	☐	☐
10. If I was found to have ovarian cancer by screening the chances of it being cured are higher	☐	☐	☐	☐

Acceptability of screening

1. How did you feel when you realised you were randomised to screening?
(please tick box for each item)

	very	a little	not at all
worried	☐	☐	☐
irritated	☐	☐	☐
pleased	☐	☐	☐

2. Was all the information you were given clear and understandable?

very	fairly	not at all
☐	☐	☐

3. Had you heard about ovarian cancer screening from any of the following before receiving the invitation?
(please tick any that apply)

your doctor (GP) ☐
friends/family ☐
radio/TV ☐
magazines ☐
newspaper ☐

4. Before deciding to join the trial did you discuss the invitation with any of the following?
(please tick any that apply)

your doctor (GP) ☐
friends/family ☐
other ☐

5. Has anyone close to you ever had ovarian cancer?

Yes ☐ **No** ☐

If Yes: Who was this?

(please tick any that apply)

Mother ☐
Friend ☐
Sister ☐
Daughter ☐
Aunt ☐

6. Did you have any difficulties getting to the ovarian cancer screening centre, such as transportation
problems or getting time off work during the day?

Yes ☐ **No** ☐

Please give details

7. At the screening centre, did you feel
(please tick box for each item)

	very	fairly	not at all
relaxed	☐	☐	☐
anxious	☐	☐	☐

8. Were the staff at the screening centre
(please tick box for each item)

	very	fairly	not at all
pleasant	☐	☐	☐
unhelpful	☐	☐	☐

9. How long were you required to wait?

less than 20 min	20-60 min	over an hour
☐	☐	☐

10. Do you intend to come again in a year's time if you are randomly selected to do so?

yes	unsure	no
☐	☐	☐

Please state whether you have had an internal (transvaginal) ultrasound scan and/or a blood test for ovarian cancer performed, and if so your experience of either of them.

Transvaginal Ultrasound Scan

11. Have you ever had an internal (transvaginal) scan (please tick)

Yes ☐ **No** ☐ **Don't know** ☐

If yes please state date of last test ______________

12. Please rate your discomfort/pain during the scan

severe	moderate	mild	none
☐	☐	☐	☐

13. Please rate your embarrassment during the scan

severe	moderate	mild	none
☐	☐	☐	☐

14. Please rate the worry caused by the scan itself

severe	moderate	mild	none
☐	☐	☐	☐

15. Please rate your impression of the time taken to perform the scan

too long	just right	too short
☐	☐	☐

16. Please rate how intrusive you felt the scan to be

very	moderately	not at all
☐	☐	☐

<u>Blood test for Ovarian Cancer</u>

17. Have you ever had a blood test for ovarian cancer (please tick)

Yes ☐ **No** ☐ **Don't know** ☐

If yes please state date of last test ______________

18. Please rate your discomfort/pain during the blood test

severe	moderate	mild	none
☐	☐	☐	☐

19. Please rate your embarrassment during the blood test

severe	moderate	mild	none
☐	☐	☐	☐

20. Please rate the worry caused by the blood test itself

severe	moderate	mild	none
☐	☐	☐	☐

21. Please rate your impression of the time taken to perform the blood test

too long	just right	too short
☐	☐	☐

22. Please rate how intrusive you felt the blood test to be

very	moderately	not at all
☐	☐	☐

23. Would you recommend ovarian cancer screening to your friends?

yes	unsure	no
☐	☐	☐

Any other comments?

Thank you for your help.

FACT-O (Version 4)

Below is a list of statements that other people with your illness have said are important.
By circling one (1) number per line, please indicate how true each statement has been for you during the past 7 days.

	PHYSICAL WELL-BEING	not at all	a little bit	some-what	quite a bit	very much
1	I have a lack of energy	0	1	2	3	4
2	I have nausea	0	1	2	3	4
3	Because of my physical condition, I have trouble meeting the needs of my family	0	1	2	3	4
4	I have pain	0	1	2	3	4
5	I am bothered by side effects of treatment	0	1	2	3	4
6	I feel ill	0	1	2	3	4
7	I am forced to spend time in bed	0	1	2	3	4

	SOCIAL/FAMILY WELL-BEING	not at all	a little bit	some-what	quite a bit	very much
8	I feel close to my friends	0	1	2	3	4
9	I get emotional support from my family	0	1	2	3	4
10	I get support from my friends	0	1	2	3	4
11	My family has accepted my illness	0	1	2	3	4
12	I am satisfied with family communication about my illness	0	1	2	3	4
13	I feel close to my partner (or the person who is my0 main support)	1	2	3	4	
	Regardless of your current level of sexual activity, please answer the following question. If you prefer not to answer it, please check this box ☐ and go on to the next section.					
14	I am satisfied with my sex life	0	1	2	3	4

FACT-O (Version 4)

By circling one (1) number per line, please indicate how true each statement has been for you during the past 7 days.

	EMOTIONAL WELL-BEING	not at all	a little bit	some-what	quite a bit	very much
15	I feel sad	0	1	2	3	4
16	I am satisfied with how I am coping with my illness	0	1	2	3	4
17	I am losing hope in the fight against my illness	0	1	2	3	4
18	I feel nervous	0	1	2	3	4
19	I worry about dying	0	1	2	3	4
20	I worry that my condition will get worse	0	1	2	3	4

	FUNCTIONAL WELL-BEING	not at all	a little bit	some-what	quite a bit	very much
21	I am able to work (include work at home)	0	1	2	3	4
22	My work (include work at home) is fulfilling	0	1	2	3	4
23	I am able to enjoy life	0	1	2	3	4
24	I have accepted my illness	0	1	2	3	4
25	I am sleeping well	0	1	2	3	4
26	I am enjoying the things I usually do for fun	0	1	2	3	4
27	I am content with quality of my life right now	0	1	2	3	4

FACT-O (Version 4)

By circling one (1) number per line, please indicate how true each statement has been for you during the past 7 days.

ADDITIONAL CONCERNS	not at all	a little bit	some-what	quite a bit	very much
28 I have a swelling in my stomach area	0	1	2	3	4
29 I am losing weight	0	1	2	3	4
30 I have control of my bowels	0	1	2	3	4
31 I have been vomiting	0	1	2	3	4
32 I am bothered by hair loss	0	1	2	3	4
33 I have a good appetite	0	1	2	3	4
34 I like the appearance of my body	0	1	2	3	4
35 I am able to get around by myself	0	1	2	3	4
36 I am able to feel like a woman	0	1	2	3	4
37 I have cramps in my stomach area	0	1	2	3	4
38 I am interested in sex	0	1	2	3	4

UKCTOCS
Gynaecological Cancer Research Centre
Institute of Women's Health, UCL
1st Floor, Maple House
149 Tottenham Court Road, London W1T 7DN

Centre Number:
Study Number: ISRCTN Number 22488978
Patient identification number for this trial:

CONSENT FORM

Thank you for reading the information about our research project. If you would like to take part, please read and sign this form

Title of Project: **United Kingdom Collaborative Trial of Ovarian Cancer Screening (UKCTOCS)**

Name of Local Researcher:

Please initial box

1. I confirm I have read and understand the information sheet (Version 7, March 2004) for the above study and have had the opportunity to ask questions.	
2. I understand that my participation is voluntary and I agree to give samples of blood for research. I understand how the sample will be collected and that I am free to withdraw my approval for use of the sample at anytime, without giving any reason and without my medical care or legal rights being affected.	
3. I give permission for individuals from the UKCTOCS research team to access my medical notes for information relevant to the research. I understand that regulatory authorities may also access this information.	
4. I understand any information I give will be treated in confidence.	
5. I understand that I will not benefit financially if this research leads to the development of a new treatment or medical test.	
6. I agree that the sample I have given and the information gathered can be stored by the custodians, University College London for possible use in future projects, as described in the information sheet. I understand that this material may be used for future research projects and that researchers other than those named above may carry out some of these projects. This may include researchers from commercial companies. I understand that my right to confidentiality will be protected at all times.	

______________________	____________	________________
Name of Volunteer	Date	Signature
______________________	____________	________________
Name of person taking consent (if different from researcher)	Date	Signature
______________________	____________	________________
Researcher	Date	Signature

(1 for patient; 1 for researcher; 1 to be kept with hospital notes)

UKCTOCS
CRC Psychosocial Oncology Group
TCMR School of Biological Sciences
University of Sussex
Falmer
Brighton BN1 9RX

UKCTOCS
Gynaecology Cancer Research Unit
Department of Obstetrics and Gynaecology
St Bartholomew's and the Royal London School of Medicine and Dentistry
West Smithfield London EC1A 7BE

UKCTOCS
United Kingdom Collaborative Trial of Ovarian Cancer Screening

CONSENT TO THE PSYCHOSOCIAL RESEARCH STUDY

Please tick and initial

I have read the Patient Information Sheet ☐

I have had an opportunity to ask questions and discuss this study ☐

I have received satisfactory answers to all my questions ☐

I have received enough information about the study ☐

The study was explained to me by: ______________________________

I understand that I am free to leave the study at any time ☐

- **you do not have to give a reason for leaving**
- **this will not affect any future medical care**

I agree to take part in the study ☐

I agree for my answers to be used in the research ☐

I agree for my information to be archived and made available for future studies within the research team? ☐

Signature ______________________________

NAME IN BLOCK CAPITALS ______________________________

Signature of witness ______________________________

Name in block capitals ______________________________

Date ______________________________

Version 2, January 2001

Funded by the Medical Research Council

QMW Data Notification Registration Number: Z4764785
University of Sussex Data Notification Registration Number: A0859013
ISRCTN 22488978

DATA PROTECTION ACT 1998

In accordance with the above Act we have a responsibility to inform you of the following:

- All data pertaining to your participation in the United Kingdom Collaborative Trial of Ovarian Cancer Screening (UKCTOCS) will be stored under the auspices of University College London data registration, Registration number Z6364106.
- This data will be used solely for research purposes as detailed in the UKCTOCS information leaflet and as discussed at registration for the research trial.
- As outlined in the UKCTOCS information leaflet, data obtained as a result of your participation in UKCTOCS may be used for other research projects, for example investigations into heart disease or other cancers. In this event all personal details will be removed prior to the data being analysed by researchers so that you cannot be identified, that is your name, address and GP details will not be made available.
- The data will be stored for a minimum of 15 years after the end of the trial. At any stage during this time the records may be audited to validate the trial data.
- Routine follow-up will be regenerated and attendance monitored by the trial database. All abnormal results and referrals for surgical intervention will be performed manually by the trial medical team. The database will be audited regularly to ensure that all follow-up is appropriate and prompt.
- If during the course of the trial you have any queries regarding the storage of your data you should contact the Safety Officer, UKCTOCS, Gynaecological Cancer Research Centre, Institute of Women's Health, UCL, 1st Floor, Maple House, 149 Tottenham Court Road, London W1T 7DN.

I have read the above information and have had the opportunity to discuss the impact of the Data Protection Act 1998 on my participation in UKCTOCS. I am happy for my details to be managed in this manner.

Signature: Witness:

Date: Date:

The Gynaecological Cancer Research Centre, based at University College London is co-ordinating the UKCTOCS trial. For the past 15 years much of the pioneering work to come out of this unit has been made possible as a direct result of grants provided by the charity "The Gynaecology Cancer Research Fund" known as The Eve Appeal, Registered charity number 1091708. The Gynaecology Cancer Research Fund undertakes fundraising events throughout the year in order to raise funds for research.

Please place a tick in the appropriate box.

I wish to receive information about the fund raising work of the Gynaecology Cancer Research Fund. ☐

I do not wish to receive information about the fund raising work of the Gynaecology Cancer Research Fund. ☐

Please be assured that your decision will not affect in any way your participation in UKCTOCS.

Signature: Witness:

Date: Date:

0175097515

UKCTOCS United Kingdom Collaborative Trial of Ovarian Cancer Screening

Volunteer Ref:

U K C T O C S

Private and Confidential

For the information that you supply to be useful to our ovarian cancer screening study, it is important that you complete all of the questions in "bold"

X Please use a BLACK pen and place a cross INSIDE the box X

Please return both sheets using the Free Post envelope supplied

Follow up questionnaire

We would be very grateful if you could answer the following questions. If you are not sure about exact details/dates an approximate answer is better than none. If there are any relevant details you wish to include, please use an additional sheet. Please use a BLACK biro or ink pen.

General questions about you

What qualification(s) do you have from school, college or the equivalent?

(please place a cross inside the most appropriate box(es))

- ☐ "O" level or equivalent
- ☐ Nursing or teaching
- ☐ "A" level or equivalent
- ☐ College/ university degree (or equivalent)
- ☐ Clerical or commercial qualification (e.g. secretarial, hairdressing etc)
- ☐ None of these

Approximately how much alcohol on average do you drink each week? (One drink = a glass of wine, half a pint of lager or cider, a measure of spirits). Average number of drinks of alcohol each week :

☐ None ☐ Less than 1 ☐ 1-3 ☐ 4-6 ☐ 7-10 ☐ 11-15 ☐ 16-20 ☐ 21+

Have you ever been a smoker? ☐ Yes ☐ No

If you answered yes to the above please answer the following questions:

How many years in total have you smoked for? ☐☐

During those years how many cigarettes on average did you smoke per day? ☐☐

What was your skirt size when you were in your early twenties?

☐ 6 ☐ 8 ☐ 10 ☐ 12 ☐ 14 ☐ 16 ☐ 18 ☐ 20 ☐ 22 ☐ 24 ☐ 26 ☐ 28 ☐ 30

What is your skirt size now?

☐ 6 ☐ 8 ☐ 10 ☐ 12 ☐ 14 ☐ 16 ☐ 18 ☐ 20 ☐ 22 ☐ 24 ☐ 26 ☐ 28 ☐ 30

Are you currently taking HRT? ☐ No ☐ Yes

Have you used any of the following to relieve menopausal symptoms? ☐ Yes ☐ No

- ☐ Herbal remedies e.g. Black cohosh
- ☐ Homeopathic remedies
- ☐ Phytoestrogens or soy products
- ☐ Aromatherapy, reflexology or acupuncture
- ☐ Vitamins e.g. Menopace, vitamin E
- ☐ Life style changes e.g. relaxation, exercise
- ☐ Other medical treatments e.g. Venlafaxine, Megace

We are interested to know more about how women deal with the menopause. If you are 50-60 years old would you be willing to complete a survey? ☐ Yes ☐ No

Questions about your outlook on life

Using the scale below, please indicate the extent to which you agree with each item:

1. The future seems to me to be hopeful, and I believe that things are changing for the better.

☐ Absolutely agree ☐ Somewhat agree ☐ Cannot say ☐ Somewhat disagree ☐ Absolutely disagree

2. I feel that it is possible to reach the goals I would like to strive for.

☐ Absolutely agree ☐ Somewhat agree ☐ Cannot say ☐ Somewhat disagree ☐ Absolutely disagree

Questions about your Health

Do you have/are you being treated for any of the following conditions (multiple boxes can be crossed)?

☐ High blood pressure ☐ Diabetes ☐ Stroke

☐ Heart disease e.g. heart attack, angina ☐ Rheumatoid arthritis ☐ Osteoporosis

☐ High blood cholesterol ☐ Osteoarthritis

☐ I have not been treated for any of the above conditions

Since joining UKCTOCS have you had any of the following operations? ☐ Yes ☐ No

If "yes" please enter the details (multiple boxes can be filled; please fill even if you had them as part of the trial)

Operation to look at your ovaries -either by incision or keyhole (laparoscopy)

Year of operation: ____________ Hospital No.: ____________

Hospital at which operation took place: ____________

____

Name of Consultant: ____________

Hysterectomy / Removal of womb

Year of operation: ____________ Hospital No.: ____________

Hospital at which operation took place: ____________

____

Name of Consultant: ____________

Removal of ovaries (please tick one box) ☐ Right Ovary ☐ Left Ovary ☐ Both ovaries

Year of operation: ____________ Hospital No.: ____________

Hospital at which operation took place: ____________

____

Name of Consultant: ____________

Hysteroscopy / D&C / Scrape of your womb / Operation to look at your womb

Year of operation: ____________ Hospital No.: ____________

Hospital at which operation took place: ____________

____

Name of Consultant: ____________

Were there any complications resulting from any of the above procedures? ☐ Yes ☐ No

If yes, please give details using an extra sheet of paper if necessary.

____

____

Breast biopsy or other surgery involving the breast

Year of operation: ____________ Hospital No.: ____________

Hospital at which operation took place: ____________

____

____

Name of Consultant: ____________

Any other operation of any kind. Please describe:

Since joining UKCTOCS **have you been diagnosed with any cancer? Please tell us about this(ese) cancer(s)**

- ☐ Ovarian cancer
- ☐ Breast cancer
- ☐ Cervical cancer
- ☐ Endometrial/uterus/womb cancer
- ☐ Bowel/colorectal cancer
- ☐ Gastric/stomach cancer
- ☐ Pancreatic cancer
- ☐ Kidney cancer
- ☐ Lung cancer
- ☐ Vulval/vaginal cancer
- ☐ BCC/rodent/skin cancer
- ☐ Other cancer
- ☐ I have not been diagnosed with any cancers

Type of cancer: ____________

Year of operation: ____________ Hospital No.: ____________

Hospital at which operation took place: ____________

____

____

Name of Consultant: ____________

Type of cancer: ____________

Year of operation: ____________ Hospital No.: ____________

Hospital at which operation took place: ____________

____

____

Name of Consultant: ____________

Once you have completed this questionnaire please sign and date below, then return it in the Free Post envelope. Thanks you.

Name (please print): Date: ☐☐ / ☐☐ / ☐☐☐☐

Signature: (dd/mm/yyyy)

QUOX 1 ☐ 2 ☐ 3 ☐ **Office use only** **QU Consent**

Yes ☐ **No** ☐

■ ■ The following sections should ONLY be completed by those volunteers who are in the CONTROL group

■ **Since joining UKCTOCS have you had an ultrasound scan of your ovaries?** ☐ **Yes** ☐ **No**

If "yes" why was this performed?

☐ GP request

☐ Hospital Doctor request

☐ Your own request

☐ Other reason ____________________

Year of scan: __________ Hospital No.: __________

Hospital at which operation took place: __________

____

____

Name of Consultant: __________

■ **Since joining UKCTOCS have you had a blood test for CA125?** ☐ **Yes** ☐ **No**

(CA125 is a substance, which is released at higher levels into the blood in women with ovarian cancer. The test is carried out if doctors suspect that a woman may have ovarian cancer)

If "yes" why was this performed?

☐ GP request

☐ Hospital Doctor request

☐ Your own request

☐ Other reason ____________________

Year of CA125 test: __________ Hospital No.: __________

Hospital at which operation took place: __________

____

____

Name of Consultant: __________

Thank you for taking the time to complete this questionnaire. The information that you supply is of great importance to the success of the trial.

Regards

UKCTOCS Team

UKCTOCS
United Kingdom Collaborative Trial of Ovarian Cancer Screening

PELVIC ULTRASOUND SCAN RESULTS

First Name ________________________ **Surname** ________________________ **Volunteer Ref No** ______________________

Date ___________/__________/_____________ **Ultrasonographer** ____

Please tick the relevant box

Mode of scan ☐ TRANSABDOMINAL ☐ TRANSVAGINAL ☐ BOTH **Latex allergy** ☐ YES ☐ NO

Period in last year ☐ YES ☐ NO **Date of last period (if within the last year)** _____/__________/__________

Type of HRT if used ☐ ESTROGEN ☐ PROGESTOGEN ☐ COMBINED CYCLICAL (CONVENTIONAL)
☐ COMBINED CONTINUOUS (NO BLEED) ☐ TIBOLONE ☐ OTHER

Hysterectomy ☐ **Previous oophorectomy** ☐ NONE ☐ LEFT OOPHORECTOMY ☐ RIGHT OOOPHORECTOMY

DETAILS OF OVARIAN SCAN

	LEFT OVARY / ADNEXA	RIGHT OVARY / ADNEXA
**VISUALISATION** **MUST BE COMPLETED**	☐ SEEN<br>☐ NOT SEEN / GOOD VIEW<br>☐ NOT SEEN / POOR VIEW<br>☐ NOT SEEN / PREVIOUS OOPHERECTOMY	☐ SEEN<br>☐ NOT SEEN / GOOD VIEW<br>☐ NOT SEEN / POOR VIEW<br>☐ NOT SEEN/ PREVIOUS OOPHERECTOMY
**If ovary not seen, reason**	☐ BOWEL ☐ FIBROIDS<br>☐ PELVIC VARICOSITIES<br>☐ OTHER	☐ BOWEL ☐ FIBROIDS<br>☐ PELVIC VARICOSITIES<br>☐ OTHER
**OVARIAN DIMENSIONS**	mm mm mm	mm mm mm
**Morphology** **MUST BE COMPLETED IF OVARY SEEN** (If COMPLEX please write description of findings in abnormalities notes box & complete other side of this form. Also enter reference number at top of page)	☐ NORMAL<br>☐ SIMPLE CYST<br>☐ COMPLEX MORPHOLOGY	☐ NORMAL<br>☐ SIMPLE CYST<br>☐ COMPLEX MORPHOLOGY
	**If midline mass, please enter under left or right adnexa and describe below**<br>**If longstanding UNCHANGED complex morphology which has been previously investigated on UKCTOCS screening and is being managed conservatively, please fax form to Susan Davies on 0207 380 6929 for data entry**	
**Number of Cysts** **(more than 1 cyst = complex morphology)**		
**Ovary mobile?**	☐ YES ☐ NO	☐ YES ☐ NO

Max double endometrial thickness ____________ **mms** **Fluid POD or ascites (max vertical diameter)** _____________ **mms**

Details of abnormalities: **Referred in view of incidental findings** ☐ YES ☐ NO

Type of Image Record ☐ NONE ☐ DISK ☐ PHOTO ONLY ☐ BOTH DISK AND PHOTO

***Result Classification /Recommended Action Must Be Completed At Time Of Scan**

☐ NORMAL hence ROUTINE SCREENING ☐ UNSATISFACTORY hence REPEAT LEVEL 1 SCAN

☐ ABNORMAL therefore LEVEL 2 / SURGERY **(If any other option required, contact Susan Davies on 0207 380 6913)**

- **As defined by protocol.**

Entered Signature Date

Checked Signature Date

October 2005

UKCTOCS
United Kingdom Collaborative Trial of Ovarian Cancer Screening

Signature____ Date ____________________________

DETAILS OF ANY OVARIAN / ADNEXAL LESION DETECTED **VOLUNTEER REF (Please enter)**____________________

	LEFT OVARY/ADNEXA	RIGHT OVARY/ADNEXA
**Cyst dimensions**	mm mm mm	mm mm mm
**Cyst wall thickness**	mm	mm
**Cyst wall structure**	☐ SMOOTH ☐ IRREGULAR	☐ SMOOTH ☐ IRREGULAR
**Fluid in cyst**	☐ANECHOIC ☐ RANDOM ECHOGENICITY<br>☐UNIFORM ECHOGENICITY	☐ANECHOIC ☐ RANDOM ECHOGENICITY<br>☐UNIFORM ECHOGENICITY
**Cyst structures**	☐SEPTAE ☐ PAPILLATIONS	☐SEPTAE ☐ PAPILLATIONS
**Maximum septa thickness**	mm	mm
**Size of largest papillation**	mm	mm
**Solid areas**	☐ Yes ☐ No	☐ Yes ☐ No
**Overall impression of lesion**<br>(Classification using International Ovarian Tumour Analysis criteria)<br>**MUST BE COMPLETED IN THE PRESENCE OF AN OVARIAN LESION**	☐ Unilocular cvst<br>☐ Unilocular solid<br>☐ Multilocular cvst<br>☐ Multilocular solid<br>☐ Solid	☐ Unilocular cvst<br>☐ Unilocular solid<br>☐ Multilocular cvst<br>☐ Multilocular solid<br>☐ Solid

DOPPLER STUDY OF ABNORMAL AREA

**Doppler performed**	☐ YES ☐ NO
**Presence of colour signal**	☐ YES ☐ NO
**Location of colour signal**	☐ SEPTAE ☐ WALL<br>☐ SOLID AREA ☐ PAPILLATIONS<br>☐ OTHER
**Lowest RI measured**	
**Lowest PI measured**	
**Peak systolic velocity**	

Findings suggestive of ☐ DERMOID CYST ☐ ENDOMETRIOTIC CYST

Were scan images reviewed at CC? ☐ YES ☐ NO

Person at CC with whom results discussed ____

Suggested management ☐ AS RECOMMENDED ☐ OTHER **(if other, please enter details in notes)**

NOTES

United Kingdom Collaborative Trial of Ovarian Cancer Screening

UKCTOCS

Clinical Assessment Form

First Name______________________**Surname**_________________________**Volunteer RefNo**________________

Date _______/______/________ **Hospital**____

Consultant ____________________ **Name of Person seeing the patient** ___________________________

Grade of person assessing the patient

Consultant gynaecologist	Consultant gynaecological oncologist
Specialist Registrar (Obs and Gynae)	Specialist Registrar (Gynae Oncology)
Non-consultant gynaecological staff	Senior House Officer (Obs and Gynae)
General Surgeon	Other

Does the lady have any symptoms YES NO (Please circle if the volunteer has any symptoms)

**General health**	Lethargy	Malaise				
**Breasts**	Lump	Pain	Discharge	Skin problem		
**Gastro-intestinal tract**	Poor appetite	Constipation	Nausea	Bloating	Rectal bleeding	
	Diarrhoea	Malena	Vomiting	Dyspepsia	Pain	
**Urinary tract**	Urgency	Frequency	Dysuria	Nocturia	Haematuria	Incontinence
**Genital tract**	Abnormal vaginal bleeding		Dyspareunia	Post-coital bleeding		PV Discharge
**Other symptoms**						
**Overall duration of symptoms (Months)**						

Does the volunteer have any other illness that may contribute to her symptoms/findings? YES NO

If so, please provide details

Does the volunteer have any of the following conditions that may account for an elevated CA125 level?

**Polyarteritis nodosa**	**Rheumatoid arthritis**	**Osteoarthritis**	**Sjorgrens syndrome**	**Pancreatitis**	**Hepatitis**	**Colitis**
**Cirrhosis**	**Other arthritis**	**Sarcoid**	**SLE**	**Renal disease**	**Endometriosis**	**Diverticulitis**
**Pericarditis**	**Other -**					

Examination

SITE	NOT DONE	NORMAL	ABNORMAL
BREAST			
ABDOMEN			
PV			
RECTAL			

If abnormal, please specify abnormality and any other comments

Please fax and post to
Ms Susan Davies Fax No 0207 380 6929
Gynaecological Cancer Research Centre, Institute of Women's Health, UCL, London W1T 7DN

Clinical Assessment Form

Clinical impression (please tick)

Probably Benign gynaecological disease	
Probably Ovarian Malignancy	
Uncertain	
Other Gynaecological Malignancy	
Other Non gynaecological Malignancy	
Other	

Further investigations (please tick)

Test	Ordered	Abnormal
**USS/Doppler**		
**MRI**		
**CT**		
**Radio-immuno-scan**		
**CA125**		

Details of any abnormalities detected on investigation

Management decision

Surgery
Conservative management and follow up
Return to annual screening
Other

(If Volunteer is to have surgery, please fill section A; If volunteer is to be taken off protocol, please fill sectionB).

SECTION A – FOLLOWING PROTOCOL

Date Booked for Surgery _______/_______/_______ **Placed on W/L for surgery** YES NO

Proposed Surgery

SECTION B – OFF PROTOCOL **Rationale for Conservative management**

Details of follow up plan

Date of next appointment _______/______/________

Please tick if any 'off protocol' TVS or CA125 needs to be arranged by the UKCTOCS team

	To be arranged by UKCTOCS team	Interval from appt date
**CA125**		
**TVS**		

Signature: **Date:**

Please fax and post to
Ms Susan Davies Fax No 0207 380 6929
Gynaecological Cancer Research Centre, Institute of Women's Health, UCL, London W1T 7DN *Version 1, July 2002*

United Kingdom Collaborative Trial of Ovarian Cancer Screening

SURGERY RESULTS

First Name ________________________ **Surname** ___________________ **Volunteer Ref No** _________________

Hospital ___________________________ **Consultant** _________________

Date/time surgery started ___/___/___ ____: ____ **Date/time surgery ended** ___/___/___ ____: ____

Surgeon ____

Please tick the relevant box

Grade of surgeon
- ☐ Consultant Gynaecologist/Oncologist
- ☐ Consultant Gynaecologist
- ☐ Specialist Registrar *(Gynae Oncology)*
- ☐ Specialist Registrar *(Obs and Gynae)*
- ☐ Senior House Officer *(Obs and Gynae)*
- ☐ General Surgeon
- ☐ Non – consultant gynaecological staff
- ☐ Other

Indication *Reason why surgery took place*
- ☐ Incidental findings
- ☐ Screen Positive
- ☐ False Negative
- ☐ Unrelated

Abdominal Incisions
- ☐ Vertical
- ☐ Transverse
- ☐ Other

Procedure carried out
- ☐ Laparotomy
- ☐ Operative laparoscopy
- ☐ Hysteroscopy/D&C/EUA
- ☐ Other
- ☐ Diagnostic laparoscopy
- ☐ Diagonistic laparoscopy/laparotomy
- ☐ Vaginal surgery

Oopherectomy type *if undertaken*
- ☐ Not Sampled
- ☐ Left oopherectomy
- ☐ Right oopherectomy
- ☐ Bilateral oopherectomy

Hysterectomy type *if undertaken*
- ☐ Total abdominal
- ☐ Radical abdominal
- ☐ LAVH
- ☐ Other
- ☐ Not Sampled
- ☐ Subtotal abdominal
- ☐ LV
- ☐ Vaginal

Omentectomy type
- ☐ Not Sampled
- ☐ Omental biopsy
- ☐ Partial omentectomy
- ☐ Other

OvCyst Rupture *during surgery*
- ☐ N/A
- ☐ No
- ☐ Yes

Pelvic lymph node dissection *if undertaken*
- ☐ Not Sampled
- ☐ Pelvic lymph node sampling
- ☐ Left pelvic lymphadenectomy
- ☐ Right pelvic lymphadenectomy
- ☐ Bilateral pelvic lymphadenectomy

Paraortic Node Dissection *if undertaken*
- ☐ Not Sampled
- ☐ Para-aortic lymph node sampling
- ☐ Left para-aortic lymphadenectomy
- ☐ Right para-aortic lymphadenectomy
- ☐ Bilateral para-aortic lymphadenectomy

**Excision colon**	☐ YES ☐ NO	**Excision small bowel**	☐ YES ☐ NO
**Appendectomy**	☐ YES ☐ NO	**Excision sigmoid/rectum**	☐ YES ☐ NO
**Peritoneal washing taken**	☐ YES ☐ NO	**Excision spleen**	☐ YES ☐ NO
**Ascites sent for cytology**	☐ YES ☐ NO	**Estimated blood loss (ml):**	______

Any other Procedure undertaken
- ☐ Ovarian Cystectomy
- ☐ Myomectomy
- ☐ Adhesiolysis
- ☐ Curettage/endometrail biopsy
- ☐ Ultrasound guided aspirations of cyst
- ☐ Hysteroscopy
- ☐ Laparoscopy
- ☐ Other

Any other relevant notes

Signature of person filling the form ______________________ **Date signed** ___/___/___

SURGERY RESULTS

PRE-OPERATIVE EVENTS

No of GP visits prior to surgery ______________ **No of visits to outpatient clinic prior to surgery** ______________

Date of admission ___/___/___

Type of ward: ☐ **Gynaecology** ☐ **ITU** ☐ **General** ☐ **Other – details below**

Pre-operative investigations

Imaging

**Imaging carried out**	☐ YES ☐ NO	**Chest X-Ray**	☐ YES ☐ NO
**ECG**	☐ YES ☐ NO	**USS or Doppler on NHS**	☐ YES ☐ NO
**MRI**	☐ YES ☐ NO	**Radio immunoscintigraphy**	☐ YES ☐ NO
**CT Scan**	☐ YES ☐ NO	**Other imaging – details below**	☐ YES

No of tests

**No of venesections prior to surgery**	☐	**No of full blood counts prior to surgery**	☐
**No of liver function tests prior to surgery**	☐	**No of CA125 blood tests prior to surgery**	☐
**No of urea/electrolyte tests prior to surgery**	☐	**other blood test carried out prior to surgery– details below**	

POST-OPERATIVE EVENTS

Post-operative investigations

No of tests

**No of full blood counts post surgery**	☐	**No of liver function tests post surgery**	☐
**No of urea/electrolyte tests post surgery**	☐	**No of CA125 blood tests post surgery**	☐

Other blood test carried out post surgery– details below

Number of transfusions during admission

**Total no units of blood transfused**	☐	**Total no units of FFP transfused**	☐
**Total no units of platelets transfused**	☐	**Total no units of other transfused**	☐

Imaging

**ECG**	☐ YES ☐ NO	**Chest X-ray**	☐ YES ☐ No	**Radio Immunoscintingraphy**	☐ YES ☐ NO
**MRI**	☐ YES ☐ NO	**CT Scan**	☐ YES ☐ NO	**Ultrasound Scan**	☐ YES ☐ NO

Other Imaging

Any other relevant issues:

Length of stay in ITU/HDU (hrs) ____________ **Date of discharge** ___/___/___

SURGERY COMPLICATIONS

Intra-operative YES ☐ NO ☐ Post-operative YES ☐ NO ☐

(If yes to above please fill in Surgery Complications Form)

Signature of person filling the form _______________________ **Date signed** ___/___/___

SURGERY COMPLICATIONS

(to be completed in the event of a complication)

Intra-operation complications: ☐ YES ☐ NO

Excessive haemorrhage ☐ YES ☐ NO **Large Vessels Injury** ☐ YES ☐ NO

Ureteric Injury ☐ YES ☐ NO **Bowel Injury** ☐ YES ☐ NO

Other-Details below

Post operative complications: ☐ YES ☐ NO

Haemorrahage
- ☐ Primary
- ☐ Secondary
- ☐ Other

Thromboembolic event
- ☐ Deep vein thrombosis
- ☐ Pelvic vein thrombosis
- ☐ Pulmonary embolish
- ☐ Other

Vaginal vault haematoma ☐ YES ☐ NO

Wound defect ☐ YES ☐ NO

Urinary infections ☐ YES ☐ NO

Wound infections ☐ YES ☐ NO

Other complications (within 30 days of surgery)

Notes regarding complication

Signature of person filling the form ________________________ **Date signed** ___/___/___

SURGERY COMPLICATIONS

RE-ADMISSIONS AFTER PRIMARY SURGERY

Re-Admission ☐ **First** ☐ Second ☐ Third

Admission Date: ___/___/___ **Discharge Date:** ___/___/___

Reasons for patients re-admission

☐ Wound rupture
☐ Vaginal vault haematoma
☐ Wound infection
☐ Deep vein thrombosis
☐ Other - enter details
☐ Pulmonary embolism
☐ Bowel obstruction
☐ Urinary tract fistula
☐ Bowel fistula

Total number of Re-admissions: ☐

FURTHER SURGERY

Further surgery ☐ First ☐ Second ☐ Third

Procedure undertaken at re-admission

☐ Secondary suturing
☐ Evacuation of haematoma
☐ Laparotomy
☐ Revision of stoma
☐ Other

Other procedure ___________________________

Other reason ___________________________

Total number of Re-operations: ☐

Notes

Signature of person filling the form ________________________ **Date signed** ___/___/___

United Kingdom Collaborative Trial of Ovarian Cancer Screening

UKCTOCS

OVARIAN CANCER DETAILS

First Name________________________ **Surname**_________________________________ **Volunteer Ref No**__________________

Date _________/_________/__________

Please tick the relevant box

Mode of presentation of ovarian cancer
- ❑ Screen detected
- ❑ Symptomatic
- ❑ Incidental finding at op
- ❑ Incidental finding at post-mortem
- ❑ Other

Mode of documentation
- ❑ Slides reviewed
- ❑ Histology/post-mortem report reviewed
- ❑ Death certificate reviewed
- ❑ Clinical opinion + cytology

Ovaries involved at surgery
- ❑ Not involved
- ❑ Right ovary
- ❑ Left ovary
- ❑ Both ovaries

Uterus involved at surgery
- ❑ Not involved
- ❑ Abdominal
- ❑ Pelvic
- ❑ Both Parenchyma

Peritoneum involved at surgery
- ❑ Not involved
- ❑ Abdominal
- ❑ Pelvic
- ❑ Both Parenchyma

Liver involved at surgery
- ❑ Not involved
- ❑ Surface
- ❑ Parenchyma

**Omentum involved in surgery**	YES / NO	**Sigmoid involved in surgery**	YES / NO
**Appendix involved in surgery**	YES / NO	**Spleen involved in surgery**	YES / NO
**Small bowel involved in surgery**	YES / NO	**Diaphragm involved in surgery**	YES / NO
**Colon involved in surgery**	YES / NO	**Rectum involved in surgery**	YES / NO

Maximum dimensions of original disease ____________**mm**

Maximum dimensions of residual disease _____________ **mm**

Stage of ovarian cancer *Ia* *Ib* *Ic* *IIa* *IIb* *IIc* *IIIa* *IIIb* *IIIc* *IV* *NR* (Not recorded)

Chemotherapy given ☐ Not given ☐ Primary ☐ Adjuvant ☐ Other

Number of chemotherapy cycles completed ____

Paclitaxel administered YES / NO **Cisplatin administered** YES / NO **Carboplatin administered** YES / NO
2nd Line chemo administered YES / NO **3rd Line chemo administered** YES / NO

Other chemotherapeutic drugs administered

Primary adjuvant chemotherapy - notes

Any other adjuvant therapy given YES / NO

Interval debulking YES/NO

Number of days admitted in hospice (within time period of 6 months) _____________

Number of times admitted to hospice ____________

Details of other treatment given

Referred to community palliative care team YES / NO

Reason for palliative surgery
- ❑ Bowel Obstruction
- ❑ Fistula
- ❑ Stenting
- ❑ Other

Place of Death
- ❑ Home
- ❑ Nursing home
- ❑ Hospital
- ❑ Hospice

Signature____ **Date** ______________________________

United Kingdom Collaborative Trial of Ovarian Cancer Screening

UKCTOCS

OUTCOMES CANCER REVIEW FORM

First Name ______________________ **Surname** ______________________________ **Volunteer Ref No.** __________________

Cancer **Yes / No**

Primary cancer site:

- ❑ Ovarian or Fallopian tube (C56/C57.0)
- ❑ Primary Peritoneal (C48)
- ❑ Malignant neoplasm, primary site unknown (C80)
- ❑ Malignant neoplasm, primary site unknown BUT NOT OV/FT (C80 but NOT OV/FT/PP)
- ❑ Breast
- ❑ Endometrium
- ❑ Other genital tract
- ❑ Other – specified

Reviewed documentation:

- ❑ Death Certificate
- ❑ ONS Cancer Registration
- ❑ CA125 report
- ❑ Histology Report
- ❑ Cytology Report
- ❑ Histology review
- ❑ Surgery Notes
- ❑ MDT Meeting Summary
- ❑ Hospital Letter/Notes
- ❑ Discharge Summary
- ❑ Imaging Report
- ❑ Hospital Episode Statistics (HES)
- ❑ Other _________________________________

Histology of the ovary/fallopian tube:

- ❑ Primary invasive epithelial malignant neoplasm
- ❑ Primary borderline epithelial malignant neoplasm
- ❑ Primary non-epithelial malignant neoplasm
- ❑ Secondary malignant neoplasm
- ❑ Benign neoplasm
- ❑ Neoplasm of uncertain or unknown behaviour
- ❑ Normal adnexa
- ❑ Other (please describe in notes)
- ❑ No adnexal histology

- ❑ Second adnexal pathology present (details in notes)

Disease code (ICD 10) relating to primary cancer site:

Stage ____________________________

Grade ____________________________

Topography ____________________________

Morphology ____________________________

Additional morphology _____________________

Is this a type II cancer? ☐ Yes ☐ No ☐ Uncertain

Notes:

Synchronous/ Metachronous neoplasm (please circle)

Cancer site ____________________________

Morphology ____________________________

Stage ____________________________

Grade ____________________________

Unable to 'sign off':

- ❑ More information required
- ❑ Outcomes Committee Discussion
- ❑ Path Review

Signature : ___________________________________ **Date:** ______/______/______

Outcomes Committee Form Version 3.8, September 2010
ISRCTN 22488978

Documentation available:		Primary cancer site
Histology definite of ovarian or fallopian tube origin	☐	C56/C57.0
Ovarian/tubal histology suggestive of PP	☐	C48
Histology suggestive of other primary	☐	Other primary

Clinical details	CA125	Adnexal imaging		Imaging		Cytology (ascites)	Pathology		Primary cancer site
Clinical history and/or findings in keeping with OV/FT/PP malignancy	CA125 elevated	Adnexal mass	Normal-sized ovaries	Peritoneal/omental disease in keeping with OV/FT/PP malignancy	Imaging suggests non- OV/FT/PP malignancy	Adenocarcinoma cells	Pathology (any site) compatible with OV/FT/PP malignancy	Pathology (any site) indicates/confirms non-OV/FT/PP malignancy	
☐	☐	☐	☐	☐	☐	☐	☐	☐	
√		√					√		C56/C57
√		√				√			
√		√		√					
√	√			√			√		
√	√			√		√			
√	√		√	√			√		C56/C57/C48
√	√		√	√		√			
√	√		√		√		√		
		√			√				C80 but NOT OV/FT/PP
			√		√				
					√				
	√							√	
√						√ (Malignant cells)			C80

Pink shading: **Irrespective of whether this column is positive or negative**

United Kingdom Collaborative Trial of Ovarian Cancer Screening

OUTCOMES DEATH REVIEW FORM

First Name ______________________ **Surname** ______________________________ **Volunteer Ref No.** __________________

**DC checked**	**Volunteer diagnosed with a cancer previously**	**Outcomes cancer form completed**
Yes / No	Yes / No	Yes / No

Cause of death

- ❑ Primary Ovarian or Fallopian tube
- ❑ Primary Peritoneal
- ❑ Malignant neoplasm, primary site unknown (C80)
- ❑ Malignant neoplasm, primary site unknown BUT NOT OV/FT/PP (C80 but NOT OV/FT/PP)
- ❑ Other cancer – specified
- ❑ Other – non cancerous

If 'Other' please give details and specify ICD-10 code:

If cause of death is OV, FT or PP (please tick ALL applicable fields below):

□ **A. Progression confirmed by**

- ❑ Appearance of new lesions on imaging
- ❑ Increase in the size of previously documented disease on imaging
- ❑ Clinical worsening of the symptoms and signs of disease
- ❑ Rising biomarker levels

□ **B. Treatment complications**

Reviewed documentation for disease progression:

- ❑ CA125 Report
- ❑ Imaging Report
- ❑ Histology Report
- ❑ Cytology Report
- ❑ Surgery Notes
- ❑ MDT Summary
- ❑ Hospital Letter/Notes
- ❑ Hospital Discharge Summary
- ❑ Hospice Discharge Summary
- ❑ Hospital Episode Statistics (HES)
- ❑ Other ________________________________

Unable to 'sign off':

- ❑ More information required
- ❑ Outcomes Committee Discussion
- ❑ Other - specify

Notes:

Signature : ________________________________ **Date:** ______/______/______

Table 1 ICD-10 Codes of Notes Reviewed by the Outcomes Committee

ICD-10 code	Description
C56	Malignant neoplasm of ovary
C57.0	Malignant neoplasm of fallopian tube
C57.4	Uterine adnexa, unspecified
C57.7	Other specified female genital organs
C57.8	Malignant neoplasm of overlapping lesion of female genital organs
C57.9	Malignant neoplasm of female genital organ, unspecified
C48.0	Retroperitoneum
C48.1	Specified parts of peritoneum
C48.2	Malignant neoplasm of peritoneum, unspecified
C48.8	Overlapping lesions of retroperitneum and peritoneum
C76.2	Malignant neoplasm of abdomen
C76.3	Malignant neoplasm of pelvis
C80	Malignant neoplasm without specification of site
D07.3	Carcinoma in situ of other/unspecified female genital organ
D28.2	Benign neoplasm of fallopian tube
D28.9	Benign neoplasm of female genital organ, unspecified
D36.9	Benign neoplasm of unspecified site
D39.1	Neoplasm of uncertain or unknown behaviour of ovary
D39.9	Neoplasm of uncertain or unknown behaviour of female genital organ, unspecified

Abbreviations: ICD, International Classification of Diseases.

Table 2 The morphology of neoplasms (ICD-O) will be used in conjunction with the above ICD-10 codes. The ICD-O codes are relevant to C56 and C57.0.

**Morphology Code**	**Description**
**M8380/3**	Endometriod carcinoma (C56)
**M8381/3**	Endometriod adenofibroma, malignant (C56)
**M8441/3**	Serous cystadenocarcinoma NOS (C56)
**M8442/3**	Serous cystadenoma, borderline malignancy (C56)
**M8450/3**	Papillary cystadenocarcinoma NOS (C56)
**M8451/3**	Papillary cystadenoma, borderline malignancy (C56)
**M8460/3**	Papillary serous cystadenocarcinoma (C56)
**M8461/3**	Serous surface papillary carcinoma (C56)
**M8462/3**	Papillary serous cystadenoma, borderline malignancy (C56)
**M8470/3**	Mucinous cystadenocarcinoma NOS (C56)
**M8471/3**	Papillary mucinous cystadenocarcinoma (C56)
**M8472/3**	Mucinous cystadenoma, borderline malignancy (C56)
**M8473/3**	Papillary mucinous cystadenoma, borderline malignancy (C56)
**M8620/3**	Granulosa cell tumour, malignant (C56)
**M9000/3**	Brenner tumour, malignant (C56)
**M9090/3**	Struma ovarii, malignant (C56)

CAUSES OF CA125 ELEVATION OTHER THAN OVARIAN CANCER (FALSE POSITIVE ELEVATION)

Medical conditions that are known to elevate CA 125 levels:

Colitis

Chronic Active Hepatitis

Cirrhosis

Renal Disease with Serum Creatinine > 2.0

Systemic Lupus Erythematosus

Sarcoidosis

Acute Pancreatitis

Diverticulitis

Endometriosis

Polyarteritis Nodosa

Sjorgrens Syndrome

Pericarditis

Rheumatoid arthritis

Osteoarthritis

Disseminated cancer

UKCTOCS
Suspected Unexpected Serious Adverse Reaction (SUSAR)

Event reported because:

- ❑ *Fatal / Life threatening*
- ❑ *Not part of listed complications*

UKCTOCS ID	PATIENT NAME (CAPITAL LETTERS)	DATE OF BIRTH	HOSPITAL NO
**HOSPITAL**	**REGIONAL CENTRE NO**	**RESPONSIBLE CONSULTANT**	

Please give the **APPROPRIATE MEDICAL TERM** which best describes the event

Onset date___________ **Resolution date**_____________ **Weight** __________ **Height** __________

Report	Category of event	Severity of outcome	Treatment required	Resolution
❑ Initial<br>❑ Follow up	❑ Death<br>❑ Life threatening<br>❑ Hospitalisation (prolongation of)<br>❑ Persistent or significant disability/incapacity	❑ Grade I – Mild<br>❑ Grade II – Moderate<br>❑ Grade III – Severe<br>❑ Grade IV - Life threatening<br>❑ Grade V - Fatal	❑ None<br>❑ Symptomatic<br>❑ Supportive<br>❑ Vigorous supportive	❑ Resolved<br>❑ Resolved with sequelae<br>❑ Improved<br>❑ Unchanged<br>❑ Worsened<br>❑ On-going with sequelae<br>❑ Fatal

Briefly describe the event and treatment given. Include any relevant **MEDICAL HISTORY, ACTION TAKEN** and **FURTHER COMMENTS** (please use additional paper if required)

Date: .... / .... / .... **Lead Researcher's signature**: ....

Please send detailed written report about this event with relevant copies of operation notes, histology, letters and discharge summary to the address below.

To be faxed to Dr Aleksandra Gentry-Maharaj, UKCTOCS Safety Desk: 0207 380 6929

UKCTOCS Coordinating Centre,
Gynaecological Cancer Research Centre,
Institute of Women's Health, UCL
Maple House, 149 Tottenham Court Road,
London, W1T 7NF

Date received by CC:

Signature:

ISRCTN Number 22488978
SUSAR Form
Version 3, September 2004

UKCTOCS

United Kingdom Collaborative Trial of Ovarian Cancer Screening

Second Follow-up Health Questionnaire

Thank you for taking part in UKCTOCS. Please answer every question as fully as you can.

If you are not sure of the exact dates/ details, an approximate answer is better than none.

Please use a **BLACK** pen and where appropriate place a cross [X] inside the box for your answer. Any information that you provide will be treated in the strictest of confidence and will only be used for research purposes. We may contact you if anything is unclear.

We appreciate your time and effort in completing this questionnaire. If possible, please return the questionnaire within 21 days of receipt.

SECTION I: YOUR CONTACT DETAILS

1. **Do you have an email address that we can use to contact you in the future?** Yes ☐ No ☐
   If yes, please provide below. Email address:
   ☐☐☐☐☐☐☐☐☐☐☐☐☐☐☐☐☐☐☐☐☐☐☐☐☐☐☐☐☐☐☐☐☐☐☐☐☐☐

2. **If you have received this questionnaire as redirected mail, please enter your current contact details below so that we can update our records:**
   Address: ....
   Town: .... County: .... Postcode: ☐☐☐☐ ☐☐☐

If your contact details change in the future, please do provide us with your new postal or email address. We will email you the results of the trial when analysis is completed in 2015 if you have an email address.

SECTION II: GENERAL INFORMATION THAT WE DID NOT ASK PREVIOUSLY

1. **Marital Status** (please tick only one box)
   (a) Current:
   ☐ Single ☐ Married ☐ Partnered ☐ Divorced ☐ Separated ☐ Widowed
   (b) Before trial started in 2000
   ☐ Single ☐ Married ☐ Partnered ☐ Divorced ☐ Separated ☐ Widowed

2. **What was your primary occupation?** (Please enter the role in which you have been engaged the longest)
   ....

3. **Please measure and enter the following measurements:**

(a) Your height: ☐ feet ☐☐ inches (b) Your weight: ☐☐ stones ☐☐ lbs

(c) Your waist: ☐☐ inches (d) Your hips: ☐☐ inches

SECTION III: GYNAECOLOGICAL MEDICAL HISTORY – SURGICAL AND INVESTIGATIVE PROCEDURES

Have you had any of the following procedures since joining UKCTOCS?
If yes, please enter the details below. Multiple boxes can be filled; please fill even if you had the procedures as part of the trial.

1. **Operation to look at/remove your ovaries/fallopian tubes** Yes ☐ No ☐
2. **Operation to look into your womb (hysteroscopy), endometrial biopsy (removal of some tissue from your womb) or D & C (scrape of your womb)** Yes ☐ No ☐
3. **Operation to remove your womb (hysterectomy)** Yes ☐ No ☐
4. **Operation to remove part or whole of your breast(s) (e.g. lumpectomy/ wide local excision/mastectomy)** Yes ☐ No ☐

   Any other gynaecological surgery Yes ☐ No ☐
5. **If you have ticked 'Yes' to any of the above, please provide details**

   a) Type of operation: ....

   Date of surgery: ☐☐ / ☐☐ / ☐☐ (dd/mm/yy)

   Hospital Name: ....

   Hospital Number: .... Name of Consultant: ....

   b) Type of operation: ....

   Date of surgery: ☐☐ / ☐☐ / ☐☐ (dd/mm/yy)

   Hospital Name: ....

   Hospital Number: .... Name of Consultant: ....

SECTION IV: YOUR MEDICAL HISTORY

1. **Since joining UKCTOCS have you been diagnosed with any cancer?** *(multiple boxes can be filled)*

   ☐ **I have not been diagnosed with any cancers**

   ☐ Ovarian cancer
   ☐ Breast cancer
   ☐ Cervical cancer
   ☐ Endometrial/womb cancer
   ☐ Vulval/vaginal cancer
   ☐ Bowel/colorectal cancer
   ☐ Gastric/stomach cancer
   ☐ Pancreatic cancer
   ☐ Kidney cancer
   ☐ Bladder cancer
   ☐ Lymphoma
   ☐ Leukaemia
   ☐ BCC/rodent/skin cancer
   ☐ Melanoma
   ☐ Other cancer (please specify below)

   ....

2. **If you have ticked any of the above cancers, please provide details of the main treating hospital where you had your surgery or chemotherapy:**

   a) Type of cancer: .... Date of diagnosis: ☐☐ / ☐☐ / ☐☐ (dd/mm/yy)

   Hospital Name: ....

   Hospital Number: .... Name of Consultant: ....

b) Type of cancer: Date of diagnosis: ☐☐ / ☐☐ / ☐☐ (dd/mm/yy)

Hospital Name:

Hospital Number: Name of Consultant:

3. **Do you have/are being treated for any of the following conditions?** *(multiple boxes can be filled)*

☐ **I do not have any of these conditions**

☐ High blood pressure
☐ Heart disease (e.g. heart attack, angina)
☐ High blood cholesterol
☐ Stroke
☐ Diabetes
☐ Rheumatoid arthritis
☐ Osteoarthritis
☐ Osteoporosis
☐ Thyroid disease
☐ Kidney disease
☐ Inflammatory bowel disease
☐ Liver disease
☐ Chronic Obstructive Pulmonary Disease

4. **If you have ticked any of the above conditions, please provide details of the main treating hospital:**

a) Condition: Date of diagnosis: ☐☐ / ☐☐ / ☐☐ (dd/mm/yy)

Hospital Name:

Hospital Number: Name of Consultant:

b) Condition: Date of diagnosis: ☐☐ / ☐☐ / ☐☐ (dd/mm/yy)

Hospital Name:

Hospital Number: Name of Consultant:

5. **Have you ever taken any of the following medications?**

Tamoxifen	Yes ☐	No ☐	Start date ☐☐ / ☐☐ / ☐☐	Stop date ☐☐ / ☐☐ / ☐☐
Statins	Yes ☐	No ☐	Start date ☐☐ / ☐☐ / ☐☐	Stop date ☐☐ / ☐☐ / ☐☐
Low-dose aspirin	Yes ☐	No ☐	Start date ☐☐ / ☐☐ / ☐☐	Stop date ☐☐ / ☐☐ / ☐☐

SECTION V: ADDITIONAL OVARIAN CANCER SCREENING

1. **Have you had any screening for ovarian cancer OUTSIDE UKCTOCS?** Yes ☐ No ☐

2. **If yes, when was it done and where was this performed?** Date: ☐☐ / ☐☐ / ☐☐ (dd/mm/yy)

☐ NHS Hospital Hospital name:

☐ GP surgery Practice name:

☐ UKCTOCS Trial Centre Name of Centre:

☐ Private Clinic Details of private clinic:

3. **Why was it done?**

☐ Symptoms ☐ Screening ☐ Your request ☐ Other reason (please specify)

4. **If yes, was it abnormal?** Yes ☐ No ☐
Did it result in additional tests? Yes ☐ No ☐
Did it result in surgery? Yes ☐ No ☐
Did it lead to a cancer diagnosis? Yes ☐ No ☐

5. **Since joining UKCTOCS have you had an ultrasound scan?** Yes ☐ No ☐

6. **If yes, when was it done and where was this performed?** Date: ☐☐ / ☐☐ / ☐☐ (dd/mm/yy)

☐ NHS Hospital Hospital name:

☐ GP surgery Practice name:

☐ UKCTOCS Trial Centre Name of Centre:

☐ Private Clinic Details of private clinic:

7. **Why was it done?**

☐ Symptoms ☐ Screening ☐ Your request ☐ Other reason (please specify)

8. **If yes, was it abnormal?** Yes ☐ No ☐
Did it result in additional tests? Yes ☐ No ☐
Did it result in surgery? Yes ☐ No ☐
Did it lead to a cancer diagnosis? Yes ☐ No ☐

SECTION VI: YOUR FAMILY HISTORY OF CANCER

1. **If any of the following relatives have had OVARIAN CANCER please write the number of affected relatives in the appropriate box. Please enter 0 for no affected relatives (e.g. 0 Mother, 2 Sister, 0 Daughter)**

☐ Mother ☐ Daughter ☐ Paternal Aunt ☐ Paternal Grandmother
☐ Sister ☐ Granddaughter ☐ Maternal Aunt ☐ Maternal Grandmother

2. **If any of the following relatives have had BREAST CANCER please write the number of affected relatives in the appropriate box. Please enter 0 for no affected relatives (e.g. 0 Mother, 2 Sister, 1 Daughter)**

☐ Mother ☐ Daughter ☐ Paternal Aunt ☐ Paternal Grandmother
☐ Sister ☐ Granddaughter ☐ Maternal Aunt ☐ Maternal Grandmother

3. **If any of the following relatives have had ENDOMETRIAL CANCER please write the number of affected relatives in the appropriate box. Please enter 0 for no affected relatives (e.g. 0 Mother, 2 Sister, 1 Daughter)**

☐ Mother ☐ Daughter ☐ Paternal Aunt ☐ Paternal Grandmother
☐ Sister ☐ Granddaughter ☐ Maternal Aunt ☐ Maternal Grandmother

Please enter the date you completed this questionnaire: ☐☐ / ☐☐ / ☐☐ (dd/mm/yy)

On completion, please check to make sure you have answered **all** the questions. Please return the questionnaire to us in the **FREEPOST** envelope provided.

Thank you for taking the time to complete this questionnaire. The information that you supply is of great importance to the success of the trial.
