# Supplementary material for: Risk Algorithm Using Serial Biomarker Measurements Doubles the Number of Screen-Detected Cancers Compared With a Single-Threshold Rule in the United Kingdom Collaborative Trial of Ovarian Cancer Screening
Source: J Clin Oncol. 2015 May 11;33(18):2062–71. doi: 10.1200/JCO.2014.59.4945 (PMC4463475; doi:10.1200/JCO.2014.59.4945)
Supplement: Publisher's Note [file supp_33_18_2062_v2_index.html]

Risk Algorithm Using Serial Biomarker Measurements Doubles the Number of Screen-Detected Cancers Compared With a Single-Threshold Rule in the United Kingdom Collaborative Trial of Ovarian Cancer Screening — Serial Biomarker Levels Improve Detection of Ovarian Cancer — Publisher's Note 

# Risk Algorithm Using Serial Biomarker Measurements Doubles the Number of Screen-Detected Cancers Compared With a Single-Threshold Rule in the United Kingdom Collaborative Trial of Ovarian Cancer Screening

## Publisher's Note

The article by Menon et al entitled, "Risk Algorithm Using Serial Biomarker Measurements Doubles the Number of Screen-Detected Cancers Compared With a Single-Threshold Rule in the United Kingdom Collaborative Trial of Ovarian Cancer Screening" (J Clin Oncol 10.1200/JCO.2014.59.4945), was published online May 11, 2015 with an error.

This article is for immediate open access but the online posting did not indicate it. The lead page did not include CC BY license and copyright information, or an icon.

This has been corrected as of May 14, 2015. *Journal of Clinical Oncology* apologizes for the error.
